# The Latest Progress in the Chemistry of *Daphniphyllum* Alkaloids

**DOI:** 10.3390/molecules29235498

**Published:** 2024-11-21

**Authors:** Lujuan Chen, Chao Lv, Yinping Meng, Zhen Yang, Wenbin Xin, Yuxue Zhu, Xuehan Wang, Baozhen Wang, Xuan Ding, Zhaoxia Wang, Xuyue Wei, Xinyue Zhang, Xuexue Fu, Xiangru Meng, Meimei Zhang, Manyu Huo, Ying Li, Hui Yu, Yuxia Wei, Longlong Geng

**Affiliations:** 1 Belgorod Institute of Food Sciences, Dezhou University, Dezhou 253023, China; 2 Shandong Provincial Engineering Research Center of Organic Functional Materials and Green Low-Carbon Technology, School of Chemistry and Chemical Engineering, Dezhou University, Dezhou 253023, Chinallgeng@dzu.edu.cn (L.G.); 3 School of Life Sciences, Dezhou University, Dezhou 253023, China; 4 Health and Medicine College, Dezhou University, Dezhou 253023, China

**Keywords:** *Daphniphyllum* alkaloids, structural determination, biological activity assay, total synthesis

## Abstract

*Daphniphyllum* alkaloids (DAs) are interesting molecules with rich molecular skeletons and diverse biological activities. Since their discovery, phytochemists have isolated, purified, and identified more than 350 DAs. Synthetic chemists, attracted by the structure and activity of DAs, have accomplished many elegant synthetic jobs. Herein, we summarize work on the isolation, structural identification, bioactivity testing, and synthesis of DAs from 2018 to 2023, with the aim of providing a reference for future studies.

## 1. Introduction

The family Daphniphyllaceae, represented solely by the genus *Daphniphyllum*, encompasses approximately 30 plant species primarily found in Southeast Asia. Scientists have isolated various components from *Daphniphyllum* species, including flavonoid glycosides [1,2], triterpene esters [3], phenolic glucosides [4], and alkaloids. The most famous of these are the *Daphniphyllum* alkaloids (DAs). Since their discovery in 1909, over 350 DAs have been identified [5]. These molecules have rich structural diversity and a range of biological functionalities, including cytotoxicity [6], inhibitory activity against kinase enzymes [7], and pesticidal activity against brine shrimp [8,9], among others [10]. The biological properties and structural complexity of DAs have captured the interest of many synthetic chemists. Following the initial report of total synthesis, numerous DAs have been successfully synthesized [11,12,13]. Therefore, while previous reviews have explored various aspects of DAs [5,14,15], in the last five years, a lot of work has been reported on its research, especially synthetic studies, there is a need for an updated report on their isolation, bioactivity, evaluation methods, and synthetic methodologies.

This article provides an updated review of recent advancements in the chemistry of DAs. We begin by introducing new DAs discovered between 2018 and 2023. Following this, we delve into synthetic studies toward DAs, with a particular focus on the synthesis of calyciphylline A-type DAs, along with other significant DAs. Finally, we review the total syntheses of various DAs, highlighting the intricate strategies that have been developed to recreate these complex natural products. By providing a detailed overview of these topics, this review offers valuable insight into the ongoing research and future directions in the field of DAs.

## 2. New DAs and Bioactivity Assays

**Figure 1:**
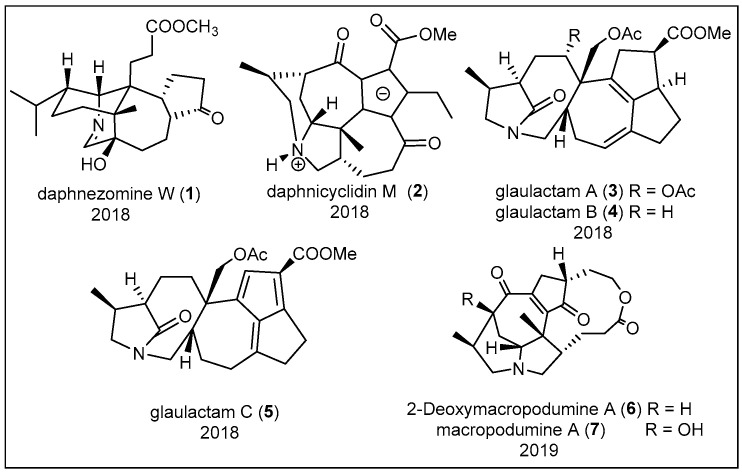
DAs reported in 2018 and 2019.

Several new DAs have been reported in recent years. In 2018, Xiaojiang Hao’s group reported a new daphnezomine L-type DA, daphnezomine W (**1**; Figure 1), which was isolated from the slender leaves of *D. angustifolium* Hutch [16]. Notably, **1** demonstrates moderate cytotoxicity against the HeLa cell line, with a half-maximal inhibitory concentration (IC_50_) of 16.0 μg/mL. Furthermore, the proposed biosynthetic pathway suggests that **1** could be derived from macrodaphniphyllidine via a series of transformations.

Another DA named daphnicyclidin M (**2**; Figure 1) was isolated in 2018 by Li Zhang et al. from the stems and leaves of *D. paxianum* K.Rosenthal [17]. The structure of **2** was elucidated from its spectroscopic data, and its absolute configuration was determined through single-crystal X-ray diffraction. Compound **2** has an interesting skeleton with a rare cyclopentadienyl anion [18]. However, it exhibits no antibacterial activity against various strains [17].

In the same year, Chih-Hua Chao’s group reported three new DAs, glaulactams A–C (**3**–**5**; Figure 1), which were extracted from the leaves of *D*. *glaucescens* Blume [19]. Their structures, including their absolute configurations, were determined using a combination of spectroscopic analyses and time-dependent density-functional-theory-based electronic circular dichroism spectra. The biosynthetic pathways of these DAs are thought to involve transformations from yuzurimine E.

In 2019, Xiaojiang Hao’s group reported a new DA, 2-deoxymacropodumine A (**6**; Figure 1), which was isolated from the stems of *D. angustifolium* [20]. The structure of **6**, including its 11-membered macrolactone ring, was elucidated using techniques such as one- and two-dimensional nuclear magnetic resonance (NMR) spectroscopy and chemical calculations. In addition, by comparing the experimental and calculated NMR data of **6** and macropodumine A (**7**; Figure 1), the structure of **7** was revised owing to its structural similarity to **6** [21,22]. Specifically, both **6** and **7** possess unusual 11-membered macrolactone rings. The proposed biosynthetic pathway for **6** suggests that it originates from 22-norcalyciphylline A-type alkaloids. Furthermore, it demonstrates moderate cytotoxicity against HeLa cells with an IC_50_ of approximately 3.89 μM.

**Figure 2:**
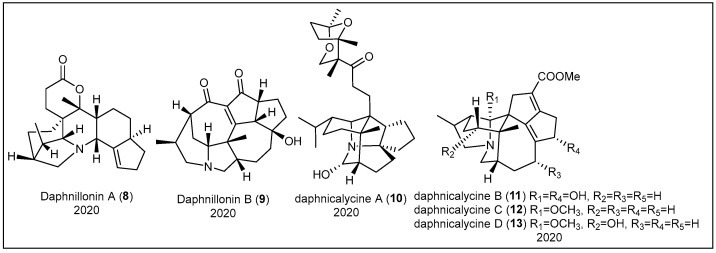
DAs reported in 2020.

In 2020, Yue’s group discovered and characterized two highly rearranged DAs, daphnillonins A and B (**8** and **9**; Figure 2), from *D. longeracemosum* K.Rosenthal [23]. Compound **8** exhibits a distinctive 8-methyl-6-azabicyclo[3.2.1]octane moiety, whereas **9** features an unusual 7/6/5/7/5/5 fused ring structure. Their structures were determined by techniques including electronic circular dichroism calculations. Compound **8** is hypothesized to originate from the coexisting secodaphniphylline-type alkaloids, whereas **9** might originate from the transformation of daphniyunnine A. However, **8** and **9** do not exhibit significant HL60 or A549 cell line cytotoxicity, anti-*Helicobacter pylori* activity, immunosuppressive effects, or protein tyrosine phosphatase non-receptor type 1 (*PTPN1*) inhibition.

In the same year, Guo’s group isolated and characterized four novel DAs, daphnicalycines A–D (**10**–**13**; Figure 2), from the foliage and stems of *D. calycinum* Benth. [24]. Their structures were determined through comprehensive spectral analyses and X-ray crystallography. The researchers also clarified the structure of caldaphnidine E [25] and provided complete ^1^H and ^13^C NMR assignments of daphniteijsmanine [25,26]. Unfortunately, none of these compounds demonstrated significant inhibition of lipopolysaccharide-induced macrophage inflammation at a concentration of 10 μM.

**Figure 3:**
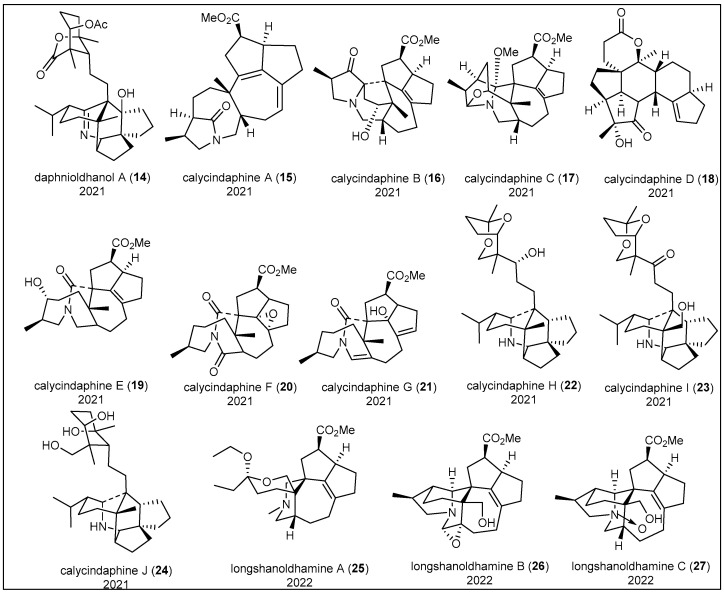
DAs reported in 2021 and 2022.

In 2021, Hao’s group isolated a new DA, daphnioldhanol A (**14**; Figure 3), from the stems of *D*. *angustifolium* [27]. Compound **14**, which is a secodaphnane-type alkaloid, demonstrates weak cytotoxicity against the HeLa cell line with an IC_50_ of 31.9 μM. The researchers hypothesized that **14** is biosynthetically derived from squalene in plants.

In the same year, Zhu and colleagues isolated ten novel DAs, calycindaphines A–J (**15**–**24**; Figure 3), from the roots of *D*. *calycinum* [28]. Their chemical structures were determined using advanced spectroscopic techniques and cross-referencing with published data. Compound **15** has an unprecedented structure, whereby the C_22_ skeleton features a unique 5/8/7/5/5 ring system. Compound **16** is the second reported instance of a calyciphylline G-type alkaloid, whereas **24** is the first reported secodaphniphylline-type alkaloid without an oxygen bridge between the C25 and C29 atoms. Furthermore, potential biogenetic pathways for **15** and **16** have been proposed. Compounds **15**–**24** were assessed for their bioactivities in three cellular models; however, no bioactivities were identified.

In 2022, Guo’s group isolated three previously unreported DAs, longshanoldhamines A–C (**25**–**27**; Figure 3), and two undescribed triterpenoids from the fruits of *Daphniphyllum oldhamii* (Hemsl.) K.Rosenthal [29]. Their structures were determined through comprehensive spectroscopic analyses and X-ray diffraction, as well as comparisons with reported data.

**Figure 4:**
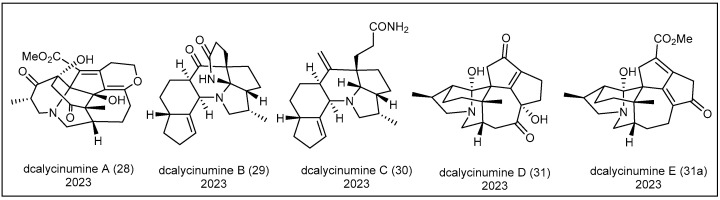
DAs reported in 2023.

In 2023, five novel DAs, i.e., dcalycinumines A–E (**28**–**31a**; Figure 4), were isolated from *D. calycinum* [30]. Compound **28** is the first DA with a 6/6/6/7/5/6 hexacyclic architecture [30], whereas **29** is an uncommon diamino DA featuring a previously unseen carbon framework. Compounds **31** and **31a** are two novel examples of the C_22_-noryuzurimine-type alkaloids. Notably, **28** exhibited significant antitumor activities, including inhibition of the proliferation, migration, and invasion of nasopharyngeal cancer cells, as well as the promotion of apoptosis.

## 3. Synthetic Strategies Toward DAs

Owing to intense research on the active compounds in *Daphniphyllum* species, reports on the isolation of DAs have gradually decreased in recent years. Current research in this field is mainly focused on the synthesis of reported molecules. Indeed, many organic chemists have been attracted to explore the synthesis of DAs because of their rich and complex skeleton types and diverse biological activities. This section discusses synthetic strategies for the synthesis of DAs.

### 3.1. Research on the Synthesis of Calyciphylline A-Type Alkaloids

#### 3.1.1. Synthesis of 7/5/6/5 Tetracyclic Carbon Core of Logeracemin A

In 2014, Yue’s group isolated a dimeric calyciphylline A-type alkaloid, logeracemin A, from *D. longeracemosum* [31]. Logeracemin A exhibits significant anti-HIV activity with a half-maximal effective concentration (EC_50_) of 4.5 ± 0.1 μM. Its interesting structure and bioactivity attracted the attention of Xu’s group, who reported a concise synthetic route for the 7/5/6/5 all-carbon ring system at its core [32].

**Scheme 1:**
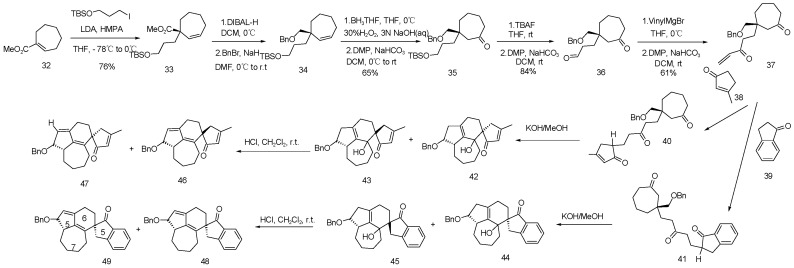
Synthesis of the 7/5/6/5 tetracyclic carbon core of logeracemin A.

As depicted in Scheme 1, the synthesis commenced by functionalizing a commercially available compound, methyl cyclohept-1-ene carboxylate (**32**), using lithium diisopropylamide (LDA) and an iodide derivative to introduce an alkyl tether and thus form cycloheptene derivative **33**. Subsequent reduction of the methyl ester group, followed by protection of the resulting alcohol with a benzyl group, led to the generation of **34**. Regioselective hydroboration–oxidation of **34** utilizing BH_3_ yielded a mixture of alcohol isomers [33]. This mixture was further subjected to Dess–Martin periodinane (DMP) oxidation to obtain ketone **35**. Deprotection of the *tert*-butyldimethylsilyl (TBS) group in **35** and oxidation of the primary alcohol moiety resulted in aldehyde formation (**36**). Subsequently, regioselective Grignard addition between the aldehyde group and another reagent at −78 °C, followed by Dess–Martin oxidation, transformed the resultant alcohol into an intermediate cycloheptanone product (**37**). Intermediate **37** then underwent Michael addition with a ketone (**38** or **39**) to form 7/5/6/5 tetracyclic β-hydroxy ketones (**42**–**45**). The reaction proceeded via an intermediate (**40** or **41**), with successive Michael addition and double aldol reactions to generate the spiro-linked framework. The final step involved the elimination of H_2_O from **42**–**45** to produce the 7/5/6/5 all-carbon structure (**46**–**49**). This biomimetic strategy successfully constructed the complete tetracyclic carbon framework found in logeracemin A, but there was a problem of poor stereoselectivity.

#### 3.1.2. Synthesis of ABC Tricyclic Core of 21-Deoxymacropodumine D

**Scheme 2:**
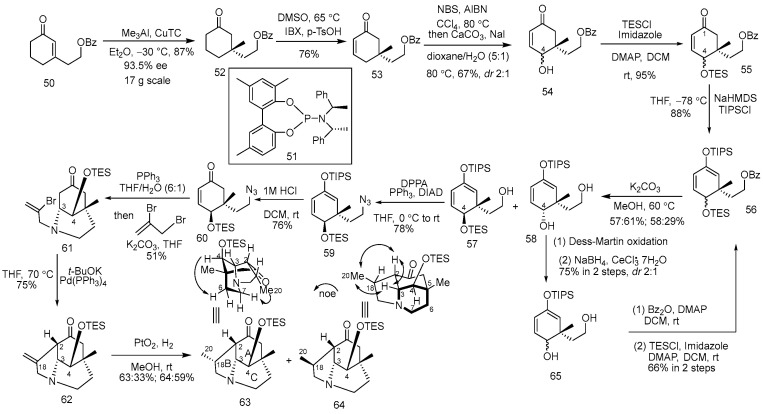
Synthesis of the ABC tricyclic core of 21-deoxymacropodumine D.

The asymmetric synthesis of the ABC tricyclic core of 21-deoxymacropodumine D was reported by Qin’s group in 2018 [34]. In this synthesis (Scheme 2), a benzoyl (Bz)-protected α,β-unsaturated ketone (**50**) was converted to adduct **52** at −30 °C in the presence of trimethylaluminum (Me_3_Al) and copper(I) thiophene-2-carboxylate (CuTC) in diethyl ether (Et_2_O) by employing ligand **51** [35]. This reaction afforded a high yield of 87% and excellent enantioselectivity (enantiomeric excess (ee): 93.5%). The enantioenriched ketone **52** was oxidized to form α,β-unsaturated ketone **53** using 2-iodoxybenzoic acid (IBX)/*p*-toluenesulfonic acid (*p*-TsOH). Subsequently, allylic bromination with *N*-bromosuccinimide (NBS)/azobisisobutyronitrile (AIBN) was conducted at 80 °C, followed by a reaction with CaCO_3_/NaI and then hydrolysis using dioxane/H_2_O to obtain allyl alcohol derivative **54**. The protection of **54** was achieved through treatment with triethylsilyl chloride (TESCl)/imidazole/4-dimethylaminopyridine (DMAP), resulting in the formation of a protected intermediate **55**. Diene compound **56** was then obtained using triisopropylsilyl chloride (TIPSCl)/sodium bis(trimethylsilyl)amide (NaHMDS) under cryogenic conditions (−78 °C). Hydrolysis using K_2_CO_3_ in methanol (MeOH) led to benzoate formation and subsequent conversion into an alcohol intermediate (**57** or **58**). The intermediate **58** was oxidized and reduced to obtain **65**, and the hydroxyl group of **65** was protected to obtain **56**. Compound **57** was then treated with diphenyl phosphate azide (DPPA) in tetrahydrofuran (THF), resulting in the formation of azide derivative **59** in 78% yield. Substrate **59** underwent treatment with 1 M HCl in dichloromethane (DCM), leading to selective desiliconization to form azide **60** in 76% yield. The azide group of substrate 60 was reduced to an amino group followed by cascade aza-Michael addition, and then through alkylation, ultimately forming an alkyl azabicyclo[3.3.1] framework (**61**) in 51% yield. Treatment of **61** with tetrakis(triphenylphosphine)palladium(0) (Pd(PPh_3_)_4_) and potassium *tert*-butoxide (*t*-BuOK) in THF at an elevated temperature [36] resulted in Pd-catalyzed α-alkenylation, leading to the synthesis of a novel C_2_ stereocentric bowl-shaped tricyclic product (**62**) in 75% yield. Compounds **63** and **64** were obtained through the catalytic hydrogenation of **62**.

#### 3.1.3. Synthesis of ACDE Ring System of Calyciphylline A-Type Alkaloids via [5+2] Cycloaddition

**Scheme 3:**
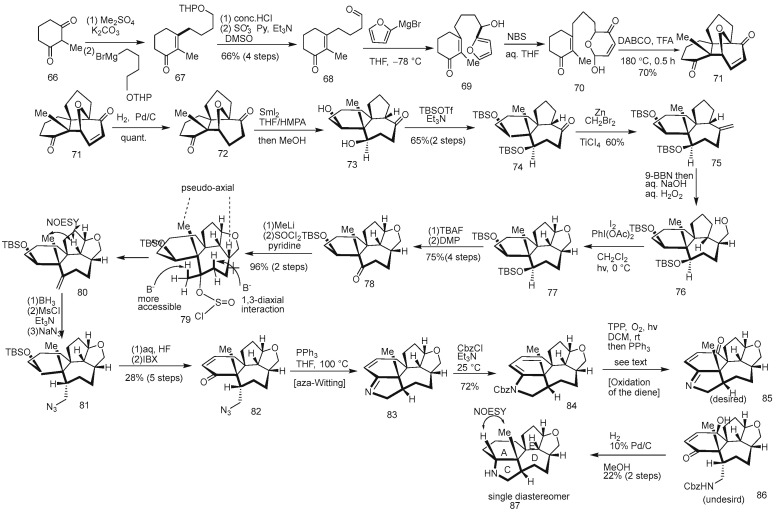
Synthesis of the ACDE ring system of calyciphylline A-type alkaloids via [5+2] cycloaddition.

The ACDE ring framework found in calyciphylline A-type alkaloids was effectively synthesized by Takemoto’s group in 2019 [37]. As depicted in Scheme 3, the synthesis involved an intramolecular reaction using a tetrasubstituted olefin-containing oxidopyrylium species, resulting in [5+2] cycloaddition. First, a cyclization precursor (**70**) was synthesized from 2-methylcyclohexane-1,3-diketone (**66**) via the formation of a vinyl methyl ester with K_2_CO_3_ and dimethyl sulfate (Me_2_SO_4_), followed by nucleophilic addition with a Grignard reagent [38] and acidic treatment to obtain α,β-unsaturated ketone **67**. After the removal of the tetrahydropyranyl (THP) group of **67** and oxidization to form aldehyde **68**, a reaction with 2-furanyl magnesium bromide resulted in alcohol **69**. NBS was employed to obtain **70** from **69** [39]. Finally, **70** underwent [5+2] cyclization to obtain tricyclic compound **71** in 70% yield.

Subsequently, **71** was converted to **73** via **72** by hydrogenation and a reductive ring-opening reaction using SmI_2_ [40], TBS protection of **73** giving **74**, and then **75** was prepared in 60% yield via the Takai reaction [41] from **74** to introduce an exosubunit. The treatment of **75** with 9-borabicyclo[3.3.1]nonane (9-BBN) followed by NaOH/H_2_O_2_ produced alcohol **76**, which was converted to tetracyclic compound **77** by C–H oxidation using I_2_ and phenyliodine(III) diacetate (PIDA) under light irradiation [42]. Then, one of the protecting groups of **77** was removed and oxidized with DMP to obtain ketone **78**. Ketone **78** was treated with methyllithium (MeLi) and SOCl_2_ to deliver the external methylene compound **80**. Next, compound **80** underwent a process of borohydride oxidation, mesylate esterification, and nucleophilic substitution to produce azide **81**. The TBS group of **81** was removed using an aqueous solution of HF and direct oxidation with IBX [43] to produce enone **82**. Reduction of the azide groups using triphenylphosphine (PPh_3_) at 100 °C produced amine **83**, which was then converted to **84** using benzyl chloroformate (CbzCl) and triethylamine (Et_3_N). Under light irradiation and in the presence of catalytic tetraphenylporphyrin (TPP) [44], a facile [4+2] cycloaddition reaction occurred between **84** and O_2_, resulting in an unstable peroxide. Treatment of this peroxide with dimethyl sulfide (Me_2_S) afforded a mixture of the desired product (**85**) and an undesired ring-opened product (**86**). Direct hydrogenation using Pd/C resulted in the exclusive formation of pentacyclic compound **87** as a single diastereomer. Thus, model compound **87** was produced through a 27-step reaction from commercially available **66**.

#### 3.1.4. Synthesis of Tricyclic Spiro-Ring Structure of Calyciphylline A-Type Alkaloids

**Scheme 4:**
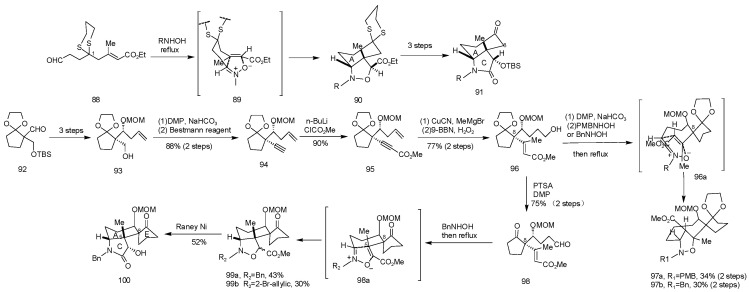
Synthesis of the tricyclic spiro-ring structure of calyciphylline A-type alkaloids.

In 2019, Gao’s team investigated 1,3-dipolar cycloaddition reactions with the aim of synthesizing the central tricyclic spiro-ring structure of calyciphylline A-type alkaloids [45]. As depicted in Scheme 4, they observed that substrate **88**, which featured a 1,3-dithiane group capable of interacting with various hydroxylamines, facilitated the formation of the desired cycloadduct **90** via the 6-*endo* transition state **89** [46]. After reductive cleavage and subsequent lactamization, **90** was converted to *cis*-hydroindole (**91**).

Next, aldehyde **92** was converted to a methoxymethyl (MOM)-protected alcohol (**93**) in three steps. Compound **93** was transformed into alkyne **94** with an 88% yield over two steps. Subsequently, **94** was reacted with methyl chloroformate (ClCO_2_Me) under the influence of *n*-butyllithium (*n*-BuLi), resulting in the formation of **95**. The two-step treatment of **95** afforded alcohol **96** in 77% yield. Next, **96** underwent oxidation and subsequent condensation with *N*-methoxybenzylhydroxylamine or benzylhydroxylamine, followed by heating to achieve 1,3-dipolar cycloaddition, resulting in **96a** and then obtained **97a** and **97b**. The structure and stereochemistry of cycloadducts **97a** and **97b** were verified via X-ray crystallography.

To further enhance the stereoselectivity, the researchers modified the structure of **96** by removing the 1,3-dioxolane-protecting group under acidic conditions. The resulting product was directly oxidized to aldehyde **98** in 75% yield over two steps. Aldehyde **98** was then condensed with different hydroxylamines under identical conditions, resulting in the formation of the corresponding nitrones. These nitrones then underwent 6-*endo* cycloaddition via **98a** to afford **99a** and **99b** in moderate yield. The N–O bond of isoxazolidine **99a** was selectively cleaved through a reductive reaction using Raney Ni, followed by spontaneous lactamization to produce tricyclic product **100** in 52% yield. The formation of the 5/6/5 ACE tricyclic spiro-ring was confirmed via single-crystal X-ray diffraction.

#### 3.1.5. Synthesis of 6/5/7/5 Tetracyclic Core of Calyciphylline A-Type Alkaloids

**Scheme 5:**
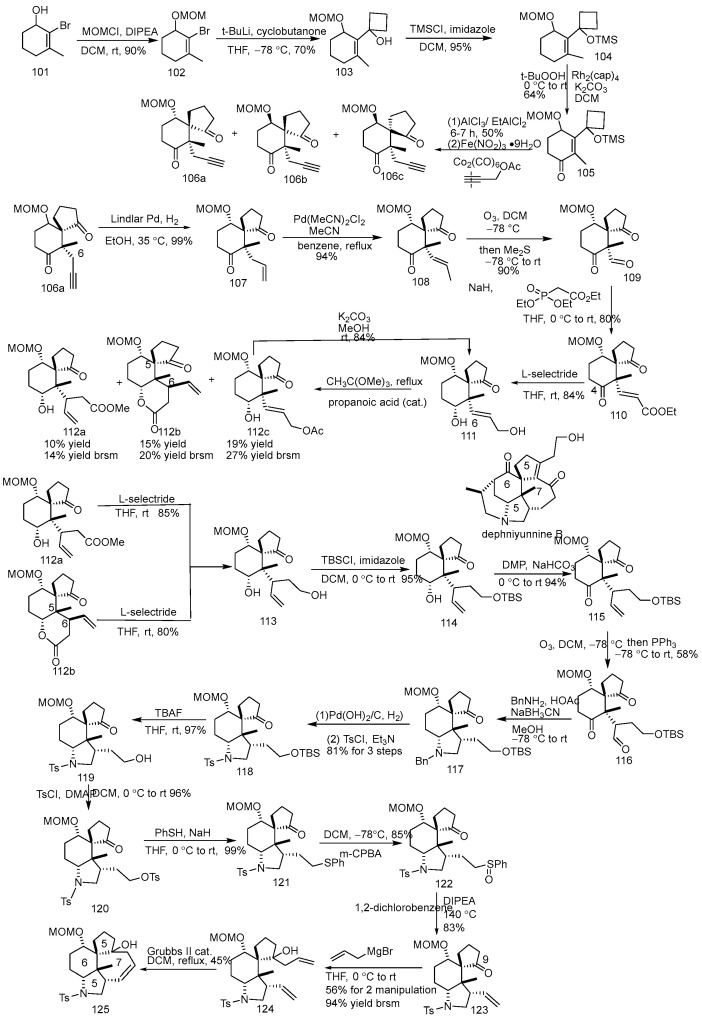
Synthesis of the 6/5/7/5 tetracyclic core of calyciphylline A-type alkaloids.

Tu’s team synthesized the 6/5/7/5 tetracyclic core of calyciphylline A-type alkaloids via a sequential semipinacol rearrangement and Nicholas reaction [47]. As depicted in Scheme 5, the synthesis of enone **105** was initiated from the well-established allyl alcohol **101**. MOM ether was used to protect the secondary hydroxy group of **101**, resulting in the formation of **102** in high yield (90%). Compound **102** underwent Li–halogen exchange with *tert*-butyllithium (*t*-BuLi), followed by the addition of cyclobutanone to produce the desired product (**103**). Compound **103** underwent silylation using trimethylsilyl chloride (TMSCl), and then Doyle’s conditions [48] were employed for allylic oxidation to generate rearrangement precursor **105**. Compound 105 underwent the crucial tandem semipinacol rearrangement/Nicholas reaction yield **106a**, **106b**, and **106c**. The structures of **106a**, **106b**, and **106c** were confirmed by X-ray crystallography. Importantly, the relative configurations between C5 and C8 in **106a** and **106b** were both consistent with those of daphniyunnine B.

The researchers opted to elaborate further on the major isomer **106a**. The alkynyl group of **106a** was partially reduced to yield alkene **107** in quantitative yield using Lindlar Pd and H_2_. Subsequently, **107** underwent isomerization using bis(acetonitrile)palladium dichloride (PdCl_2_(MeCN)_2_) to form internal olefin **108**. The synthesis of α,β-unsaturated ester **110** was achieved in satisfactory yield (72%) through the ozonolysis of **108** followed by the Horner–Wadsworth–Emmons (HWE) reaction with aldehyde **109**. The distal ester group in **110** was subsequently reduced using L-selectride, resulting in allylic alcohol **111** with an impressive yield (84%). Notably, when treated with trimethyl orthoacetate (CH_3_C(OMe)_3_) and catalytic propanoic acid at 130 °C, **111** yielded C6-vinylated products **112a** and **112b**, although their combined yield remained low at only 25%. An acetyl-protected allylic alcohol (**112c**) emerged as a significant byproduct during this reaction; however, it was effectively recycled by hydrolysis.

The reduction of methyl ester **112a** and lactone **112b** using L-selectride resulted in high yields of 85% and 80%, respectively, for the formation of diol **113**. TBS silyl ether **114** was employed to selectively protect the primary hydroxyl group in **113**, while the secondary hydroxyl group underwent oxidation using DMP to produce diketone **115**. By subjecting **115** to ozonolysis, aldehyde **116** was obtained in 52% overall yield. The reaction proceeded favorably when aldehyde **116** was treated with benzylamine (BnNH_2_) in the presence of cyanoborohydride (NaBH_3_CN) and acetic acid (HOAc), resulting in the formation of **117**. Subsequently, high-pressure hydrogenation replaced the *N*-benzyl protecting group in **117** with a 4-toluenesulfonyl (tosyl; Ts) group, producing sulfonamide **118** in 81% overall yield over three steps. After the TBS protective group was removed, compound **119** was obtained. Then, the resulting primary hydroxyl group was sulfonated, and finally, the sulfonate **120** was obtained. The quantitative yield of sulfide **121** was achieved through the nucleophilic substitution of **120** with thiophenol (PhSH), followed by oxidation to form sulfoxide **122**. Compound **122** underwent thermodynamic elimination in the presence of *N*,*N*-diisopropylethylamine (DIPEA) [49], thus forming terminal olefin **123**. By adding allylmagnesium bromide (allylMgBr) to the C9 ketone group of olefin **123**, diene **124** was obtained via Grignard addition. Finally, the 6/5/7/5 tetracyclic framework, resembling that of calyciphylline A, was formed through intramolecular ring-closing metathesis using Grubbs second-generation (Grubbs II) catalyst, leading to the synthesis of **125**.

#### 3.1.6. Synthesis of ACDE Ring System of Calyciphylline A-Type Alkaloids via Intramolecular Diels–Alder Reaction

**Scheme 6:**
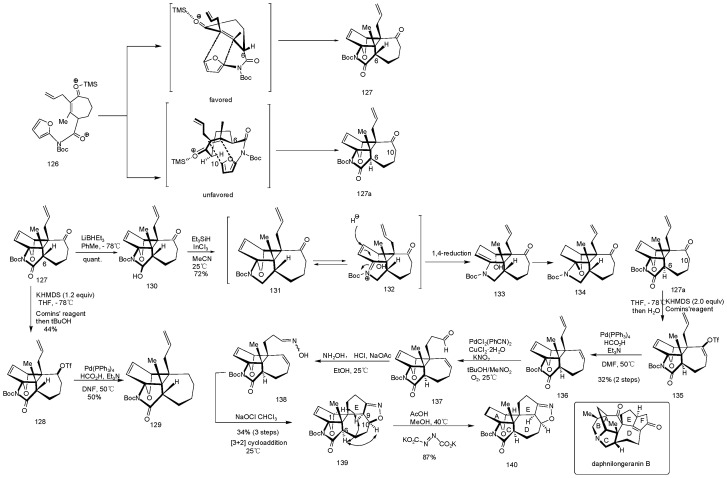
Development of the ACDE ring system of calyciphylline A-like structures via intramolecular Diels–Alder reaction.

The ACDE ring system of calyciphylline A-type alkaloids was synthesized by Takemoto’s group using an intramolecular Diels–Alder reaction involving a retrasubstituted olefin [50]. They first acquired cyclization precursor **126** through a sequence of procedures and then used it to synthesize the A and C rings of calyciphylline A-type alkaloids via an intramolecular Diels–Alder reaction. By thoroughly investigating the effects of different reaction parameters, they achieved the selective formation of diastereomers **127** and **127a** (Scheme 6). Notably, **127** exhibited the desired stereochemistry at position C6.

Next, the researchers focused on constructing the E ring of calyciphylline A-type alkaloids. In the presence of the ketone group, the amide of **127** was selectively reduced upon treatment with 1 equiv of lithium triethylborohydride (LiBHEt_3_), leading to the formation of hemiaminal **130**. Interestingly, when **130** was treated with triethylsilane (Et_3_SiH) and InCl_3_ in acetonitrile (MeCN), **134** was obtained via the reduction of the double bond. By contrast, treating **127** with 1.2 equiv of potassium bis(trimethylsilyl)amide (KHMDS) and Comins’ reagent resulted in trifluoromethanesulfonate (triflate) **128** in 44% yield, without epimerization. Triflate **128** could be converted to triene **129** using Pd(PPh_3_)_4_ and HCOOH in moderate yield (50%). However, **129** displayed inherent instability and decomposed within a short timeframe.

Considering the challenging derivatization of **127**, presumably because of skeletal strain, the researchers investigated the formation of the E ring using thermodynamic Diels–Alder product **127a**, despite its undesired stereochemistry at C6. After the generation of enol triflate **135** from **127a**, a reduction reaction employing Pd(PPh_3_)_4_ and HCOOH was conducted to produce triene **136**. Notably, **136** demonstrated sufficient stability for storage and subsequent utilization. The terminal olefin of triene **136** underwent anti-Markovnikov Wacker oxidation involving bis(benzonitrile)palladium dichloride (PdCl_2_(PhCN)_2_), CuCl_2_·H_2_O, and KNO_2_, resulting in aldehyde **137** in 66% yield. Aldehyde **137** was then treated with NH_2_OH·HCl and sodium acetate (NaOAc), leading to the formation of oxime **138**. A smooth [3+2] cycloaddition reaction occurred in situ upon oxidizing compound **138** with NaOCl, thus affording the ACDE core structure (**139**) in 34% yield. The double bond within the A ring of compound **139** was selectively reduced through reduction, yielding model compound **140** with high efficiency (87% yield).

#### 3.1.7. Construction of ABC Core of Calyciphylline A-Type Alkaloids

**Scheme 7:**
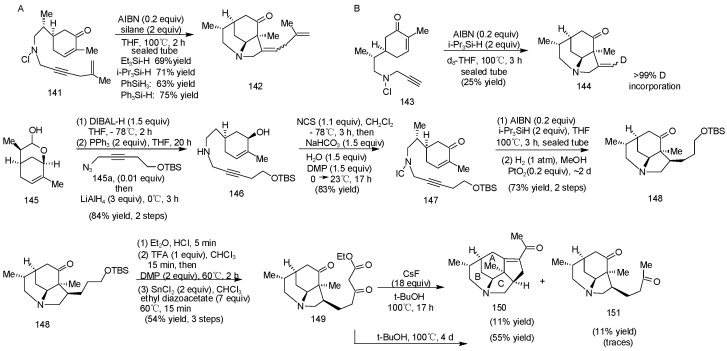
Construction of the ABC core in calyciphylline A-type alkaloids.

In 2018, Stockdill’s team devised an Sn-free approach to effectively cyclize different *N*-chloroamine precursors with internal alkynes [51]. This method facilitated the formation of the ABC core of calyciphylline A-type alkaloids by inducing the cyclization of neutral aminyl radicals. Reactions A and B verified the feasibility of the cyclization reaction. Starting from known compounds, the researchers first prepared cyclization precursors **141** and **143** (Scheme 7). They then treated **141** with AIBN and various silanes to obtain **142**, and **143** was converted to **144**.

The focus of their attention then shifted toward the synthesis of cyclization precursor **149** using the alkynyl azide **145a**, which features a TBS ether. This modification was expected to improve the solubility of the highly polar intermediates. The synthesis of *N*-chloroamine **149** began with a reaction between hemiacetal **145** and azide **145a**. Compound **145** was reduced by DIBAL-H to form an aldehyde, which then reacted with the amine produced by the reduction of compound **145a** to form an imine, followed by the reduction of the resulting imine using LiAlH_4_ to produce amine **146** in impressive yield (84%). Chlorination of **146** with *N*-chlorosuccinimide (NCS) at −78 °C and then further treated with DMP under buffered conditions after gradually increasing the temperature to 0 °C led to the formation of *N*-chloroenone **147**.

*N*-chloroenone **147** underwent tin-free cyclization and then hydrogenated using Adams’ catalyst (20 mol%) yield **148** in a combined isolated yield of 73% over two steps. The Knoevenagel strategy was used to synthesize a β-ketoester from **148** through a three-step procedure without intermediate purification. Desilylation was carried out using concentrated HCl(aq) in Et_2_O. The resulting primary alcohol was then protonated in situ to activate the tertiary amine, followed by oxidation using DMP. Roskamp coupling was achieved by treating **148** with SnCl_2_ and ethyl diazoacetate, yielding **149** in 54% overall yield over three steps. Finally, CsF in *tert*-butanol (*t*-BuOH) was used to convert **149** into the desired 6/6/5/5 ring system (**150**) and trace **151**.

#### 3.1.8. Efficient Synthesis of Tricyclic Scaffold of Calyciphylline A-Type Alkaloids

**Scheme 8:**
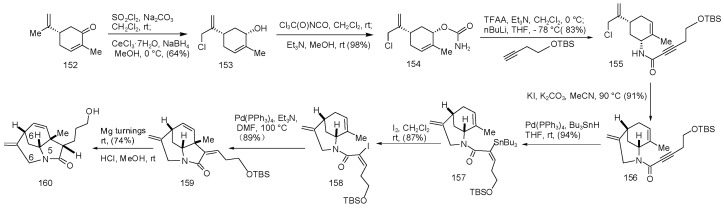
Efficient synthesis of the tricyclic scaffold found in calyciphylline A-type compounds.

Hudlicky and colleagues presented an effective and streamlined method for synthesizing the aza-5/6/6 tricyclic structure of calyciphylline A-type alkaloids by using [1,3]-Ichikawa transposition and intramolecular Heck cyclization [52]. Following strategic planning, the synthesis, as depicted in Scheme 8, was initiated by one-step allylic chlorination followed by Luche reduction of (*S*)-carvone (**152**), resulting in the formation of chloro-carveol **153**. The carbamylation of **153** yielded primary carbamate **154**. After confirming the effectiveness of the Ichikawa transposition-based approach, the researchers effectively captured isocyanate using an alkynyl lithium reagent derived from TBS-protected 3-butyne-1-ol. This resulted in the creation of secondary amide **155**. Heating a mixture of **155**, KI, K_2_CO_3_, and MeCN at 90 °C for two days produced the desired tertiary amide (**156**) in excellent yield. A Pd-catalyzed chemo- and regioselective hydrostannation reaction was then used to generate alkenylstannane **157**, wherein the tributyltin moiety underwent iodination without any complications, yielding vinyl iodide **158**. By heating a mixture of **158**, Pd(PPh_3_)_4_, and Et_3_N in degassed dimethylformamide (DMF) at 100 °C for under 10 min, the desired aza-5/6/6 tricyclic core was formed in excellent yield. The addition of a single drop of concentrated HCl(aq) to a stirred solution of **159** in MeOH, followed by the introduction of Mg turnings in the same reaction vessel, resulted in the production of primary alcohol **160** with saturation occurring specifically at C6–C12.

#### 3.1.9. Synthesis of ABCD Ring System of Calyciphylline A-Type Alkaloids

**Scheme 9:**
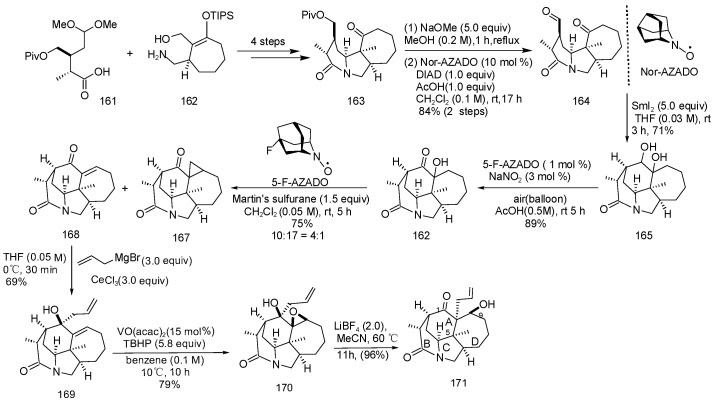
Synthesis of ABCD ring system containing adjacent all-carbon quaternary stergenic centers resembling calyciphylline A-type alkaloids.

The stereocontrolled synthesis of the ABCD tetracyclic ring system of calyciphylline A-type alkaloids, which contains adjacent all-carbon quaternary stereocenters, was accomplished by Iwabuchi’s group in 2018 [53]. As depicted in Scheme 9, compound **163** was obtained from compounds **161** and **162** via four steps [54], and then the synthesis of ketoaldehyde **164** from **163** was achieved through a two-step process, with a high yield of 84%. First, the pivaloyl group was deprotected, and then the resulting alcohol was mildly oxidized using 9-azanoradamantane *N*-oxyl (nor-AZADO) and diisopropyl azodicarboxylate(DIAD) [53].

Subsequently, **164** was used as a substrate for intramolecular pinacol coupling. When SmI_2_ was employed as the reagent, tetracyclic diol **165** was formed as a single diastereomer with an impressive yield (71%) [55]. The secondary alcohol in diol **165** was efficiently oxidized through 5-fluoro-2-azaadamantane *N*-oxyl (5-F-AZADO)/NO*_x_* catalysis and aerobic oxidation [53], leading to α-hydroxyketone **166** in high yield (89%). Importantly, this oxidation procedure effectively preserved the integrity of the vicinal diol moiety. By dehydrating **166** using Martin’s sulfurane reagent, enone **168** was produced along with cyclopropane **167** as an inseparable mixture. The overall yield for this step reached approximately 75%, with a ratio favoring **168** at approximately 4:1 **168**/**167**. Compound **168** was then reacted with allylMgBr in the presence of CeCl_3_ to introduce an allyl group, leading to the formation of allylic alcohol **169** as the sole diastereomer in a 69% yield. The stereocontrolled oxidation of **169** using a V-based catalyst produced epoxy alcohol **170**. Subsequently, **170** was subjected to Marson’s semipinacol rearrangement [56]. Finally, the use of Lipshutz’s conditions, involving LiBF_4_ in MeCN, formed compound **171** as a single stereoisomer with high efficiency.

#### 3.1.10. Production of ABCD Tetracyclic Ring Domain of Calyciphylline A-Type Alkaloids

**Scheme 10:**
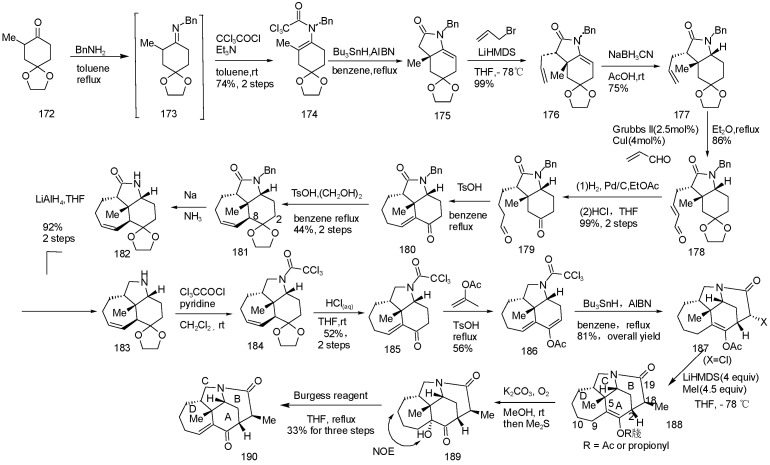
Synthesis of the tetracyclic ABCD ring domain of calyciphylline A-type alkaloids.

The synthesis of the ABCD tetracyclic ring framework of calyciphylline A-type alkaloids was also achieved by Bonjoch’s team using 5-*endo* radical cyclization starting from *cis*-3a-methyloctahydroindole [57,58]. As depicted in Scheme 10, the reaction of ketone **172** with BnNH_2_ using the Dean-Stark apparatus led to the formation of imine **173**, which was subsequently treated with trichloroacetyl chloride (Cl_3_CCOCl) to produce enamide **174**. Trichloroacetamide **174** was then refluxed in benzene and reacted with tributyltin hydride (Bu_3_SnH) or AIBN, leading to the synthesis of hydroindole **175** through 5-*endo*-*trig* cyclization [59]. Enamide **175** underwent allylation using lithium bis(trimethylsilyl)amide (LiHMDS) and allyl bromide at −78 °C, resulting in the diastereoselective formation of **176** in almost complete yield. The acylithinium generated from **176** was selectively reduced using NaBH_3_CN in an acidic medium, thus forming *cis*-octahydroindole **177** [60].

Starting from **177**, α,β-unsaturated aldehyde **178** was obtained through an efficient cross-metathesis reaction using a low-catalyst-loading Grubbs II catalyst and CuI as an additive in Et_2_O. Afterward, the acetal group in the aldehyde was removed using an aqueous solution of HCl in THF, leading to the synthesis of ketoaldehyde **179**. A seven-membered ring was formed through aldol cyclization, employing *p*-TsOH in benzene under reflux conditions. Consequently, the D ring closed to form enone **180** with the ACD tricyclic ring system. Enone was protected using ethylene glycol to yield **181**.

Lactam **181** underwent debenzylation using Na in NH_3_(liq) at −78 °C. This was followed by the reduction of secondary lactam **182** using LiAlH_4_ to achieve a high overall yield of secondary amine **183**. Compound **183** was then trichloroacetylated to obtain **184**, which underwent acid-mediated treatment for the regeneration of moiety **185** containing an α,β-unsaturated ketone. To obtain the required radical acceptor enol acetate **186**, compound **185** was treated with *p*-TsOH and isopropenylacetate. The reaction between enol acetate **186** and Bu_3_SnH, along with AIBN, resulted in **187** with a morphan ring system in up to 82% yield.

In a significant advancement toward the synthesis of valuable intermediates for calyciphylline A-type alkaloids, the diastereoselective alkylation of lactam **187** introduced a methyl substituent with an identical configuration as that observed in the natural target product. Surprisingly, upon the deprotection of enol acetate **188** using K_2_CO_3_ in MeOH, α-hydroxylated ketone **189** was isolated instead of a simple ketone [61]. Finally, the Burgess reagent was employed to dehydrate the tertiary alcohol in **189**, affording **190** in modest yield (33% over three steps; 70% achieved for each individual chemical event from **188**).

#### 3.1.11. Synthesis of AC Bicyclic Framework of Daphniyunnine B

**Scheme 11:**
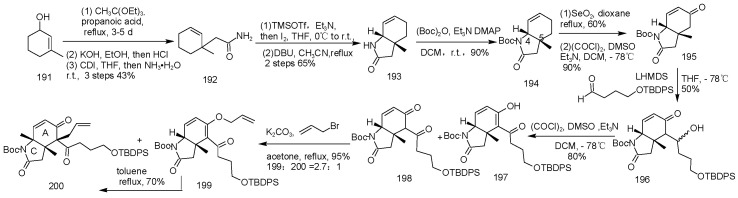
Synthesis of the AC bicyclic framework of daphniyunnine B.

In 2019, Xie’s group constructed the AC ring system of daphniyunnine B [62]. As depicted in Scheme 11, the synthesis began with the acid-promoted rearrangement, hydrolysis, and amidation of cyclohexenol **191** to obtain primary amide **192**, with a single purification step using column chromatography. Subsequently, bicyclic lactam **193** was synthesized through intramolecular iodocyclization under Levorse’s conditions [63], followed by 1,8-diazabicyclo[5.4.0]undec-7-ene (DBU)-promoted elimination. Compound **194** was then protected to achieve a yield of 90%. Its cis configuration was confirmed through nuclear Overhauser effect experiments on fused bicyclic lactam. Finally, high yields of bicyclic lactam **195** were obtained through SeO_2_-assisted allylic oxidation and Swern oxidation of the diastereomeric secondary alcohols.

Following the synthesis of **195**, the researchers turned their focus to the synthesis of the C8 quaternary stereocenter of the AC ring system. To achieve this, they combined **195** with an aldehyde by aldol reaction, resulting in the formation of β-hydroxyl ketone **196**. Subsequently, **196** was converted to a more reactive form, 1,3-diketone **198**, which coexisted with its enolization isomer **197**. Both compounds underwent allylation, leading to a mixture of O-alkylation product **199** and C-alkylation product **200** in a ratio of approximately 2.7:1 (total yield of up to 95%). Finally, subjecting **199** to heat-promoted Claisen rearrangement facilitated its transformation into **200** with approximately 70% yield.

#### 3.1.12. Construction of ABC Ring System of 21-Deoxymacropodumine

**Scheme 12:**
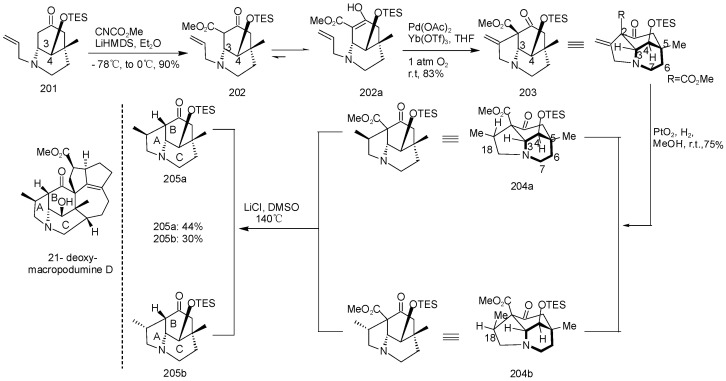
Construction of ABC ring of 21-deoxymacropodumine.

In 2020, Tang’s group reported the synthesis of the ABC tricyclic ring system of 21-deoxymacropodumine [64]. As depicted in Scheme 12, the reactive deprotonation of **201** using LiHMDS, followed by quenching of the resulting anion with Mander’s reagent, resulted in the formation of enol **202a** as the major product. Next, **202a** was treated with palladium(II) acetate (Pd(OAc)_2_) and ytterbium(III) triflate (Yb(OTf)_3_) in THF under an O_2_ atmosphere for Pd-catalyzed intramolecular oxidative alkylation. This led to the synthesis of C2–C18 ligated compound **203**. The double bond in **203** was hydrogenated catalytically using PtO_2_ in MeOH, resulting in a mixture of two diastereomers (**204a** and **204b**) at a ratio of 1.5:1. The relative stereochemistry between **204a** and **204b** was determined by comparing their respective NMR spectra with those of Krapcho decarboxylation products **205a** and **205b**.

### 3.2. Research on the Synthesis of Other Calyciphylline-Type Alkaloids

#### 3.2.1. Synthesis of the ABE Tricyclic Core of Calyciphylline B-Type Alkaloids

**Scheme 13:**
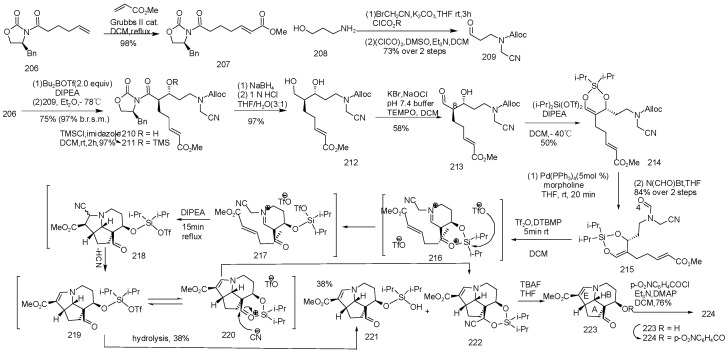
Synthesis of the ABE tricyclic core of calyciphylline B-type alkaloids.

Boissarie and Bélanger presented a concise method for synthesizing the enantiomerically enriched ABE tricyclic scaffold found in calyciphylline B-type alkaloids [65]. As depicted in Scheme 13, a reaction involving **206** and methyl acrylate resulted in the production of unsaturated ester **207** via cross-metathesis [66]. Subsequently, **207** underwent a Heathcock aldol reaction [67] with aldehyde **209**, leading to the satisfactory formation of anti-product **210**. The direct reduction of **210** proved challenging. Thus, a trimethylsilyl (TMS) group was introduced to protect the free alcohol, affording **211**. The protecting group was removed during acid quenching in the following reduction reaction, resulting in diol **212** with a nearly complete yield for these two steps.

To selectively oxidize the primary alcohol moiety, catalytic 2,2,6,6-tetramethylpiperidin-1-yl)oxyl (TEMPO) and sodium hypochlorite (NaOCl) were employed as co-oxidants to obtain compound **213** followed by enolization into cyclic silyl enol ether **214**. The conversion of **214** through deallyloxycarbonylation [68], followed by immediate formylation using Katritzky salts [69], yielded polycyclization precursor **215**. Compound **215** was exposed to amide activation conditions (triflic anhydride (Tf_2_O) and 2,6-di-*tert*-butylpyridine (DTBMP)), resulting in smooth and rapid Vilsmeier–Haack cyclization to form iminium ion **216**. By transferring the reaction solution to a flask containing a refluxing solution of DIPEA in DCM, efficient cycloaddition also ensued. Encouragingly, the excess DIPEA utilized for azomethine ylide generation also facilitated cyanide elimination. Consequently, two cycloadducts were obtained in equal proportions: the expected tricyclic aldehyde **221** and a tetracyclic silylated cyanohydrin **222**.

The researchers proposed that these products were formed by the partial conversion of silyl triflate **219** into cyclic oxocarbenium ion **220**. Ion **220** then reacted with the cyanide that formed during the elimination process to afford cyanohydrin **222**, whereas hydrolysis of the remaining **219** produced aldehyde **221** during the aqueous quench. Importantly, both compounds can be desilylated independently using tetra-*n*-butylammonium fluoride (TBAF) to obtain the desired tricyclic product (**223**) in satisfactory yield. By employing a one-pot procedure involving sequential Vilsmeier–Haack and cycloaddition reactions followed by TBAF treatment, **224** was obtained from substrate **223** in an impressive overall yield of approximately 69%.

#### 3.2.2. Synthesis of ABCE Tetracyclic Core of Calyciphylline B-Type Alkaloids

**Scheme 14:**
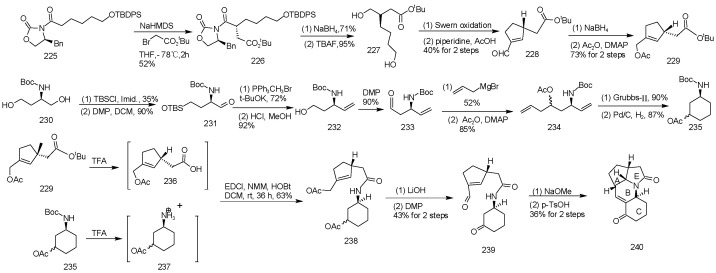
Synthesis of the ABCE tetracyclic core found in calyciphylline B-type alkaloids.

She’s group developed a novel approach involving two cyclization steps to obtain the tetracyclic core found in calyciphylline B-type alkaloids [70]. By starting with ε-caprolactone, they synthesized chiral oxazolidinone **225** using established methods [71]. They then proceeded with an asymmetric alkylation using *tert*-butyl bromoacetate at low temperature, resulting in the formation of **226** with exceptional diastereoselectivity (Scheme 14) [72]. Removal of the Evans auxiliary and desilylation with TBAF resulted in diol **227**. The subsequent oxidation of **227** afforded the corresponding dialdehyde without purification. This dialdehyde underwent intramolecular condensation to generate a chiral cyclicenal intermediate **228** [73,74]. Finally, fragment **229** was obtained in high yield by reducing the aldehyde group to an alcohol and subsequently protecting it with an acetyl group.

After preparing fragment **229**, the researchers then focused on synthesizing fragment **235**. To achieve this, diol **230** was synthesized from d-aspartic acid following an established protocol. The remaining hydroxyl groups were selectively protected with *tert*-butyldimethylsilyl chloride (TBSCl) and then oxidized to form aldehyde **231**. Introducing a terminal olefin through Wittig methylenation followed by acid-assisted deprotection resulted in the release of free alcohol **232**, which was readily converted to aldehyde **233** using DMP. Next, the nucleophilic addition of allylMgBr to the aldehyde group generated a secondary alcohol that underwent acylation with acetic anhydride (Ac_2_O). This reaction produced linear diene **234** as the final product. The ring-closing metathesis of alkene **234** in the presence of Grubbs II catalyst resulted in the corresponding cyclohexene. Subsequently, hydrogen saturation was used to form fragment **235**.

With sufficient quantities of fragments **229** and **235** at hand, the subsequent step involved their coupling to investigate cyclization reactions. The compounds **236** and **237** were obtained by stirring **229** and **235** in DCM with excess trifluoroacetic acid (TFA) to liberate the carboxylic acid and amine, respectively, for direct utilization in the subsequent step. After multiple trials, it was found that a mixture of 1-ethyl-3-(3-dimethylaminopropyl)carbodiimide (EDCI) and hydroxybenzotriazole (HOBt) in *N*-methylmorpholine (NMM) facilitated intermolecular amidation, leading to the formation of the coupling product **238** [75]. The base-promoted hydrolysis and double oxidation of **238** converted its two acetoxyl groups into carbonyl ones to form **239**. Finally, **240** was formed through intramolecular aldol condensation to close the central ring of tetrahydropyridine B [76]. The cyclization required a stoichiometric quantity of *p*-TsOH, as confirmed by control experiments. Notably, this synthesis of the ABCE ring system in **240** is a promising step toward the complete synthesis of calyciphylline B-type alkaloids.

#### 3.2.3. Synthesis of Aza-5/7/6/7 Tetracyclic Core of Calyciphylline D-Type Alkaloids

**Scheme 15:**
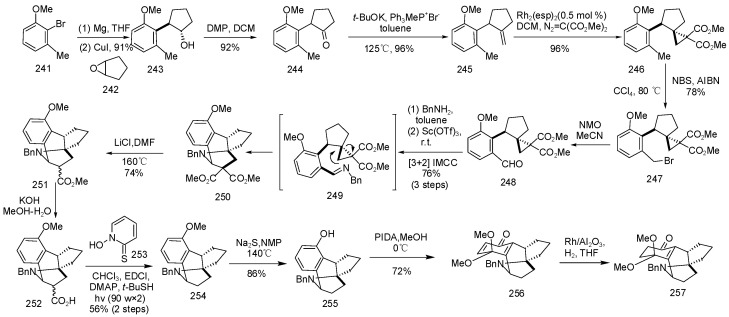
Synthesis of a tetracyclic core structure with an aza-5/7/6/7 motif resembling calyciphylline D-type alkaloids.

In 2021, Wang’s group documented the synthesis of a tetracyclic core structure featuring an aza-5/7/6/7 configuration similar to that in calyciphylline D-type alkaloids [77]. As depicted in Scheme 15, the synthesis was initiated using known compound **241** and epoxide **242**. The reaction between a Grignard reagent (derived from **241**) and **242**, catalyzed by CuI, furnished alcohol **243** with an impressive yield (91%). The subsequent oxidation of **243** resulted in ketone **244** in remarkable yield (92%). Finally, Wittig olefination of **244** led to the formation of alkene **245** in outstanding yield (96%).

The efficient cyclopropanation of **245** using dimethyl diazomalonate under the catalysis of Rh_2_(esp)^2^ (esp = α,α,α′,α′-tetramethyl-1,3-benzenedipropanoate) resulted in the formation of cyclopropane 1,1-diester **246** as a single diastereomer in remarkable yield (96%). Subsequently, aldehyde **248**, the precursor of [3+2] intramolecular cross-cycloaddition (IMCC), was obtained by subjecting **246** to a Wohl–Ziegler reaction followed by oxidization using *N*-methylmorpholine-*N*-oxide (NMO) [78]. After a comprehensive assessment, AIBN was selected as the radical initiator for the bromination of the methyl group neighboring the benzyl moiety. The yield of benzylbromide **247** was 78%.

Aldehyde **248** was obtained through the subsequent oxidation of **247** using NMO without additional purification and then used directly in the subsequent [3+2] IMCC reaction. Specifically, **248** was reacted with BnNH_2_ in a single-step synthesis to yield imine **249** in the same reaction mixture. Subsequently, under the catalytic action of scandium(III) triflate (Sc(OTf)_3_), **249** underwent intramolecular cyclization ([3+2] cycloaddition) to form tetracyclic compound **250** [79,80]. Thus, **250** was synthesized from benzylbromide **247** as the sole diastereomer with a satisfactory three-step conversion efficiency of 76%. The structure of **250** was verified using X-ray crystallography. Its stereochemical configuration was similar to that of calyciphyllines D and F and caldaphnidine M.

The Krapcho decarboxylation of compound **250** yielded monoester **251** in a diastereomeric mixture with an approximately equimolar ratio. Monoester **251** underwent hydrolysis to yield acid **252**. Following this, a modified procedure was employed for the Barton decarboxylation of acid **252**, utilizing **253** and *tert*-butylthiol (*t*-BuSH) as the hydrogen donor to produce **254**. After multiple attempts, Newman’s method (Na_2_S/*N*-methylpyrrolidone (NMP)) [81] was ultimately found to achieve the synthesis of **255** in impressive yield (86%). The oxidative dearomatization of **255** using PIDA [82,83,84] afforded **256** in 72% yield. The structure of **256** was confirmed by X-ray crystallography. Finally, **257** was obtained with high selectivity and a high yield of 96% by hydrogenating **256**. Based on this successful synthesis, the researchers turned to the complete synthesis of calyciphyllines D and F and caldaphnidine M by a dual Michael addition approach.

### 3.3. Research on the Synthesis of Yuzurimine-Type Alkaloids

#### 3.3.1. Synthesis of Heterocyclic Segments of Deoxyyuzurimine and Macrodaphnine

**Scheme 16:**
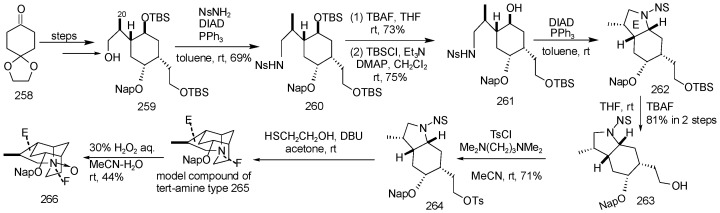
Synthesis of the heterocyclic segments of deoxyyuzurimine and macrodaphnine.

In 2019, Sakakura’s group reported the synthesis of the heterocyclic components found in the yuzurimine-type alkaloids deoxyyuzurimine and macrodaphnine [85]. As depicted in Scheme 16, the synthesis involved a challenging multistep reaction from known compound **258** to the intricate intermediate **259** [86]. The utilization of alcohol **259** in the Mitsunobu reaction [87] resulted in the formation of nitrobenzenesulfonamide **260**, which was later transformed into **261** as a precursor for an intramolecular Mitsunobu reaction. This effectively facilitated the synthesis of the E-ring segment, resulting in the formation of bicyclic compound **262** with minimal impurities. After the removal of the TBS group from **262** using TBAF, the impurities were isolated to acquire alcohol **263** as the sole isomeric form. Subsequent tosylation of the hydroxy group of **263** was achieved using Tanabe’s method, yielding tosylate **264**. Finally, the *tert*-amine model compound, **265**, was synthesized through the sequential elimination of the nitrobenzenesulfonyl (Ns) group and intramolecular S_N_2 reaction [88]. Additionally, *N*-oxide **266** was synthesized from **265** using H_2_O_2_.

#### 3.3.2. Synthesis of AE Bicyclic Structure of Yuzurine

**Scheme 17:**
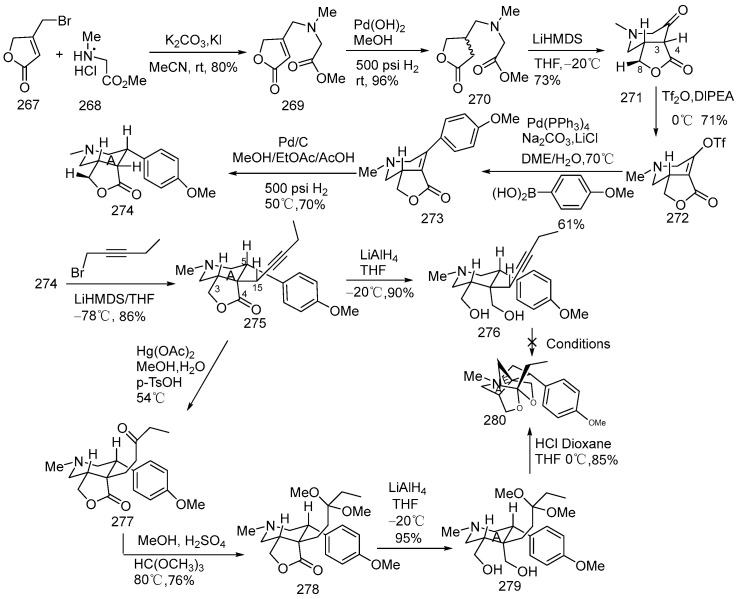
Synthesis of the AE bicyclic structure of yuzurine.

Yang’s group developed a succinct method for synthesizing the AE bicyclic system of the yuzurimine-type alkaloid yuzurine in 2017 [89]. As depicted in Scheme 17, the synthesis employed commercially accessible starting materials, 4-(bromomethyl)-5-hydrofuran-2-one (**267**) and sarcosine methyl ester hydrochloride (**268**), to produce γ-butyrolactone **269** in impressive yield (80%) through *N*-allylation followed by hydrogenation to yield **270**. Next, Dieckmann condensation was achieved by treating **270** with LiHMDS in dry THF at −20 °C, yielding β-keto ester **271.** Enoltriflate **272** was then synthesized in significant yield (71%) by reacting **271** with Tf_2_O in CH_2_Cl_2_ in the presence of DIPEA. Subsequently, **272** was subjected to a Suzuki reaction with 4-methoxyphenyl boronic acid, leading to the formation of intermediate **273**. The piperidine analog **274** was obtained through hydrogenation of the double bond in **273**. The reaction between lactone **274** and LiHMDS in THF at −78 °C generated alkyne **275** while effectively forming the quaternary carbon center C4. The lactone in **275** was then reduced using LiAlH_4_ in dry THF at a temperature of −20 °C, resulting in the formation of diol **276**, which can be utilized for subsequent oxyfunctionalization reactions.

Despite attempts to transform **276** using De Brabander’s method [90] involving [Cl_2_Pt(CH_2_CH_2_)]_2_, MeAuPPh_3_/AgPF_6_, PdCl_2_(PhCN)_2_, and PdCl_2_(MeCN)_2_, a complex mixture was produced without the desired ketal product. Moreover, upon exposure to HgCl_2_/Et_3_N in CH_2_Cl_2_, following a previously reported procedure [91], an assortment of undisclosed compounds was detected. Thus, the researchers reacted **275** with mercury(II) acetate (Hg(OAc)_2_) and *p*-TsOH in a mixture of MeOH and H_2_O at 54 °C, leading to the formation of ketone **277** in 78% yield. Moreover, no undesired isomers formed. Subsequently, **277** was converted to dimethyl ketal **278**. Compound **278** was further reduced with LiAlH_4_ to obtain dihydroxyl compound **279**. Notably, the reaction of **279** with HCl/dioxane in THF at 0 °C ultimately formed the AE bicyclic intermediate **280** in 85% yield.

### 3.4. Research on the Synthesis of Daphnicyclidin-like Alkaloids

#### 3.4.1. Synthesis of ACE Tricyclic Structures Resembling Those of Daphnicyclidin A and Dehydroxymacropodumine A

**Scheme 18:**
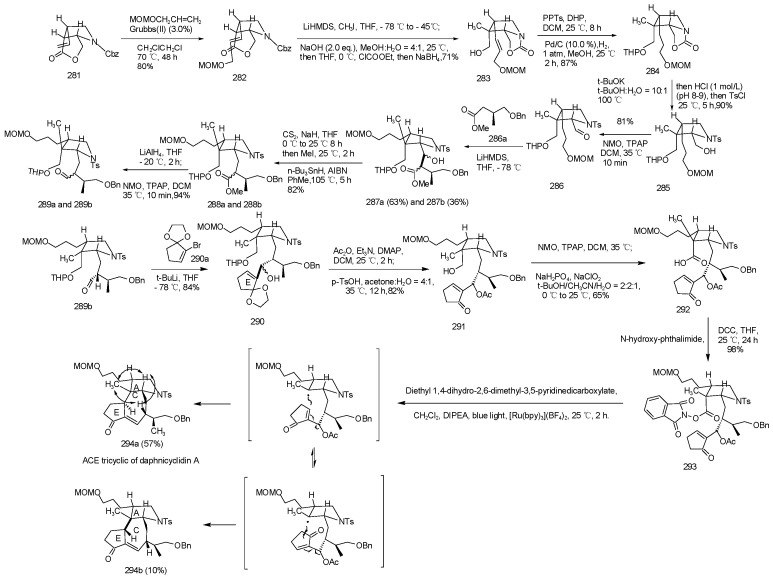
Synthesis of ACE tricyclic structures similar to those of daphnicyclidin A and dehydroxymacropodumine A.

In 2020, Yang’s group reported a method for the production of ACE tricyclic systems resembling those of daphnicyclidin-type alkaloids daphnicyclidin A and dehydroxymacropodumine A [92]. As depicted in Scheme 18, the reaction of **281** with an MOM-protected allyl alcohol in the presence of Grubbs II catalyst afforded **282** in impressive yield (80%). Compound **282** was further processed using THF as the solvent along with LiHMDS and methyl iodide (MeI) at temperatures ranging from −78 to −45 °C, followed by hydrolysis with NaOH in a mixture of MeOH and H_2_O, removal of the MeOH/H_2_O solvent, addition of THF to the residue, and addition of ethyl chloromethylate. This was followed by a reduction in NaBH_4_ to afford alcohol **283**. Alcohol **283** was protected using THP, followed by a reduction of the double bond to obtain **284**. The oxazolinone was hydrolyzed using *t*-BuOK in an aqueous solution of *t*-BuOH at 100 °C, then the pH was adjusted to 8–9, and *p*-toluenesulfonyl chloride (*p*-TsCl) was added to obtain **285**. Subsequent DMP oxidation of the primary alcohol afforded aldehyde **286**. Aldehyde **286** was introduced during the treatment of ester **286a** with LiHMDS, which provided secondary alcohols **287a** and **287b** in yields of 63% and 36%, respectively. The hydroxyl groups at positions **287a** and **287b** were eliminated through Barton deoxygenation, resulting in the formation of **288a** and **288b** in impressive yield (82%). Subsequently, the ester moieties in **288a** and **288b** were converted to alcohols using LiAlH_4_ in THF at −20 °C. The resulting alcohols were then oxidized utilizing NMO/tetrapropylammonium perruthenate (TPAP) to afford diastereomeric aldehydes **289a** and **289b**.

Product **290** was obtained by reacting **289b** with **290a** in THF at −78 °C after treatment with *t*-BuLi. An acetyl group was introduced to protect the new secondary alcohol, after which the ethylene glycol and THP protecting groups were removed to obtain primary alcohol **291** in high yield (82%). The hydroxyl group was rearranged using NMO/TPAP to produce an aldehyde, followed by oxidation with NaH_2_PO_4_/NaClO_2_ to yield **292**. Subsequently, decarboxylation radical conjugate addition was performed on carboxylic acid **292** using MacMillan’s conditions [93]. The carboxyl group was then converted using Overman’s conditions [94] to produce **293**. The synthesis of **294a** and **294b** was achieved under blue light irradiation in the presence of tris(2,2′-bipyridyl)ruthenium tetrafluoroborate ([Ru(bpy)_3_](BF_4_)_2_), diethyl 1,4-dihydro-2,6-dimethyl-3,5-pyridinedicarboxylate, DIPEA, and anaerobic DCM.

#### 3.4.2. Formation of ABCE Ring Substructure of Daphnicyclidin A

**Scheme 19:**
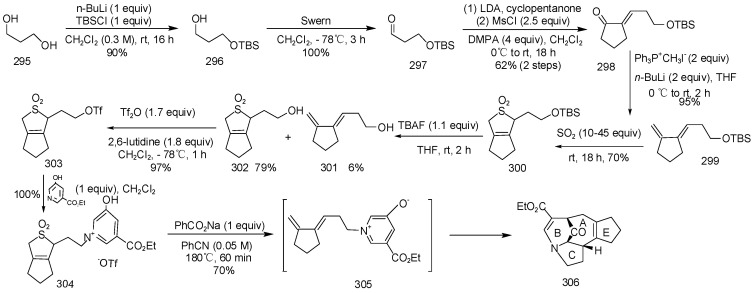
Formation of the ABCE ring substructure of daphnicyclidin A.

Harmata and colleagues devised a method for intramolecular [4+3] cycloadditions involving oxidopyridinium ions, leading to the formation of the ABCE ring substructure of daphnicyclidin A [95,96]. As depicted in Scheme 19, the synthesis began by directly converting propane-1,3-diol **295** into alcohol **296** with an impressive yield (90%) using a monoTBS protection strategy. Subsequently, employing an established protocol [97,98], **296** was quantitatively converted to aldehyde **297** by Swern oxidation with high efficiency. Next, aldol condensation between **297** and cyclopentanone followed by elimination produced enone **298** in 62% total yield. Finally, diene **299** was obtained through Wittig olefination in exceptional yield (95%). Diene **299** was protected by treatment with SO_2_(liq, neat) [99], resulting in the formation of sulfone **300** in 70% yield. Subsequently, the TBS protecting unit of **300** was eliminated to afford alcohol **302** and a small amount of byproduct **301**. Triflate derivative **303** was obtained from **302** with an impressive yield (97%). The interaction between triflate **303** and ethyl 5-hydroxynicotinate led to the complete production of pyridinium salt **304**. Compound **305** was obtained via sulfonation deprotection/intramolecular [4+3] cycloaddition of salt **304**, and then cycloadduct **306,** which possessed the ABCE tetracyclic ring system of daphnicyclidin A, was obtained. Finally, cycloadduct **306**, which possessed the ABCE tetracyclic ring system of daphnicyclidin A, was obtained through the sulfation deprotection/intramolecular [4+3] cycloaddition of salt **304**.

#### 3.4.3. Synthesis of ABC Tricyclic Structure of Daphnicyclidin A

**Scheme 20:**
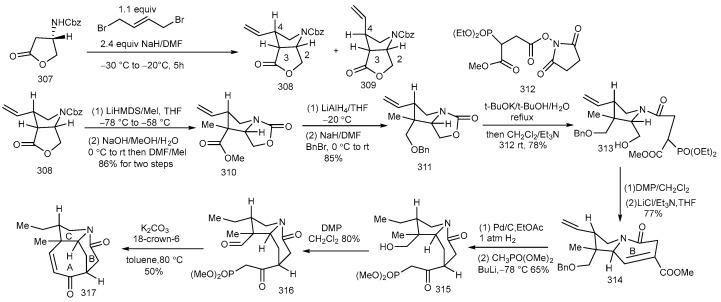
Formation of ABC tricyclic structure of daphnicyclidin A.

The ABC tricyclic system of daphnicyclidin A was synthesized by Yang’s group in 2017 using a substrate-stereocontrolled approach [100]. As depicted in Scheme 20, compound **307** was converted to **308** and **309** via alkylation reactions, and **308** was reacted with LiHMDS and MeI in THF within the temperature range of −78 to −58 °C, followed by a reaction with 2 equiv of MeOH(aq)/NaOH. The solvent was then switched to DMF, followed by the addition of MeI, thus forming oxazolinone **310** in 86% overall yield from **308**. Compound **310** was then reduced, and a benzyl group was added to obtain **311**. Compound **311** was treated with *t*-BuOK at 100 °C overnight and then reacted with phosphate ester **312** to produce amine **313** in 78% yield. An aldehyde was used as a reactant for molecular HWE following the Dess–Martin oxidation of **313**. The formation of the B ring under Rathke’s conditions led to the synthesis of **314** in 77% yield. Following the hydrogenolysis of **314** using 10% Pd/C in the presence of H_2_ gas (pressure: 1 atm), the obtained ester was reacted with the lithium salt of dimethyl methylphosphonate in THF at −78 °C, affording β-ketophosphonate **315** in 65% yield. The conversion of alcohol **315** to aldehyde **316** was achieved by Dess–Martin oxidation. Subsequently, the resulting compound was handled with K_2_CO_3_/18-crown-6 in toluene and stirred at 80 °C for an extended period. This resulted in the formation of the A ring in **317**. Notably, the arrangement of stereocenters in **317** was confirmed to align with that in (+)-daphnicyclidin A.

#### 3.4.4. Construction of 5/6/7 Tricyclic Core of Daphnicyclidin-Type Alkaloids

**Scheme 21:**
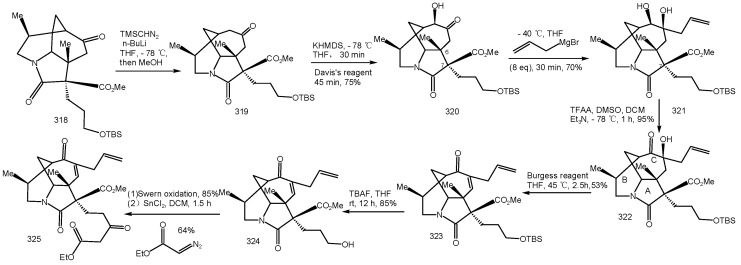
Construction of the 5/6/7 tricyclic core of daphnicyclidin-type compounds.

A rapid synthesis for the 5/6/7 tricyclic core found in daphnicyclidin-type alkaloids was reported by She’s group [101]. As depicted in Scheme 21, they subjected tricyclic ketone **318** to base-mediated ring dilation rearrangement in the presence of trimethylsilyl diazomethane (TMSCHN_2_) to afford 5/6/7 tricyclic ketone **319**. Notably, **319** was the sole product resulting from this ring expansion and migration [102]. Reacting **319** in the presence of KHMDS and Davis’s reagent led to the synthesis of α-hydroxy ketone **320** in high yield (75%) with exclusive diastereoselectivity [103]. The treatment of **320** with excess allylMgBr triggered a 1,2-addition reaction to form tertiary alcohol **321**. Subsequently, the vicinal diol **321** was cleaved using TPAP/NMO, IBX, and DMP as oxidizing agents to afford a ring-opened product. After extensive experimentation, it was determined that Swern oxidation provided optimal results for this transformation. When (COCl)_2_ was employed as a reagent, the desired product (**322**) only obtained a modest yield (24%), whereas the yield increased to an impressive 95% when using trifluoroacetic anhydride (TFAA) [102]. The α-hydroxyketone **322** was treated with the Burgess reagent, leading to the synthesis of dehydrated product **323** in satisfactory yield. Diene **323** was subsequently reacted with TBAF, resulting in product **324** after desilylation. Finally, an aldehyde compound was synthesized using Swern oxidation conditions, followed by a Roskamp reaction to yield β-ketoester **325** [102].

### 3.5. Synthetic Studies Toward Other DAs

#### 3.5.1. Synthesis of ABC Tricyclic Moiety of Calyciphylline N

**Scheme 22:**
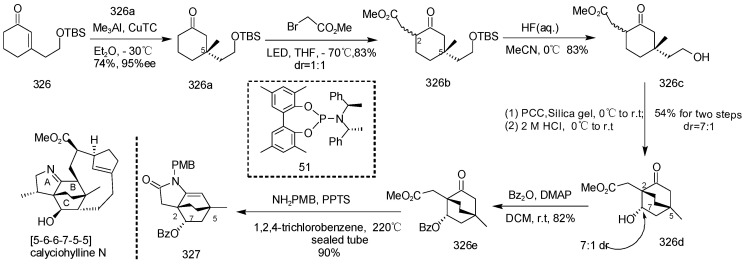
Synthesis of the ABC tricyclic moiety found in calyciphylline N.

Tang’s group recorded the production of a tricyclic compound with a 5/6/6 ABC arrangement resembling that of daphmanidin A-type alkaloid calyciphylline N [64]. This approach, as depicted in Scheme 22, involved a seven-step sequence starting from the known unsaturated ketone **326**. The asymmetric addition of **326** and trimethylaluminum was performed under the action of a copper catalyst with **51** as ligand [25], resulting in an enantioenriched ketone intermediate **326a** in satisfactory yield (74%) with exceptional enantioselectivity of up to 95% ee. Intermediate **326b** was subsequently treated with methyl 2-bromoacetate in THF at −78 °C to afford **326c**. The product contained two diastereomers in equal proportions. After removing the TBS group of **326c**, it was converted into a bicyclo[2.2.2]octanone BC core in **326d** by oxidizing the resulting alcohol with pyridinium chlorochromate (PCC) and inducing an intramolecular aldol reaction with acid mediation. This process achieved high stereoselectivity at C7 (diastereomeric ratio (dr) = 7:1) in just two steps with a 54% yield. To obtain the target bicyclic intermediate **326d**, an intramolecular aldol-type cyclization with high *endo* selectivity was performed. The secondary alcohol in **326d** was protected to give **326e** and subjected to thermal condensation in the presence of 4-methoxyphenylmethanamine (PMBNH_2_) and pyridinium *p*-toluenesulfonate (PPTS), resulting in ABC tricycle **327** in high yield. Notably, the ABC tricyclic framework of **327** comprised the bicyclo[2.2.2]octanone BC core and three quaternary stereocenters of calyciphylline N. It may go through processes such as ring-closing metathesis (RCM) reaction [104] from the ABC three-ring system to get calyciphylline N.

#### 3.5.2. Synthesis of ABCF Tetracyclic Structure of Calyciphylline N

**Scheme 23:**
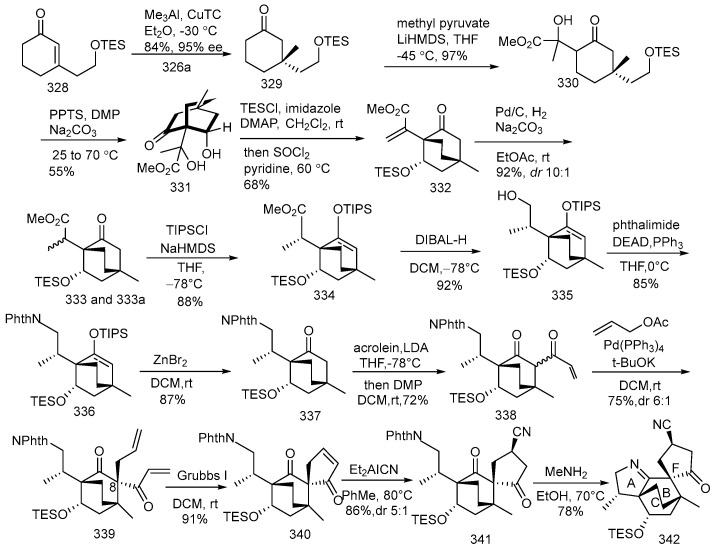
Synthesis of the ABCF tetracyclic structure of calyciphylline N.

The ABCF tetracyclic framework of calyciphylline N was constructed by Qin’s group in 2018 [105]. As shown in Scheme 23, the triethylsilyl ether (TES)-protected substrate **328** was efficiently transformed to adduct **329**. Subsequently, **329** underwent an intermolecular aldol reaction using methyl pyruvate [106] and LiHMDS in THF at −45 °C, thus forming **330**. Bicyclic compound **331** was synthesized with DMP instead of PCC by utilizing Na_2_CO_3_. The secondary alcohol in **331** was selectively protected through treatment with TESCl/imidazole/DMAP. Silyl ether **332**, obtained without prior purification, was subjected to elimination conditions in heated pyridine using SOCl_2_. This led to the formation of α,β-unsaturated ester **332**. After subjecting **332** to catalytic hydrogenation using Pd/C in the presence of Na_2_CO_3_, two distinct esters, **333** and **333a**, formed in EtOAc in 92% total yield (dr = 10:1). Compound **333** was treated with TIPSCl/NaHMDS to afford silyl enol ether **334** in excellent yield (88%). The subsequent conversion of **334** to primary alcohol **335** was accomplished using diisobutylaluminium hydride (DIBAL-H) in high yield (92%). Encouragingly, **335** was reacted with phthalimide under Mitsunobu conditions to obtain product **336** [107]. Selective desilylation employing ZnBr_2_ [108] successfully released the desired ketone **337**.

Then, the researchers synthesized the C8 center via aldol condensation between **337** and acrolein. Diketone **338** was obtained in satisfactory yield (72%) via DMP oxidation. Next, Tsuji–Trost allylation [109] was employed to introduce an allyl group, resulting in diene **339** as the predominant stereoisomer (dr = 6:1). In the subsequent steps, Grubbs first-generation catalyst [110] was employed for the metathesis of **339**, resulting in cyclopentenone motif **340** with a high efficiency of 91%. Thereafter, Nagata conjugate cyanation [111] was utilized to introduce a CN group onto enone **340** as a surrogate for the CO_2_Me group in the target molecule. Fortunately, the 1,4-hydrocyanation of **340** using Nagata’s reagent (Et_2_AlCN) [111] proceeded smoothly in heated toluene. This formed tricyclic compound **341**, which featured the functionalized F ring, in high yield. Subsequently, heating **341** to 70 °C in methylamine (MeNH_2_) and ethanol (EtOH) [112] efficiently removed the phthalimide group and spontaneously formed an imine group, leading to ring closure and thus the formation of the ABCF tetracyclic framework (**342**).

#### 3.5.3. Construction of AC Ring Moiety of Daphnilactone B-Type Alkaloids

**Scheme 24:**
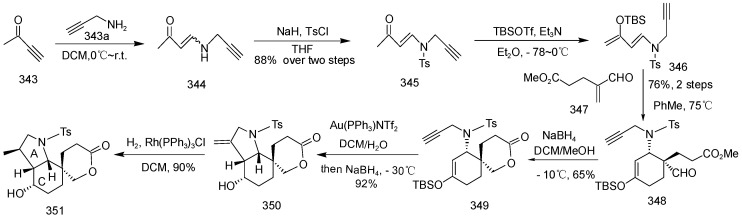
Construction of the AC ring moiety of daphnilactone B.

In 2019, J. Xu’s group synthesized the AC ring moiety of daphnilactone B-type alkaloids in 30% overall yield through a seven-step procedure involving an exceptionally efficient Diels–Alder reaction and an Au-catalyzed Conia-ene reaction [113]. As shown in Scheme 24, acetylenone (**343**) and acetylenamine (**343a**) underwent the Michael addition to produce **344**. Compound **344** was then reacted with NaH and *p*-TsCl to obtain **345** after removing the H from the N atom in **344**. Under the action of Et_3_N, the enol hydroxyl group of **345** was protected using TBS to obtain enol silyl ether **346**, which then underwent a Diels–Alder reaction with aldehyde **347** to obtain compound **348** with a six-cell ring. Subsequently, the aldehyde hydroxyl group of **348** was reduced, followed by intramolecular cyclization to obtain **349**.

Next, the researchers attempted to use substance **349** to construct the A ring of the target compound by an Au-catalyzed Conia-ene reaction [114]. After a series of screening experiments, they used 10 mol% triphenylphosphonogold(I) bis(trifluoromethanesulfonyl)imide salt (Au(PPh_3_)NTf_2_) in DCM and H_2_O (10:1 *v*/*v*). Under these conditions, cyclization occurred quickly at room temperature; however, the cyclization product was not stable. The product was then directly reduced to stable compound **350** using NaBH_4_. Finally, **350** was reacted in DCM in the presence of Wilkinson’s catalyst (chlorido-tris(triphenylphosphine)rhodium(I) (Rh(PPh_3_)_3_Cl)) under a H_2_ atmosphere to produce **351** [115].

#### 3.5.4. Synthesis of ABCE Tetracyclic Framework of Daphenylline

**Scheme 25:**
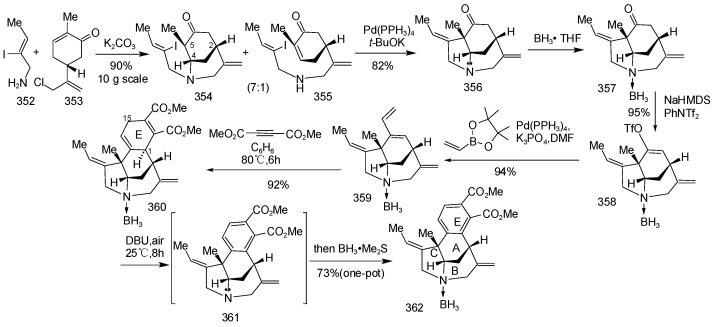
Efficient synthesis of the ABCE tetracyclic framework of daphenylline.

Synthesis of the ABCE tetracyclic framework of daphenylline has also been achieved [116]. The synthesis, as shown in Scheme 25, began with a cascade *N*-alkylation/aza-Michael addition reaction between chloride **353** and amine **352** to produce the bridged aza [3.3.1]bicycle **354** [102,117]. The reaction proceeded smoothly and generated **354** as well as retro aza-Michael addition product **355** in a 7:1 ratio. Subsequent Pd-catalyzed enolate α-vinylation afforded bowl-shaped tricyclic tertiary amine core **356**. Introducing a THF/BH_3_ complex to **356** led to the formation of the borane-complexed aza-tricyclicketone **357**. Subsequently, **357** underwent triflation using NaHMDS and *N*-phenyl bis(trifluoromethanesulfonimide) (PhNTf_2_), affording **358** an almost complete yield [118]. Suzuki coupling between **358** and vinyl borate proceeded smoothly, leading to the synthesis of amine-borane diene **359** in high yield (94%). Dimethyl acetylenedicarboxylate was used as a diene body to react with **359** to produce the desired cyclohexadiene intermediate **360**. The subsequent aromatization step [119] involved the isolation of aromatic tertiary amine **361** in ambient air. To simplify the purification process, BH_3_·Me_2_S was introduced in situ to form amine-borane complex **362**. Notably, **362** exhibits structural similarity to the ABCE tetracyclic framework of (+)-daphenylline.

#### 3.5.5. Production of Central Framework of Daphnimacropodine

**Scheme 26:**
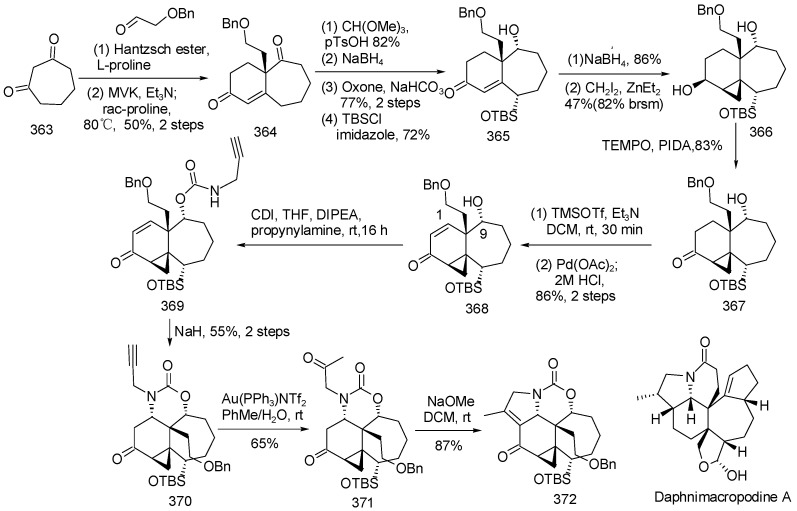
Production of the central framework of daphnimacropodine.

In 2019, J. Xu’s team reported the synthesis of the central framework of daphnimacropodine [120]. The synthesis, as shown in Scheme 26, was initiated from the racemic form of Wieland–Miescher-type diketone **364**, which was prepared from 1,3-cycloheptanedione **363** through a two-step process involving reductive alkylation [121] and aldol condensation. The preparation of **365** was achieved by Oxone-mediated γ-oxidation [122] and protection of the secondary hydroxyl group with TBS to avoid steric hindrance. However, attempts to construct the adjacent quaternary centers through conjugate addition using Luche’s alkylzinc conditions [123] were unsuccessful. Instead, the neighboring quaternary centers were synthesized by selectively reducing the enone motif to obtain **366**. Subsequently, **366** was subjected to OH-directed cyclopropanation and TEMPO-mediated oxidation to produce ketone **367**. Enone **368** was then obtained through Saegusa–Ito oxidation and carbamate derivative **369** was synthesized by sequential treatment with carbonyldiimidazole (CDI) and propynylamine. Carbamate **369** was transformed into the crucial intermediate **370** via NaH-promoted intramolecular Michael addition. Notably, the structure of **370** was unequivocally confirmed by single-crystal X-ray diffraction. Intermediate **370** was then subjected to Au-catalyzed hydration, leading to the formation of intermediate **371**. Finally, intermediate **371** underwent aldol condensation in the presence of sodium methoxide (NaOMe) to yield **372** with the intended hydropyrrole moiety.

#### 3.5.6. Synthetic Studies Toward Longeracemine

**Scheme 27:**
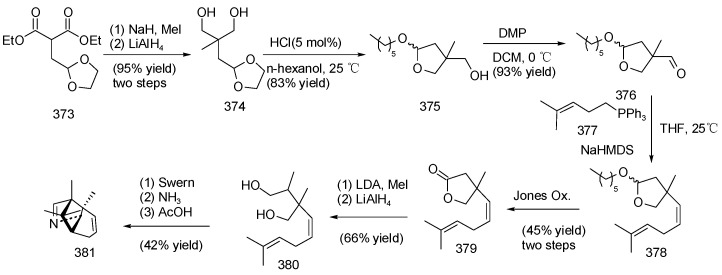
Synthetic studies toward longeracemine.

In 2018, Cox and Wood reported a synthetic method for the azabicyclic core of longeracemine [124]. As depicted in Scheme 27, the well-known diethylmalonate **373** underwent alkylation followed by reduction using LiAlH_4_ to produce diol **374**. Upon exposure to catalytic acid in *n*-hexanol, **374** reacted to form **375** with an equal mixture of diastereomeric acetals. The incorporation of *n*-hexanol increased the molecular weight of **375** and the subsequent oxidation product (**376**). The exposure of neopentyl aldehyde **376** to the homoprenyl phosphonium ylide **377** resulted in the formation of cis olefin **378** as the sole diastereomer. Subsequently, acetal **378** was converted to lactone **379** through a one-pot Jones oxidation, followed by sequential methylation and reduction to yield diol **380** as the sole diastereomer. Notably, diol **380** exhibited excellent substrate reactivity toward cascade cyclization and efficiently produced 2-azabicyclo[2.2.1]heptane **381** under conventional reaction conditions.

#### 3.5.7. Development of ACE Tricyclic Structure of Dehydroxymacropodumine A

**Scheme 28:**
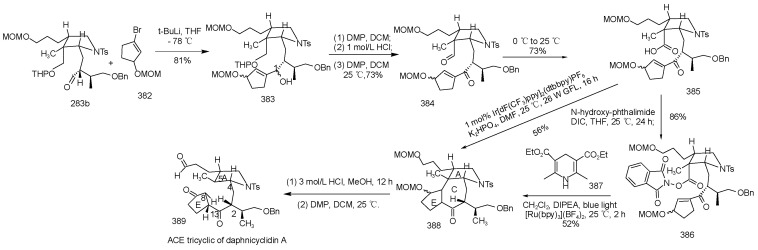
Development of ACE tricyclic structure of dehydroxymacropodumine A.

The ACE tricyclic structure of dehydroxymacropodumine A has also been synthesized [92]. As demonstrated in Scheme 28, **382** was reacted with *t*-BuLi in THF at −78 °C, followed by the addition of **283b** for the attachment of a cyclopentene moiety to afford secondary alcohol **383**. Sequential DMP oxidation of **383**, removal of the THP protecting group, and oxidation of the resulting primary alcohol produced aldehyde **384** in 73% yield. Compound **384** continued to oxidize into compound **385**. Reactive compound **385** was further subjected to light irradiation under MacMillan’s photocatalyst conditions (1 mol% Ir-based catalyst (Ir[dF(CF_3_)ppy]_2_(dtbbpy)PF_6_) in deoxygenated DMF), resulting in the formation of **388** through conjugate addition. Irradiation with a common household fluorescent bulb for approximately 16 h led to a satisfactory yield of 56%. The researchers further examined Overman’s state by transforming carboxylic acid **385** into *N*-hydroxyphthalimide ester **386**, using the same condition as that of **293** to **294a**, resulting in the production of **388** in 52% yield. To further elucidate its structure, the MOM groups of **338** were removed, resulting in the conversion of the hydroxyl groups to carbonyl ones and yielding compound **389**, which resembled the ACE tricyclic structure of dehydroxymacropodumine A.

## 4. Total Syntheses of DAs

In the last few years, several synthetic chemists have reported the total synthesis of various DAs, building on the work discussed in Section 3. This section highlights some of the intricate strategies that have been developed to recreate these complex natural products.

### 4.1. Total Synthesis of (−)-Daphenylline and (−)-Himalensine A by Qiu’s Group

**Scheme 29:**
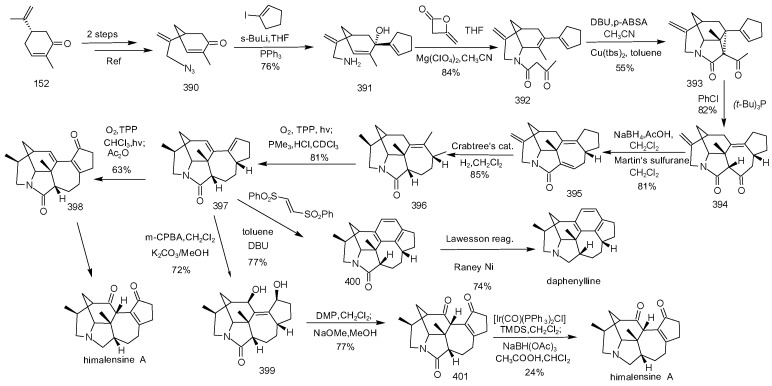
Total synthesis of (−)-Daphenylline and (−)-Himalensine A by Qiu’s group.

Daphenylline and Himalensine A were reported by Hao [125] and Yue group [126] separately. In 2021, Qiu’s group reported the complete synthesis of (−)-daphenylline and (−)-himalensine [127]. As depicted in Scheme 29, the synthesis commenced with (*S*)-carvone (**152**) to produce allyl azide **390** via a two-step procedure [128]. Primary amine **391** was then obtained in 76% yield via 1,2-addition between cyclopenenyl lithium (derived from the lithiation of 1-iodocyclopent-1-ene using *sec*-butyllithium (*s*-BuLi)) and azide **390**. Subsequently, Staudinger reduction was conducted using PPh_3_, followed by acylation of the amino group with diketene and an Mg(ClO_4_)_2_ catalyst to synthesize amide atropisomer **392** by intramolecular amidocyclization. By employing 4-acetamidobenzenesulfonyl azide (*p*-ABSA) and DBU, diazo acetoacetamide was gradually introduced into a toluene solution containing 10 mol% copper(II) *tert*-butylacetoacetate (Cu(tbs)_2_), resulting in the formation of cyclopropyllactone **393**. The treatment of **393** with tri-*tert*-butylphosphine (P(*t*-Bu)_3_) in chlorobenzene [129] at 110 °C resulted in formal rearrangement to afford cycloheptenone **394**. After the reduction of **394** with NaBH_4_, dehydration using Martin’s sulfurane resulted in α,β-unsaturated amide **395** in 81% yield. Simultaneous regio- and diastereoselective hydrogenation of **395** employing Crabtree’s catalyst afforded diastereomerically pure **396**. Using the Schenck ene reaction with TPP as a photosensitizer, the adjacent position of **396** was oxidized by singlet oxygen, followed by the addition of trimethylphosphine (PMe_3_) and ethyl acetate to generate diene **397**. Motivated by a valuable precedent [130], the researchers reacted **397** with *trans*-1,2-bis(phenylsulfonyl)ethylene in toluene at 150 °C to obtain **400**. They then added DBU to obtain (−)-daphenylline in 74% yield.

A subsequent investigation involved the direct conversion of diene **397** into (−)-himalensine A via a hetero [4+2] reaction with singlet oxygen, followed by Kornblum–DeLaMare rearrangement [131] of the resulting endoperoxide. Surprisingly, the TPP-sensitized Schenck ene photooxygenation of **397** resulted in the formation of an intriguing hydroperoxide. The hydroperoxide was then treated with Ac_2_O in situ to obtain intermediate **398** in 63% yield. Notably, **398** was further transformed into (−)-himalensine A through a two-step process.

An alternative method was also developed to obtain (−)-himalensine A. The selective epoxidation of **397** with *m*-chloroperoxybenzoic acid (*m*-CPBA), followed by ring opening using the *m*-chlorobenzoic acid that formed in situ and removal of the benzoyl group with K_2_CO_3_ in MeOH, resulted in diol **399** in 72% yield. DMP oxidation of diol **399**, followed by C=C double bond isomerization using NaOMe, resulted in the synthesis of **401**. Finally, chemoselective reduction with an Ir-based catalyst reduced the lactam carbonyl group of **401** to afford (−)-himalensine A.

### 4.2. Synthesis of (−)-Daphnilongeranin B and (−)-Daphenylline by Zhai’s Group

**Scheme 30:**
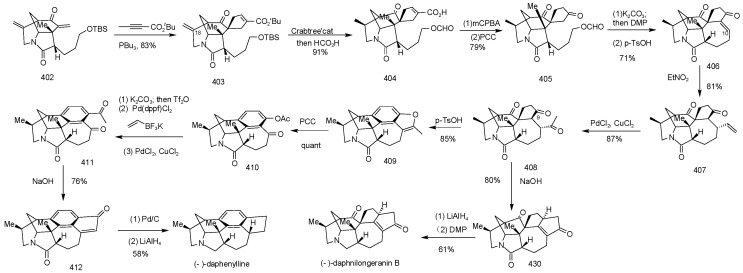
Synthesis of (−)-Daphnilongeranin B and (−)-Daphenylline by Zhai’s group.

The total synthesis of (−)-daphnilongeranin B and (−)-daphenylline was achieved by Zhai’s group in 2018 [132]. The developed cycloaddition was a modification of Lu’s protocol [133]. As shown in Scheme 30, **402** was treated with *tert*-butyl 2-butynoate under the catalysis of K_2_CO_3_/MeOH to afford **403**. Subsequent hydrogenation employing Crabtree’s catalyst, followed by solvent evaporation and treatment with HCO_2_H, provided **404** in a one-pot fashion in high yield (91%). The required ketone derivative, **405**, was synthesized by exposing **404** to Barton’s reagent, followed by oxidation [134]. Compound **404** was treated sequentially with *m*-CPBA and dicyclohexylcarbodiimide (DCC), followed by PCC oxidation, to yield **405** in good overall yield (79%) [135]. The methanolysis of **405** utilizing catalytic K_2_CO_3_ in MeOH and subsequent DMP oxidation generated an unstable aldehyde product under *p*-TsOH-catalyzed conditions (substrate concentration: 0.005 M), resulting in enone **406** in 71% yield. Upon reacting **406** with nitroethane, crystalline product, **407** was unexpectedly formed. Wacker oxidation of **407** yielded **408** [136] in high yield (87%) [137]. The aldol condensation and isomerization of **408** were accomplished by treating it with NaOH in MeOH/brine (1:1 *v*/*v*), resulting in tricarbonyl **430** [138,139]. Subsequent attempts to achieve a chemoselective reduction of the amide proved exceptionally challenging [140]. However, a sequential method that included overall reduction using LiAlH_4_ and subsequent oxidation with DMP was successful, resulting in the synthesis of (−)-daphnilongeranin B [141].

After successfully synthesizing (−)-daphnilongeranin B, the researchers employed intermediate **406** or its analogues to construct the benzene ring found in (−)-daphenylline [125]. There are only a few documented cases of five-membered rings within cyclopentanones being transformed into benzene rings [142]. Initially, the researchers attempted to use **407** as the reactant, but **408** was found to be more effective. Indeed, by reacting **408** with *p*-TsOH in benzene, benzofuran **409** was produced in 85% yield. Reactive oxidation of the furan ring in **409** using PCC resulted in complete conversion to acetate **410** [143]. Through a three-step sequence involving methanolysis/phenolsulfonation, Suzuki coupling, and Wacker oxidation, ester **410** was converted to **411** in 80% overall yield. The daphenylline core was completed by intramolecular aldol condensation to afford **412** in 76% yield, achieved by treating **411** with NaOH(aq) in MeOH/brine (1:1 *v*/*v*) at 80 °C. (−)-Daphenylline was successfully obtained in 58% yield over two steps by subjecting **412** to catalytic hydrogenation using Pd/C, followed by amide reduction with LiAlH_4_.

### 4.3. Total Synthesis of Daphenylline, Daphnipaxianine A, and Himalenine D by A. Li’s Group

**Scheme 31:**
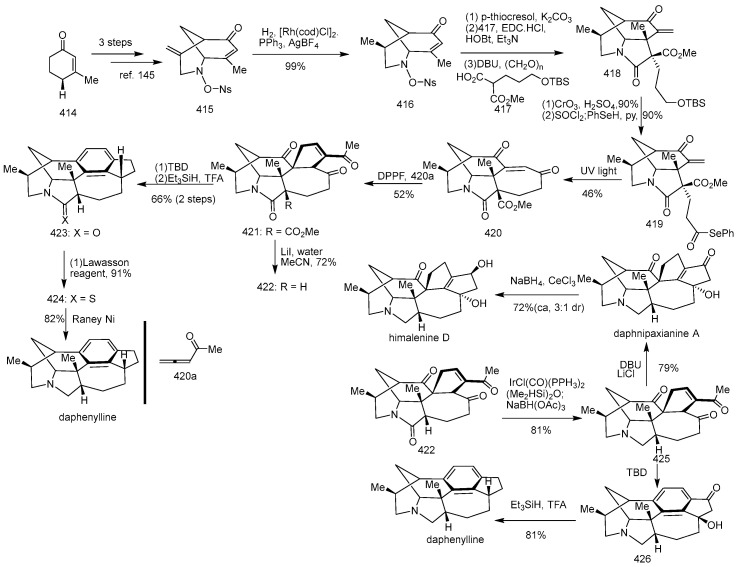
Total synthesis of daphenylline, daphnipaxianine A, and himalenine D by A. Li’s group.

In 2018, A. Li’s group achieved the total synthesis of calyciphylline A-type alkaloids daphenylline, daphnipaxianine A, and himalenine D [144]. As shown in Scheme 31, the synthesis of daphenylline was initiated by constructing enedione **419**. A highly enantioenriched starting material, **414**, was utilized to synthesize bridged bicyclic compound **415** (99% ee) via a well-established three-step process involving alkyne cyclization with an Ag catalyst [145]. A highly selective hydrogenation occurred at the C18=C20 bond of **415** in the presence of a Rh complex (generated in situ from [Rh(cod)Cl]_2_, PPh_3_, and AgBF_4_), resulting in **416** in almost complete yield (99%). Tricyclic enone **418** was then synthesized from **416** by reactions including the sequential removal of the nosyl protecting group, amide formation, intramolecular Michael addition, and aldol condensation. The corresponding carboxylic acid was obtained directly through the Jones oxidation of **418**. Treatment with SOCl_2_ afforded an acyl chloride, which was reacted with benzeneselenol (PhSeH) and pyridine to yield **419**. Subsequently, the UV irradiation of **419** using a Hg lamp provided **420** in 46% yield, presumably through atom-transfer radical cyclization [146] followed by instantaneous β-elimination.

Next, pentacyclic triketone **421** was prepared from **420** through Lu’s [3+2] cycloaddition. The addition of 1,1′-bis(diphenylphosphino)ferrocene (DPPF) and allenyl ketone **420a** likely resulted in the formation of a zwitterionic species; this species then reacted with electron-deficient alkene **420** to afford **421**. The subsequent Krapcho demethoxycarbonylation using LiCl and wet MeCN at 175 °C, along with microwave irradiation, ran into problems but still yielded the desired product (**422**) in 25% yield, along with a small amount (<2% yield) of aromatic compounds. This provided support for the proposed ring expansion/aromatization cascade mechanism. By optimizing the reaction conditions (LiI and MeCN/dimethyl sulfoxide (DMSO) (4:1 *v*/*v*) at 140 °C), the yield of **422** increased significantly to 72%, while a retro-Michael product was also produced at 15% yield. Exposing **422** to triazabicyclodecene (TBD) in toluene at 90 °C provided the desired product in up to 43% yield. However, THF proved to be a better solvent, resulting in a 67% yield. The benzylic carbonyl and hydroxy groups were efficiently reduced using Et_3_SiH/TFA, resulting in the formation of indane **423**. The treatment of **423** with Lawesson’s reagent provided thioamide derivative **424** in high yield (91%). The reduction of **424** with Raney Ni afforded daphenylline with excellent efficiency.

By utilizing modified Nagashima conditions, **422** was successfully transformed into the corresponding enamine. Subsequently, a one-pot reduction with sodium triacetoxyborohydride (NaBH(OAc)_3_) yielded tertiary amine **425**. The treatment of **425** with DBU and LiCl in MeCN at 120 °C afforded daphnipaxianine A a 79% yield. Luche reduction of daphnipaxianine A resulted in acceptable diastereoselectivity at C16 (dr = 3:1), yielding himalenine D with an isolated yield of 72%. Meanwhile, a two-step treatment of **425** led to the formation of daphenylline via intermediate **426**.

### 4.4. Total Synthesis of (−)-Daphenylline by Qiu’s Group

**Scheme 32:**
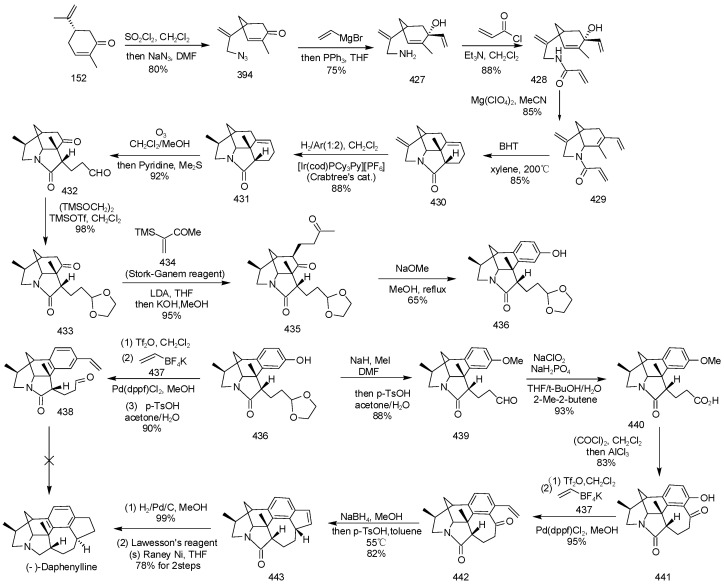
Total synthesis of (−)-daphenylline by Qiu’s group.

Qiu’s group accomplished the complete synthesis of (−)-daphenylline in 2019 [147]. As shown in Scheme 32, they employed Robinson annulation combined with oxidative aromatization to construct the challenging aromatic moiety. The 1,6-dicarbonyl structure was obtained through the ozonolysis of **431**, which was synthesized via consecutive amide cyclization and Diels–Alder cycloaddition. Intermediate **428** was derived from (*S*)-carvone (**152**) via allylic chlorination using SO_2_Cl_2_. The resulting allylic chloride was then directly displaced with sodium azide to yield **394**. A reactive vinyl Grignard reagent was introduced to the ketone carbonyl group of **394**, followed by Staudinger reduction of the azido moiety to produce primary amine **427**. The acylation of **427** with acryloyl chloride and Et_3_N yielded acrylamide **428**. When **428** was treated with a catalytic amount of Mg(ClO_4_)_2_ [147,148] in CH_3_CN [149] under reflux conditions for three h, S_N_1′ amide cyclization occurred, resulting in **429**. Compound **429** was heated at 200 °C for three days in a sealed tube, generating cycloaddition product **430**.

Inspired by the recent work of Dixon’s groups [150], **431** was generated using Crabtree’s catalyst. Ozonolysis of the trisubstituted olefin in **431** afforded ketoaldehyde **432** in high yield (92%). The aldehyde carbonyl group was selectively protected using Noyori’s conditions [151], thus forming intermediate **433** in 98% yield. Notably, **433** was suitable for the Robinson annulation. The Michael addition of **433** and the Stork–Ganem reagent (methyl trimethylsilylvinyl ketone (**434**)) [152] in the presence of LDA afforded an α-silyl ketone. The crude product was treated with KOH in MeOH without purification, leading to cleavage of the TMS moiety and yielding **435**. After numerous attempts [102], phenol **436** was generated by reacting **435** with freshly prepared 1 M NaOMe in MeOH under reflux conditions.

With the aromatic core established, the researchers shifted their attention to constructing the D and F rings through a Friedel–Crafts-type domino cyclization. Aldehyde **438** was obtained in high yield (90%) through a three-step process involving the triflation of phenol **436**, Suzuki coupling with potassium vinyltrifluoroborate (**437**), and subsequent removal of the acetal protecting group. Despite multiple attempts with phenol **436**, it was eventually protected as a methyl ether using MeI/NaH. The resulting compound was then subjected to aqueous acid hydrolysis to obtain aldehyde **439** in 88% yield, which further underwent Pinnick oxidation [153] to yield carboxylic acid **440** in 93% yield. Expanding on the research conducted by Cao’s group [154], the researchers accomplished intramolecular Friedel–Crafts acylation [155] while simultaneously removing the methyl group. Compound **440** was then converted into its corresponding acid chloride and reacted with AlCl_3_ under one-pot conditions to yield intermediate **441**. Inspired by Nazarov’s electrocyclization of divinyl alcohol and the Suzuki coupling conditions reported by Zhai et al. [132], the researchers successfully obtained product **442**. Notably, treating ketone **442** with NaBH_4_ followed by *p*-TsOH in toluene at 55 °C for 20 min resulted in the desired electrocyclization product **443**, similar to Nazarov’s work. Further hydrogenation of the double bond using Pd/C and reduction of the lactam moiety [156] led to a combined yield of 78% for (−)-daphenylline.

### 4.5. Total Synthesis of Himalensine A by Gao’s Group

**Scheme 33:**
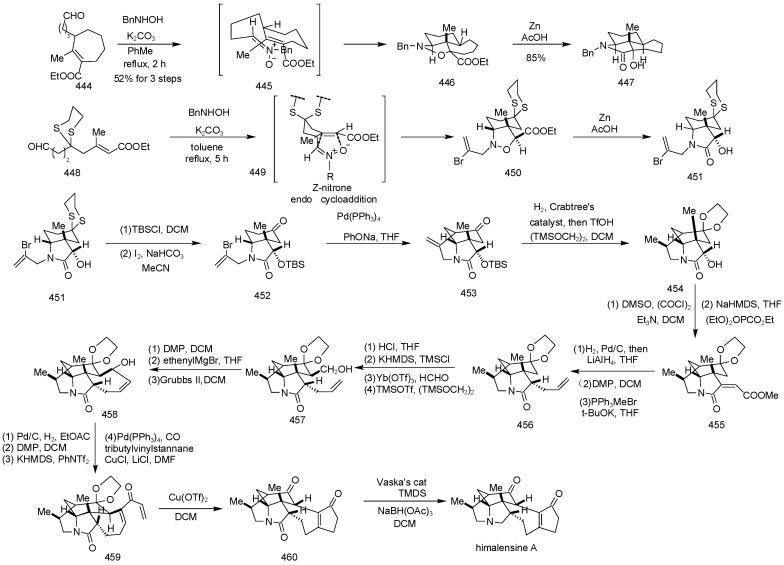
Total Synthesis of Himalensine A by Gao’s group.

In 2019, Gao’s group elucidated a synthetic strategy for the central structure of calyciphylline A-type DAs and ultimately presented a comprehensive total synthesis for himalensine A [157]. As shown in Scheme 33, the reaction between **444** and benzyl hydroxylamine (BnNHOH) led to the formation of intermediate nitrone **445**. Intermediate **445** then underwent a thermodynamic 1,3-dipolar cycloaddition with an electron-deficient alkene, resulting in the formation of cycloadduct **446** as a single diastereomer. The N–O bond in isoxazolidine **446** was reduced and cleaved, leading to the spontaneous formation of tricyclic product **447** through lactamization. The reaction of **448** with various hydroxylamines produced the desired endo-cycloadducts **450**, which efficiently transformed into cis-hydroindoles **451** (A−C rings) with good overall yield. [158]. Compound **451** with TBS ether, followed by the removal of the 1,3-dithiane group, produced ketone **452**, which serves as the precursor for constructing the B ring. Compound **452** was treated with catalytic Pd(PPh_3_)_4_ in the presence of PhONa in THF, resulting in a cyclized product **453** obtained with an impressive yield of 74%. The exocyclic olefin was selectively hydrogenated using Crabtree’s catalyst, followed by one-pot protection of the carbonyl group and deprotection of the TBS group yielded azatricyclic compound **454**. Compound **454** was oxidized to produce aldehyde, which was then reacted with phosphate esters to produce **455**. The stereocontrolled hydrogenation, oxidation state adjustment, and olefination resulted in the formation of **456** with the desired α-configuration, yielding 90% overall. The acetal deprotection of **456** under acidic conditions produced the corresponding ketone, which underwent an aldol reaction to yield the allylic alcohol. After extensive screening of reaction conditions, the silyl enol ether exhibited higher reactivity compared to the active metal enolate. A Yb(OTf)_3_-mediated Mukaiyama aldol reaction with formaldehyde resulted in the formation of **457** containing a hydroxylmethane group, while acrylaldehyde showed no reactivity. The oxidation of **457** with Dess-Martin periodinane, followed by ethenylmagnesium bromide addition and ring-closing metathesis, yielded **458** in an overall yield of 87%, containing the seven-membered D ring. The compound **458** underwent hydrogenation with Pd/C, followed by oxidation to form the corresponding ketone. The ketone was then converted into vinyl triflate under basic conditions. The triflate was carbonylatively coupled with tributylvinylstannane to yield dienone **459** in good yield. This dienone then underwent selective Nazarov cyclization with copper triflate, resulting in the formation of pentacyclic compound **460** in a 74% yield. Compound **460** is an advanced intermediate in the first enantioselective total synthesis of himalensine A. The lactam carbonyl group in **460** was selectively reduced using Vaska’s catalyst [IrCl(CO)(PPh_3_)_2_], enabling the total synthesis of himalensine A.

### 4.6. A Concise Total Synthesis of (−)-Himalensine A by Xu’s Group

**Scheme 34:**
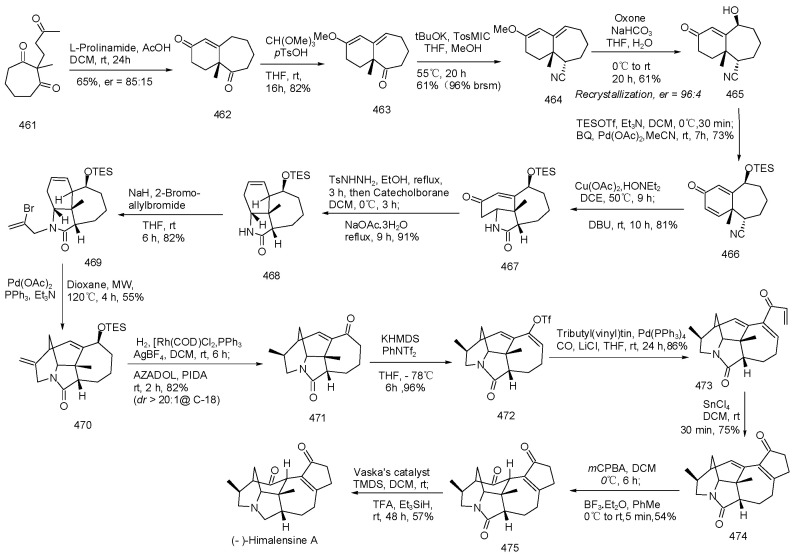
A Concise Total Synthesis of (−)-Himalensine A by Xu’s group.

A concise strategy was described to provide general and diversifiable access to various DAs, which is utilized in the asymmetric synthesis of (−)-himalensine A accomplished in 14 steps by Xu’s group [159]. This approach was initiated from the readily available chiral diketone **462**, which was optimized for enantioselectivity [160]. Importantly, the absolute stereoconfiguration of **462** was corrected, as reported previously [161]. As shown in Scheme 34, the enone motif in **462** was selectively converted into methyl enol ether **463**. The treatment of **463** with toluenesulfonylmethyl isocyanide (TosMIC) resulted in van Leusen homologation [162], yielding nitrile derivative **464** as a single diastereomer. The application of Oxone led to the targeted incorporation of a γ-hydroxyl group on **464**, resulting in the formation of enone **465**. Sequential silylation and Saegusa–Ito oxidation conveniently produced dienone intermediate **466**.

Attempts to achieve the nitrile hydration of **466** using alkali hydroxide, alkoxide, and Rh(I)-mediated methods proved unsuccessful. Promisingly, employing *N*,*N*-diethylhydroxylamine as a promoter for Cu(II)-catalyzed nitrile hydration generated the essential primary amide. This primary amide then underwent intramolecular Michael addition in situ to yield tricyclic γ-lactam **467**. Subsequently, the conversion of enone **467** into alkene migration product **468** was achieved through a one-pot reaction involving hydrazone formation, reduction, and allyldiazenere arrangement using Kabalka’s conditions [163]. Following this, amide nitrogen alkylation led to the formation of Heck reaction precursor **469**, which was treated to produce **470** in 55% yield. The diastereo- and regioselective reduction of the *exo*-alkene in **470** was accomplished using carbonyl group-directed catalytic hydrogenation employing A. Li’s conditions (H_2_, [Rh(cod)Cl]_2_, and AgBF_4_) [164]. Other attempts at hydrogenation, such as by using Crabtree’s [165] or Wilkinson’s catalyst [115], resulted in either undesired diastereoselectivity or no observable reaction.

Next, 2-hydroxy-2-azaadamantane (AZADOL) and PIDA were sequentially added to the reaction mixture, resulting in a one-step oxidation to form enone **471**. Taking inspiration from Shvartsbart and Smith’s impressive synthesis of (−)-calyciphylline N [166], a three-step process was employed to obtain pentacyclic compound **474** from **471** in 62% yield, which involved sequential enol triflate generation, carbonylative Stille coupling, and Nazarov cyclization [166,167]. After other attempts [166,168], **474** was reacted with *m*-CPBA followed by Meinwald rearrangement [169] with BF_3_·Et_2_O in a one-pot reaction to obtain the desired ketone intermediate **475**. The synthesis was finalized by Ir-catalyzed hydrosilylation followed by reduction [170], resulting in the production of (−)-himalensine A. Alternative approaches were explored, including sequential reduction and targeted oxidation of the two secondary hydroxyl groups; however, this resulted in decomposition.

### 4.7. Total Synthesis of Daphenylline by Lu’s Group

To improve the efficiency of the chemical synthesis of daphenylline, Lu’s group adopted a “hide-and-seek” strategy. Specifically, they searched for readily available building blocks that contained hidden structural information relevant to their synthetic target [171]. The Arene building blocks aligned perfectly with this approach [172,173]. Taking inspiration from important studies on β-naphthol dearomatization [173,174,175,176], researchers employed intramolecular oxidative dearomatization using ester-tethered β-naphthol **480** to synthesize daphenylline [174,175].

**Scheme 35:**
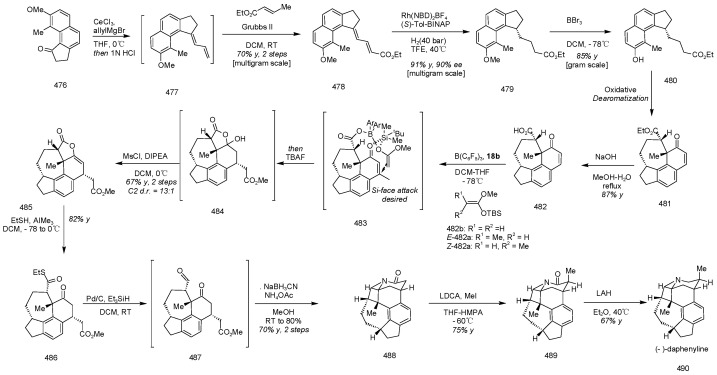
Total Synthesis of Daphenylline by Lu’s group.

As shown in Scheme 35, the synthesis began by obtaining dearomatization precursor **480**. The decagram of indanone **476**, which encompasses the tetrasubstituted arene pattern found in daphenylline, was readily synthesized via a succinct sequence. By condensing it with allylMgBr and subjecting it to cross-metathesis with ethyl crotonate, the researchers efficiently generated a substantial amount of the desired diene **478**. Target product **479** was achieved with exceptional enantioselectivity by employing a chiral Rh catalyst and (*S*)-(−)-2,2′-*p*-tolyl-phosphino)-1,1′-binaphthyl ((*S*)-Tol-BINAP) as a ligand in trifluoroethanol (TFE). Subsequent elimination of the methyl group afforded dearomatization precursor **480**.

Inspired by pioneering studies on β-naphthol dearomatization [176,177], researchers explored the deprotonation-induced oxidative dearomatization of **480** using various potent bases and oxidizing agents. I_2_ [178]—a relatively small oxidant, which was employed by Ma et al. for intramolecular oxidative coupling of indoles [179]—proved the most effective choice. The treatment of **480** with lithiumcyclohexyl isopropyl amide in the presence of I_2_ afforded a highly congested benzo-fused cyclohexenone derivative (**481**) in moderate yield (35%). A diastereomer, *epi*-**481**, was also formed as a minor product in 12% yield. Fortunately, under thermodynamic conditions, *epi*-**481** could be efficiently epimerized to afford **481** in good yield (80%). Consequently, the overall yield of **481** reached approximately 45%.

The researchers then hydrolyzed the ester of **481** to obtain the corresponding acid (**482**). Crude adduct **484** was synthesized via the tris(pentafluorophenyl)borane-catalyzed reaction of **482** and **482b**. Crude adduct **484** was then directly utilized for dehydration to achieve diester **485**. The reaction was hypothesized to occur via active boron enolate ate complex **483** [180], whose silylium group [181] acts as a bridge to bring the reactive centers together, facilitating bonding and thus adduct formation. A brief two-step process involving transthioesterification and Fukuyama reduction of thiolester **486** was utilized to produce **487**. Subsequently, the desired outcome, **488**, was obtained in good overall yield (70%) over two steps. To synthesize (−)-daphenylline, a methyl group was introduced at the C18 position of **488**, followed by simple reduction. It is expected that an identical series of steps could be utilized to acquire the synthetic isomer (−)-daphenylline from the enantiomeric form of **479**.

### 4.8. Total Syntheses of (−)-10-Deoxydaphnipaxianine A, (+)-Daphlongamine E and (+)-Calyciphylline R by J. Xu’s Group

**Scheme 36:**
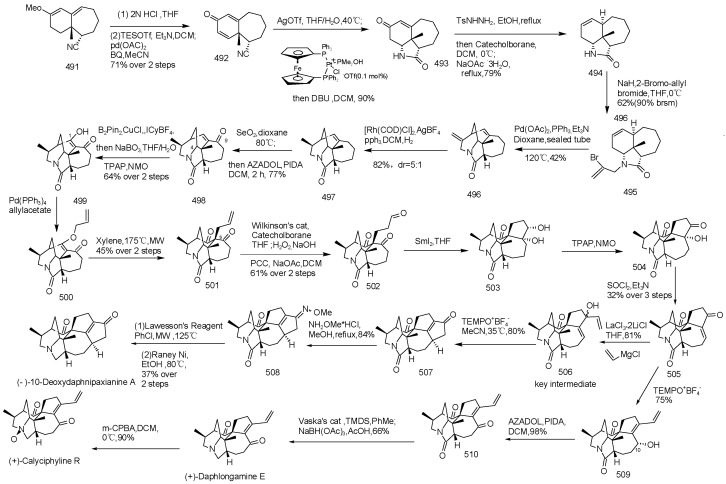
Total Syntheses of (−)-10-Deoxydaphnipaxianine A, (+)-Daphlongamine E, and (+)-Calyciphylline R by J. Xu’s group.

J. Xu’s group synthesized three calyciphylline A-type alkaloids, namely, (−)-10-deoxydaphnipaxianine A, (+)-daphlongamine E, and (+)-calyciphylline R, through late-stage divinyl carbinol rearrangement [182]. As shown in Scheme 36, the synthesis began with chiral nitrile **491**, where the enol methyl ether motif was hydrolyzed and then oxidized by Saegusa–Ito oxidation to form dienone **492**. By utilizing a donor-acceptor Pt catalyst developed by Grubbs et al. [183], the nitrile motif of **492** was efficiently converted into a primary amide through hydration. Subsequent aza-Michael addition involving DBU produced γ-lactam **493**. Further transformations, including Hutchins–Kabalka reductive rearrangement [184], led to the formation of alkene **494**. *N*-Alkylation and intramolecular Heck reaction were employed to construct the critical 2-azabicyclo[3.3.1]nonane moiety within the tetracyclic structure of **496**. Finally, intermediate **497** was obtained at a high dr of 5:1 through diastereoselective hydrogenation of the 1,1-disubstituted alkene of **496** under A. Li’s conditions [144,145,164].

Next, the allylic oxidation of **497** was conducted using SeO_2_ in dioxane at approximately 80 °C, followed by one-step oxidation using PIDA and AZADOL to obtain ketone **498**. Although **498** could be synthesized using previously documented methods, to enhance the strategy and tactical repertoire, conjugate boron addition was employed to achieve the α,β-unsaturated enone motif in **498**. Sequential oxidation resulted in **499**, which exhibited a 1,3-diketone functionality, in 64% yield over two steps.

Tsuji–Trost allylation led to the formation of *C*-alkylated product **500** instead of **501**. To address this issue, an alternative approach involving Claisen rearrangement was utilized to convert enol allyl ether **500** into diketone **501**, which possessed a crucial C8 quaternary center adjacent to the C5 quaternary center. A two-step functionalization involving Rh-catalyzed hydroboration and PCC oxidation afforded aldehyde **502** in 61% yield over two steps. Subsequently, the essential cyclopentane structure was created by SmI_2_-mediated pinacol coupling, which selectively distinguished between the C1 and C9 ketones. The resulting diol, **503**, was then oxidized to form α-hydroxylketone **504**. Elimination of the α-hydroxyl group of **504** under SOCl_2_/pyridine conditions afforded α,β-unsaturated enone **505**. Furthermore, Grignard addition to the enone component of **505** produced significant intermediate **506** (dr ≈ 2:1).

The researchers initially planned to construct the enone moiety of **510** by subjecting **506** to Dauben–Michno rearrangement [185], thereby granting access to (+)-daphlongamine E and (+)-calyciphylline R. However, the use of Iwabuchi’s conditions (TEMPO^+^BF_4_^−^ in MeCN) [186] resulted in unprecedented Nazarov cyclization of the tertiary divinyl carbinol **506** to form **507**. The allyl cation was captured and transformed into intermediate D, which was subsequently oxidized to generate enone **507**. Therefore, the researchers instead focused on converting **507** into (−)-10-deoxydaphnipaxianine A by selectively reducing the amide group. Surprisingly, despite several analogous examples [187], attempts with Vaska’s conditions [188] resulted in an insignificant yield of the desired product. Following these unsuccessful efforts, the researchers protected the C16 ketone in **507** and then combined it with methoxyamine to yield *O*-methyloxime [189], which served as a crucial intermediate for the synthesis of (−)-10-deoxydaphnipaxianine A [190]. Finally, oxime **508** was reacted with Lawesson’s reagent in chlorobenzene, followed by treatment with Raney Ni to afford (−)-10-deoxydaphnipaxianine A.

Subsequently, the researchers investigated the syntheses of (+)-daphlongamine E and (+)-calyciphylline R via Dauben–Michno rearrangement [185] or allylicalcohol rearrangement of **506** [191]. However, the presence of two adjacent rings presented unexpected challenges. Surprisingly, when the researchers varied Iwabuchi’s conditions [186] and used TEMPO^+^BF_4_^−^ in 1,4-dioxane as the reagent, divinyl carbinol **506** was transformed into secondary alcohol **509**. Further oxidation using AZADOL and PIDA converted the C10 hydroxyl group in **509** into enone **510**. Interestingly, **510** exhibited significantly different behavior compared to its analog **507** when applying selective amide reduction conditions with Vaska’s complex (*trans*-chlorocarbonylbis(triphenylphosphine)iridium(I); IrCl(CO)(PPh_3_)_2_),. This was ascribed to its less sterically hindered amide moiety. Indeed, this process resulted in (+)-daphlongamine E in 66% yield. Furthermore, *m*-CPBA treatment of (+)-daphlongamine E resulted in its *N*-oxide derivative, (+)-calyciphylline R.

### 4.9. Total Syntheses of (−)-Daphlongamine H and (−)-Isodaphlongamine H by Sarpong’s Group

**Scheme 37:**
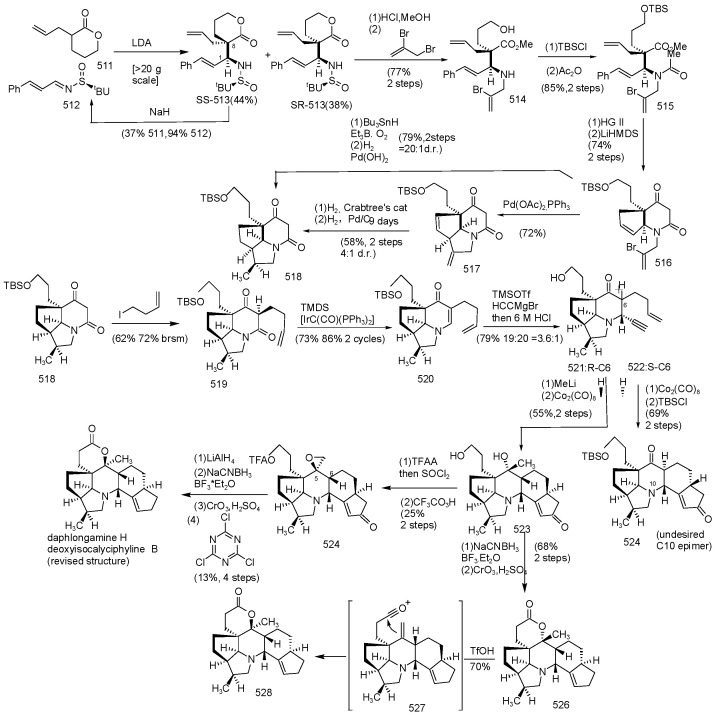
Total Syntheses of (−)-Daphlongamine H and (−)-Isodaphlongamine H by Sarpong’s group.

Ellman and colleagues’ groundbreaking research in 2010 demonstrated that the introduction of ester enolates to *N*-*tert*-butanesulfinyl imines yielded favorable diastereoselectivity at the β-amino stereocenter [192]. However, accurately predicting selectivity outcomes at the α-center remains a challenging task, particularly when utilizing fully substituted unsymmetrical enolates. Therefore, for their complete synthesis of (−)-daphlongamine H and (−)-isodaphlongamine H, Sarpong’s group initially examined various imines and enolates for suitable precursor compounds [193]. They ultimately selected allylated valerolactone **511** and sulfinyl imine **512**. As shown in Scheme 37, treating **512** with a lithium enolate derived from **511** resulted in an interesting transformation in the β-amino lactones *SS*- and *SR*-**513** through Mannich–retro-Mannich equilibrium. After chromatographic separation, the undesired β-amino lactone *SS*-**513** was recycled to regenerate **511** and **512** [193].

Next, *SR*-**513** was treated with HCl in MeOH, which cleaved the sulfinyl group and methanolized the lactone group. The intermediate ammonium salt was then alkylated to afford vinyl bromide **514**, which yielded amide **515** after silylation of the hydroxy group and acetylation of the secondary amine. LiHMDS was used to induce Dieckmann condensation on the resulting intermediate, thus forming bromo bicyclic compound **516**. To synthesize the tricyclic structure, an intramolecular Heck coupling reaction was conducted to obtain diene **517**. A two-step procedure involving Crabtree’s catalyst under an H_2_ atmosphere (50 atm) and subsequent heterogeneous hydrogenation yielded **518** (dr = 4:1).

Further, synthetic efforts focused on constructing the E and F rings of (−)-daphlongamine H and (−)-isodaphlongamine H. The alkylation of **518** resulted in alkene **519**. The researchers then reduced the δ-lactam carbonyl group of **519** to obtain enaminone **520** through elimination. By activating enaminone **520** using trimethylsilyl triflate (TMSOTf) [194] and adding ethynylmagnesium bromide (HCCMgBr), researchers generated a silyl enol ether. Subsequent hydrolysis during the workup process resulted in C6-epimeric enynes **521** and **522** in 79% overall yield. Enyne **522** underwent a Pauson–Khand reaction, resulting in the formation of enone **524** after the silylation of its primary hydroxy group. Treating **521** with excess MeLi also triggered a Pauson–Khand reaction, resulting in the formation of pentacyclic enone **523** with the desired orientation at the 10-Hα position. The enone moiety in **523** was efficiently deoxygenated using excess NaCNBH_3_ and a Lewis acid as a facilitator to produce the corresponding cyclopentene through a one-step reaction. Finally, the *cis*-lactone formation was achieved through Jones oxidation, completing the synthesis of isodaphlongamine H.

The researchers hypothesized that subjecting **526** to acidic conditions might trigger a series of reactions involving elimination–hydroacyloxylation for the biosynthesis of other calyciphylline B-type alkaloids [195]. Specifically, they expected it to form a mixture of deoxycalyciphylline B, deoxyisocalyciphylline B, daphlongamine H, and isodaphlongamine H. However, upon treating **526** with excess trifluoroacetic acid (TfOH) in nitromethane, the only productive conversion product was enone **528**. Therefore, the researchers suggested that formal dehydration occurred owing to the Prins-type cyclization of an acylium intermediate (**527**).

Ultimately, the synthesis of daphlongamine H encompassed the formal inversion of the stereochemistry of the tertiary alcohol in pentacyclic enone **523**. To achieve this, **523** was treated with TFAA and then SOCl_2_ to protect the primary hydroxy group and eliminate the tertiary hydroxy group. Epoxide **524** was obtained by reacting the exocyclic alkene with trifluoroperacetic acid (TFPAA; CF_3_CO_3_H) [196]. The subsequent epoxide opening at the terminal position was achieved by utilizing LiAlH_4_, followed by deoxygenation under established conditions. After performing Jones oxidation on the resulting amino diol, the researchers obtained *trans*-seco acid daphlongamine H. However, unlike **526**, which possessed a *cis*-lactone ring, this highly polar compound did not readily undergo lactonization. In the end, the researchers identified cyanuric chloride as a suitable compound [197] for bond creation. This afforded **528**, which possessed a *trans*-lactone ring, characterized by significant strain and sensitivity. Interestingly, the initial NMR spectrum of **528** did not match that reported for daphlongamine H [198]; instead, it exhibited remarkable similarity to that reported for deoxyisocalyciphylline B. This total synthesis of daphlongamine H from deoxyisocalyciphylline B calls for additional exploration into the suggested biosynthetic pathway for all calyciphylline B-type alkaloids.

### 4.10. Total Synthesis of (−)-Caldaphnidine O by Xu’s Group

**Scheme 38:**
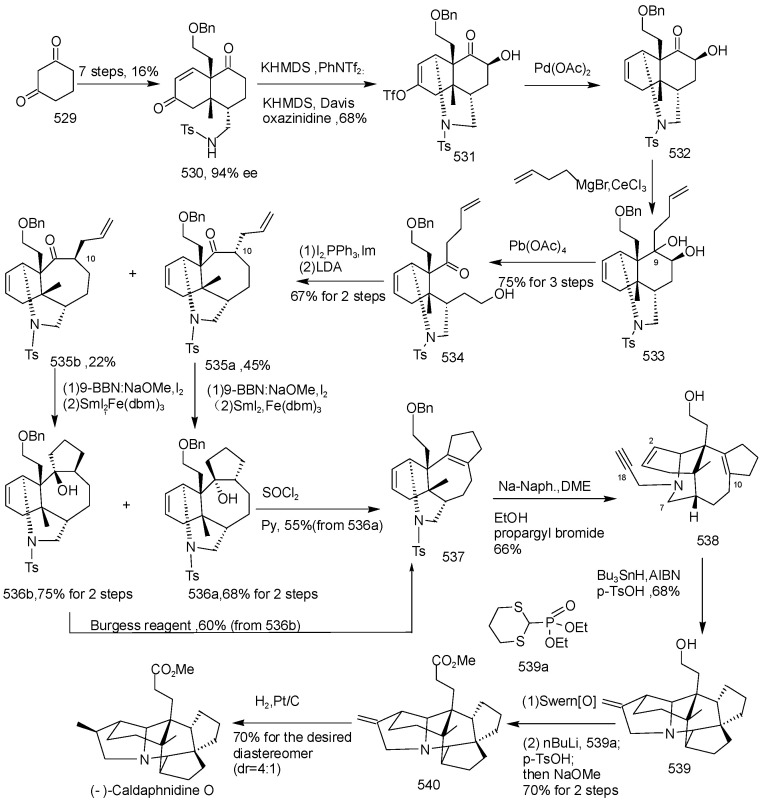
Total synthesis of (−)-caldaphnidine O by Xu’s group.

In 2019, Xu’s group achieved the total synthesis of (−)-caldaphnidine O [199]. As outlined in Scheme 38, the synthesis was initiated using the well-established chiral synthon **530** (94% ee), which was obtained from a seven-step transformation of 1,3-cyclohexanedione **529** in 16% overall yield [200]. The treatment of sulfonylamide diketone **530** with KHMDS and PhNTf_2_, followed by additional KHMDS and Davis’s oxaziridine, facilitated both the desired intramolecular aza-Michael addition reaction and α-hydroxylation in a single step, thus forming tricyclic compound **531** as the sole diastereomer. Pd(0)-mediated reduction [201] converted the enol triflate motif in **531** into alkene derivative **532**. The treatment of α-hydroxyl ketone **532** with a homoallyl cerium reagent [202], obtained in situ by mixing homo-allylMgBr with CeCl_3_, resulted in the quantitative formation of diol **533**. The diol moiety in **533** was subjected to oxidative cleavage, followed by selective reduction of the aldehyde group. This reaction effectively produced primary alcohol **534**.

Taking inspiration from Shvartsbart and Smith’s impressive synthesis of (−)-calyciphylline N [166], primary alcohol **534** was transformed into its corresponding alkyl iodide and then treated with LDA. As a result, **535a** and **535b** were obtained as C10 diastereomers (**535a**:**535b** = 2:1) with the desired seven-membered ring moiety. Both **535a** and **535b** were efficiently transformed into the radical cyclization precursor **537** through a concise three-step process. Compound **535a** was generated in two steps by the reaction of 9-BBN/NaOMe/I_2_ under Molander’s conditions (SmI_2_ and tris(dibenzoylmethanato)iron(III) (Fe(dbm)_3_)) to produce cyclopentanol **536a**. An attempt was made to replicate Knochel’s protocol [203]. Compound **535b** underwent a two-step conversion under Molander’s optimized reaction conditions to form cyclopentanol **536b** in 75% yield. Subjecting **536a** to SOCl_2_/pyridine significantly improved the alkene yield, whereas **536b** showed poor results under the same conditions. By contrast, exposing **536a** to Burgess reagent resulted in minimal production of cyclopentene **537**, whereas it effectively converted **536b** to **537** in 57–60% yield.

Next, sodium naphthalenide was used to remove both the *N*-tosyl and *O*-benzyl groups of **537**, followed by in situ *N*-propargylation to convert sulfonylamide **537** into dienyne **538**. Notably, **538** is a crucial precursor for radical cyclization. Diene **538** was exposed to Bu_3_SnH and AIBN and then acid-hydrolyzed to generate **539**. Subsequently, Swern oxidation was employed to convert the primary alcohol group in **539** into its corresponding aldehyde. Expanding on their previous discoveries in the synthesis of dapholdhamine B, the researchers utilized an HWE reaction with *n*-BuLi and phosphonate **539a** [204], followed by sequential acidic and basic treatments, resulting in the formation of carboxylic acid methyl ester **540**. Finally, through selective hydrogenation [205] of the C18–C20 alkene group in **540** from its convex side as a confined substrate, the researchers accomplished the first-ever synthesis of bukittinggine-type alkaloid (−)-caldaphnidine O.

### 4.11. Total Synthesis of (−)-Daphnezomines A and B by Li’s Group

**Scheme 39:**
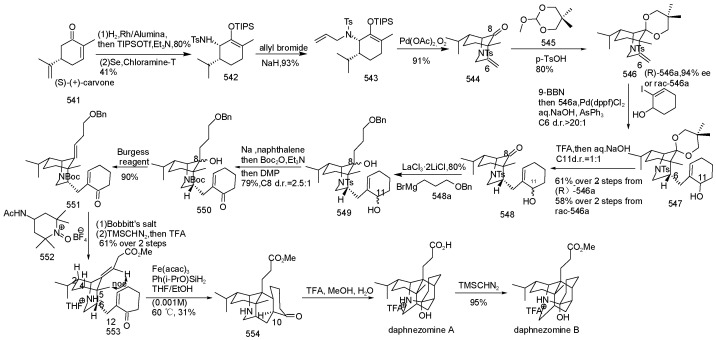
Total Synthesis of (−)-Daphnezomines A and B by Li’s group.

The complete synthesis of (−)-daphnezomines A and B has also been achieved [206]. As illustrated in Scheme 39, the synthesis initially aimed to generate the azabicyclo[3.3.1]nonane ring system (**544**). Starting from (*S*)-(+)-carvone (**541**), the unsaturated bond was globally hydrogenated, and the resulting ketone was reacted with triisopropylsilyl triflate (TIPSOTf) and Et_3_N [206]. Subsequently, the desired amination product, **542**, was produced using freshly prepared Sharpless amination reagents [207]. Compound **542** was subjected to NaH treatment and the subsequent addition of allyl bromide, thus forming **543**. Taking inspiration from Magnus’s elegant investigation [207], the researchers achieved the synthesis of **544** via the Pd(OAc)_2_ catalysis (20 mol%) of **543** in an O_2_ atmosphere [206]. By treating **544** with 2-methoxy-5,5-dimethyl-1,3-dioxane(**545**) under *p*-TsOH catalysis at 50 °C, the researchers efficiently formed bulky ketal **546** in 80% yield. The desired product, **547**, was synthesized by treating **546** with 9-BBN, followed by standard Suzuki–Miyaura coupling [208].

After extensive experiments to optimize the reaction conditions, it was discovered that the treatment of **547** with TFA produced **548** via the formation of a relatively stable C11 trifluoroacetate intermediate and the subsequent removal of 1,3-dioxane. The trifluoroacetate intermediate could be easily hydrolyzed through a basic workup process. This approach yielded good results; however, it produced C11 diastereomers of **548** in a 1:1 ratio. The researchers then implemented a two-step process that commenced with the initial addition of a nucleophile to the ketone, followed by subsequent dehydration. Ketone **548** was then combined with Grignard reagent **548a** at C8 to produce **549**.

The focus then shifted toward constructing the azaadamantane ring system. The tosyl (Ts) group was eliminated, and a *tert*-butyloxycarbonyl (Boc) group was introduced to protect the resulting amine with available functionality. Subsequently, the C11 alcohol underwent Dess–Martin oxidation to generate enone **550**. Notably, the two diastereomers eventually combined into a single compound via dehydration facilitated by the Burgess reagent, affording the desired product **551** in impressive yield (90%). Surprisingly, the oxidation of **551** with Bobbitt’s salt (**552**) resulted in the direct formation of the carboxylic acid with good chemoselectivity [209]. Compound **553** was obtained through the esterification of this acid with TMSCHN_2_ and the subsequent deprotection of the Boc group using TFA. On the gram scale, the overall yield of **553** from **551** was 61%.

The focus then shifted toward the challenging task of 6-*endo*-*trig* cyclization. The researchers explored three different approaches for obtaining either **554a**, **554**, or daphnezomine B: (i) utilizing a Lewis acid to promote ene cyclization [210]; (ii) employing base-mediated anionic cyclization; and (iii) initiating hydrogen atom transfer-induced radical conjugate addition [211]. Unfortunately, initial attempts with the first two reaction types did not yield satisfactory results, possibly because of steric crowding at C8. However, the desired cyclization was accomplished by Baran’s hydrogen atom transfer-initiated radical conjugate addition, which led to the exclusive formation of a transfused adduct, as observed in **554**. The application of TFA to keto amine **554** resulted in the conversion of its C10 epimer, thus forming daphnezomines A and B. These compounds feature a TFA-locked azaadamantane core. Although the NMR data of daphnezomine A·TFA varied slightly compared to that of the natural zwitterion form of daphnezomine A, the treatment of daphnezomine A·TFA with TMSCHN_2_ yielded daphnezomine B·TFA in impressive yield (95%).

### 4.12. Total Synthesis of Dapholdhamine B and Dapholdhamine B Lactone by Xu’s Group

**Scheme 40:**
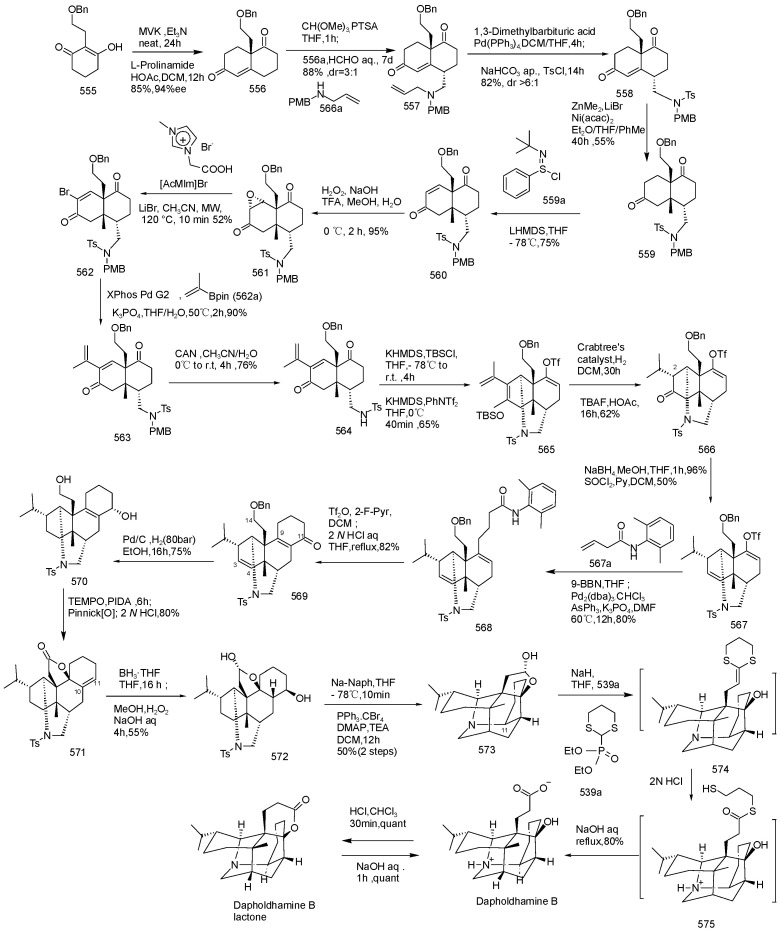
Total Synthesis of Dapholdhamine B and Dapholdhamine B Lactone by Xu’s group.

In 2019, J. Xu’s group reported the complete synthesis of dapholdhamine B and its lactone derivative, dapholdhamine B lactone [200]. As shown in Scheme 40, the synthesis was initiated by the l-prolinamide-catalyzed asymmetric Robinson annulation of **555**, affording diketone **556** (85% yield, 94% ee). A reactive methyl enol ether was selectively formed, followed by a vinylogous Mannich reaction [212], resulting in the production of tertiary amine **557**. Subsequently, **557** underwent deallylation and tosylation to afford sulfonylamide **558**. Luche’s conditions [213] facilitated conjugate addition, thus forming the crucial quaternary center. This resulted in the exclusive production of diketone **559** as a single diastereomer. Treating **559** with LiHMDS selectively formed the corresponding lithium enolate, which was then reacted with sulfinimidoyl chloride **559a** for Mukaiyama dehydrogenation [214]. As a result, the desired enone **560** was obtained in 75% yield.

An optimized method for α-bromination was developed based on a previously reported example. The researchers employed epoxide **561**, which was obtained from enone **560** through epoxidation, and LiBr as a bromide source. Other bromide sources proved ineffective, either showing no reaction, leading to decomposition, or producing only trace amounts of **562**. Notably, microwave irradiation improved the yield from 30% to 46–52%, thereby providing a sufficient quantity of vinyl bromide **562** for further investigation. Diene **563** was synthesized in high yield (90%) by Suzuki coupling between **562** and boronate **562a** with XPhos Pd G2 as the catalyst. The *p*-methoxybenzyl (PMB) group of **563** was then oxidatively removed to obtain sulfonylamide **564** in 76% yield. Enol triflate **565** was synthesized by reacting the silylenol ether obtained from the intramolecular aza-Michael addition reaction with excess KHMDS and PhNTf_2_. Ketone **566** was then produced via homogeneous hydrogenation of **565** using Crabtree’s catalyst [215], followed by in situ treatment with TBAF/HOAc. Compound **567** was formed by reducing and eliminating the carbonyl group in **566**. Finally, crucial amide intermediate **568** was synthesized via Suzuki coupling between enol triflate **567** and the borane derived from treating amide **567a** with 9-BBN. Next, tetracyclic compound **569** was obtained from **568** with an efficiency of 82% via Huang’s amide-activation–annulation in Tf_2_O/2-fluoropyridine. The resulting imine intermediate then underwent acid hydrolysis.

Building upon this significant finding, it was postulated that **569** could be transformed into tetracyclic diol **570** through a single-step process involving high-pressure hydrogenation/hydrogenolysis of its C3=C4 double bond, C11 ketone, and C14 *O*-benzyl group. Subsequently, a sequential process using TEMPO/PIDA and Pinnick oxidation was employed to selectively oxidize the primary alcohol at C14, resulting in the formation of lactone **571** through an anticipated S_N_2′-type reaction. Subsequent hydroboration of the C_10_=C_11_ double bond followed by oxidation led to the synthesis of **572**. By utilizing sodium naphthalenide for *N*-tosyl group removal and an S_N_2-type reaction, the researchers obtained **573** with a distinctive azaadamantane core structure. After a thorough investigation, the researchers achieved standardization of lactol **573** and employed an HWE reaction involving NaH and phosphonate **539a** to generate intermediate **574**. Subsequently, **574** underwent acid hydrolysis in a one-pot process to form thioester **575**. Notably, **574** and **575** were not isolated separately, as their subsequent basic hydrolysis led directly to the efficient synthesis of dapholdhamine B.

The synthetic product could not be compared directly with authentic dapholdhamine B via NMR owing to pH sensitivity issues. Therefore, a small quantity of synthetic dapholdhamine B was treated with HCl, resulting in the quantitative formation of dapholdhamine B lactone (The basic hydrolysis of dapholdhamine B lactone also yielded dapholdhamine B quantitatively). Through comprehensive NMR analysis of dapholdhamine B lactone, along with the unequivocal structural assignment of intermediate **573**, the researchers confirmed that the synthetic product was dapholdhamine B.

### 4.13. Total Syntheses of Daphnezomine L-Type and Secodaphniphyllinetype Daphniphyllum Alkaloids by J. Xu’s Group

**Scheme 41:**
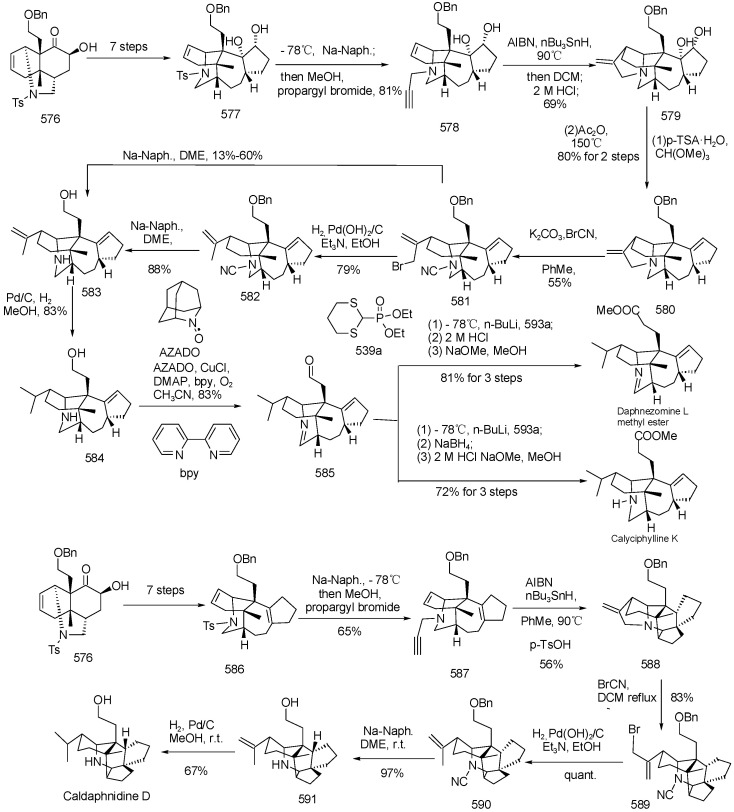
Total Syntheses of Daphnezomine L-type and Secodaphniphyllinetype Daphniphyllum Alkaloids by J. Xu’s group.

The total synthesis of daphnezomine L- and secodaphniphylline-type alkaloids utilizing late-stage C–N bond activation was reported by J. Xu’s group in 2022 [216]. As shown in Scheme 41, the approach commenced with widely used intermediate **576**, which was transformed into the desired tetracyclic diol **577** through a seven-step synthesis [200]. Specifically, **577** was exposed to sodium naphthalenide followed by propargylation, resulting in propargyl tertiary amine **578**. Compound **578** underwent enyne cyclization to afford pentacyclic amine **579**. An effective olefination method was employed to convert the diol structure in **579** to a trisubstituted alkene in **580** in 80% total yield. The expected ring-opening product, **581**, was obtained via the von Braun reaction of **580**, albeit with only a 55% yield. In addition, there were potential reproducibility issues observed during multiple attempts using sodium naphthalenide.

Nevertheless, a robust two-step procedure was employed to synthesize **583** from **581** in 70% overall yield. This involved hydrogenolysis of the C–Br bond using H_2_ and Pd(OH)_2_/C in the presence of Et_3_N and MeOH to form **582**, followed by global removal of the N–CN and O–benzyl (OBn) groups utilizing a sodium naphthalenide solution. Subsequently, amino alcohol **584** was obtained through hydrogenation of the alkene moiety with two substituents in **583** using Pd/C, H_2_, and MeOH in 83% yield. Further oxidation afforded imine-aldehyde **585**. The aldehyde group in **585** was subsequently subjected to a HWE reaction, followed by hydrolysis of the corresponding ketene dithioacetal group. This resulted in the formation of a methyl ester carboxylic acid group, which was utilized for synthesizing daphnezomine L methyl ester. Calyciphylline K was synthesized using a similar approach with an additional imine reduction step, with a 72% yield over three steps.

The synthesis of caldaphnidine D commenced from intermediate **586**, which is readily obtainable from intermediate **576** in seven synthetic steps. The *N*-tosyl group in **586** was substituted with a propargyl group to yield enyne **587**. Enyne derivative **587** was further transformed into hexacyclic compound **588** through a radical cyclization cascade employing AIBN and Bu_3_SnH. Reorganizing the experimental steps, **588** was smoothly transformed into pentacyclic compound **589** using von Braun’s conditions. Finally, **589** underwent a series of consecutive transformations, including C–Br bond reduction, N–CN and O–Bn group removal using sodium naphthalenide, and finally, 1,1-disubstituted alkene motif hydrogenation. These modifications resulted in caldaphnidine D with an overall efficiency of 65% across three steps.

### 4.14. Total Synthesis of Hybridaphniphylline B by Li’s Group

**Scheme 42:**
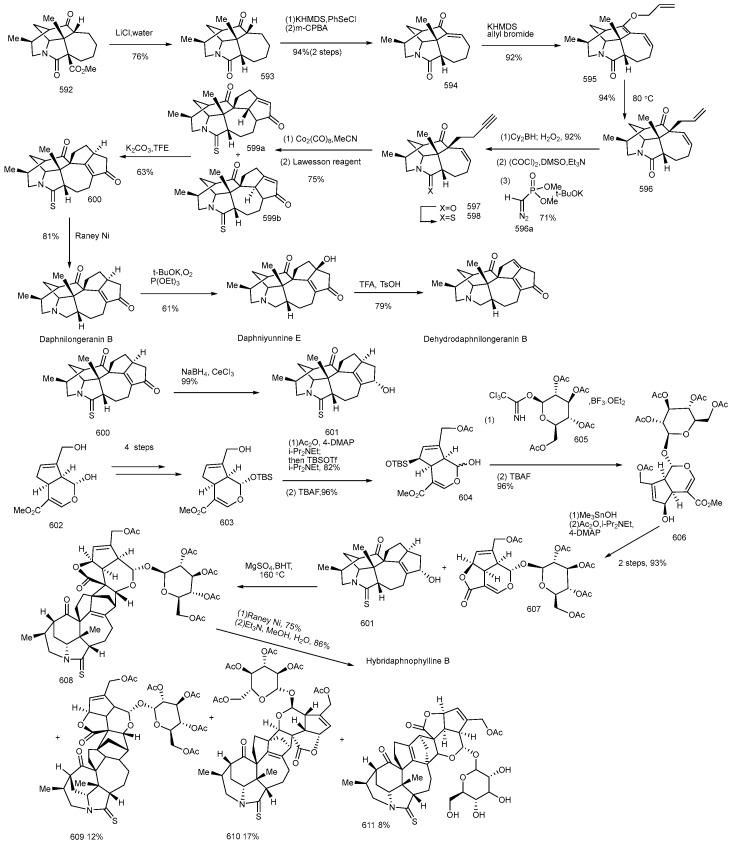
Total Synthesis of Hybridaphniphylline B by Li’s group.

The first total synthesis of hybridaphniphylline B, a DA with 11 rings and 19 stereocenters, was reported by A. Li’s group in 2018 [164]. The synthesis involved a late-stage intermolecular Diels–Alder reaction to combine a highly developed cyclopentadiene and asperuloside tetraacetate (**607**). As shown in Scheme 42, **592** was subjected to Krapcho demethoxycarbonylation to form **593**. Subsequent α-selenation and oxidative elimination led to the generation of α,β-unsaturated enone **594**. Treatment with KHMDS and allyl bromide resulted in the formation of dienol ether **595**. The Claisen rearrangement of **595** occurred smoothly using a MeOH/H_2_O solvent at 80 °C, thus producing **596**. However, no Cope rearrangement occurred under these conditions. The terminal C=C double bond in **596** was selectively hydroborated using diethylborane (Cy_2_BH), followed by oxidation to yield a primary alcohol. Subsequent Swern oxidation and Seyferth–Gilbert homologation with **596a** led to the formation of alkyne **597**. Then, the treatment of **597** with Lawesson’s reagent afforded **598**. A study on Pauson–Khand reaction conditions revealed that MeCN effectively promoted the transformation from the alkyne dicobalt complex formed from **598** and Co_2_(CO)_8_ to obtain **599a** and **599b** in a 2.4:1 ratio with a yield of approximately 73%. Further treatment with K_2_CO_3_/TFE led to the migration of the C=C bonds, affording enone **600** with higher substitution, in 63% overall yield from **598**. Subsequently, the reduction of thioamide **600** using Raney Ni produced daphnilongeranin B. Both racemic and enantioenriched forms of daphnilongeranin B were synthesized via the described route.

The treatment of daphnilongeranin B with *t*-BuOK and O_2_ in the presence of triethyl phosphite (P(OEt)_3_) yielded diastereomerically pure daphniyunnine E in 61% yield. Dehydration was achieved by treating the TFA salt of daphniyunnine E with *p*-TsOH, affording dehydrodaphnilongeranin B in a high yield (79%). Interestingly, enone **600**, the immediate precursor of daphnilongeranin B, underwent Luche reduction to yield allylic alcohol **601**. Subsequently, asperuloside tetraacetate (**607**) was synthesized as a dienophile. Compound **603**, obtained from (+)-genipin (**602**), underwent a series of chemical modifications, including acetylation, silylation, and selective deprotection of the less-hindered silyl ether. This resulted in the formation of lactol **604** as a mixture of two anomers in roughly equal proportions. The glycosylation between **604** and trichloroacetimidate **605**, followed by desilylation, led to the production of **606** with only one stereoisomer. Despite undergoing partial deacetylation upon exposure to trimethyltin hydroxide (Me_3_SnOH), reacetylation yielded **607**. To generate the dienes required for further reactions from precursor **601**, the researchers developed a convenient procedure using MgSO_4_ as a mild yet efficient dehydrating agent under elevated temperature conditions. By employing MgSO_4_ and butylated hydroxytoluene (BHT) at 160 °C, cyclopentadiene was generated from **601** and subsequently reacted with **607** to produce cycloadducts **608**–**611**. Finally, the reduction of **608** using Raney Ni followed by global deacetylation resulted in hybridaphniphylline B.

### 4.15. Total Synthesis of Longeracinphyllin A by A. Li’s Group

**Scheme 43:**
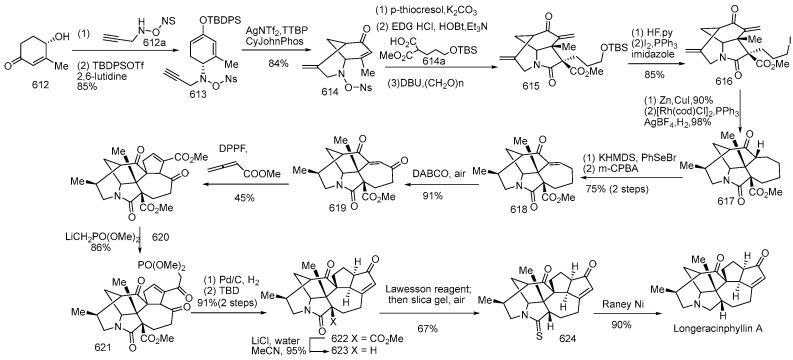
Total Synthesis of Longeracinphyllin A by Li’s group.

A. Li’s group achieved the complete synthesis of longeracinphyllin A in 2017 [145]. As shown in Scheme 43, alcohol **612** was subjected to a widely recognized two-step process to form alkynyl silyl enol ether **613**. In the presence of silver triflimide (AgNTf_2_) and CyJohnPhos, the cyclization of **613** led to the formation of **614** in good yield (84%), accompanied by a minor product (5% yield) via a less favorable 7-*endo*-*dig* cyclization pathway. The nosyl group was removed from **614** using TTBP, a 4 Å molecular sieve, and CyJohnPhos, followed by condensation with **614a**, treatment with DBU at 95 °C, and finally a one-pot reaction with paraformaldehyde to afford **615**. Compound **616** was efficiently synthesized from **615** through sequential desilylation and iodination. Based on their prior knowledge of the asymmetric hydrogenation of unfunctionalized olefins, the researchers employed a Rh-based catalytic system to achieve remarkable facial selectivity. As a result, the crucial intermediate (**617**) was obtained in 98% yield as the sole detectable diastereomer. The ketone underwent α-selenation, followed by oxidative elimination, resulting in the synthesis of α,β-unsaturated enone **618** with remarkable overall effectiveness.

The subsequent treatment of **618** with 1,4-diazabicyclo[2.2.2]octane (DABCO) in the air led to the impressive formation of enedione **619**. The utilization of DPPF as a catalyst highly favored the [3+2] pathway, resulting in **620** in 45% yield at the gram scale. The treatment of **620** with excess LiCH_2_PO(OMe)_2_ resulted in the formation of β-ketophosphonate **621**. Subsequent hydrogenation and intramolecular HWE olefination afforded hexacycle **622**. Krapcho demethoxycarbonylation with MeCN resulted in **623** in 95% yield. Thiolation of both the enone and lactam carbonyls with Lawesson’s reagent, followed by oxygenation of the more labile thioenone in air, led to the efficient formation of thioamide **624**. Finally, reduction using Raney Ni produced longeracinphyllin A.

### 4.16. Synthesis of (+)-Caldaphnidine J Using an Asymmetric Approach by Xu’s Group

**Scheme 44:**
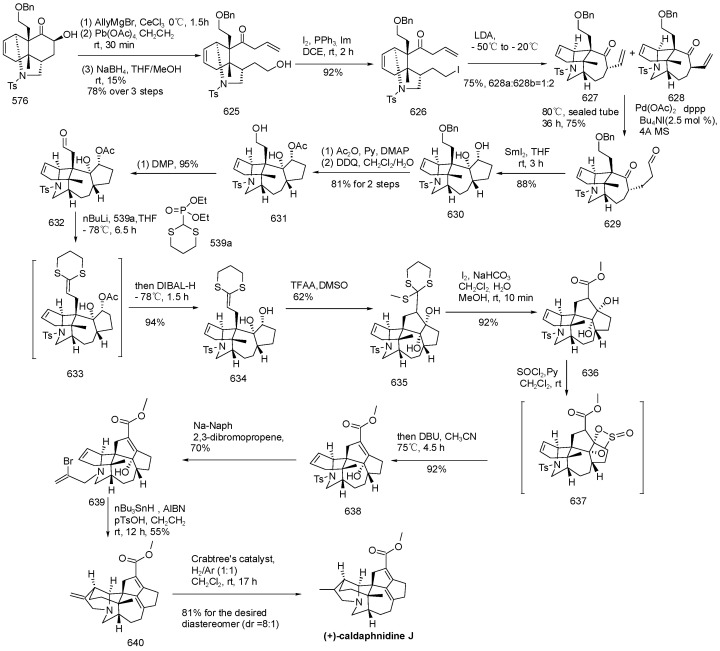
Synthesis of (+)-caldaphnidine J using an asymmetric approach by Xu’s group.

The asymmetric total synthesis of the yuzurimine-type alkaloid (+)-caldaphnidine J was accomplished by Xu’s group in 2020 [217]. In this synthesis (Scheme 44), ketone **576** was treated with allylMgBr in the presence of CeCl_3_ to form a diol intermediate, which was then subjected to Pb(IV)-mediated oxidative cleavage. Subsequent reduction using NaBH_4_ resulted in the production of β,γ-unsaturated ketone **625**, which exhibited some degree of instability. To address this issue, alkyl iodide **626** was promptly generated through the iodination of ketone **625**. Subsequently, the treatment of **626** with LDA induced intramolecular alkylation, thus forming α-vinyl functionalized ketones **628** and **627** in 25% and 50% yield, respectively.

Regioselective hydroformylation of the terminal alkene moiety in **627** using Shi’s protocol afforded aldehyde **629** in 75% yield. Subsequently, diol **630** was synthesized through an intramolecular pinacol coupling reaction mediated by SmI_2_. The secondary hydroxyl group was selectively acylated and then subjected to 2,3-dichloro-5,6-dicyano-1,4-benzoquinone (DDQ)-mediated debenzylation to afford primary alcohol **631**. Further oxidation using DMP converted alcohol **631** into aldehyde **632**. Phosphonate **539a** was utilized in HWE homologation, followed by a one-step reduction using DIBAL-H. This synthetic route yielded ketene dithioacetal **634** in an impressive 94% yield.

The cyclization of diol **634** was accomplished by TFAA/DMSO-mediated Swern oxidation, resulting in the formation of a ketone functional group. Subsequently, **635** was synthesized by introducing Me_2_S to the sulfonium intermediate and removing the methyl groups. The conversion from the 2-(methylthio)-1,3-dithiane moiety to methyl ester **636** proceeded smoothly through treatment with methanolic iodine. *cis*-Diol **636** reacted with SOCl_2_ to afford dialkyl sulfite **637**, which underwent E2cB elimination upon treatment with DBU, resulting in the desired allylic alcohol **638**. After removing the tosyl group from **638**, a one-pot alkylation process was conducted, leading to the synthesis of vinyl bromide **639**. The key tetrahydropyrrole ring in **640** was assembled through radical cyclization mediated by AIBN/Bu_3_SnH. Upon acidic workup, the C9 hydroxyl group was eliminated, thus forming a conjugated diene. A highly selective hydrogenation process (H_2_/Ar = 1:1) utilizing Crabtree’s catalyst resulted in (+)-caldaphnidine J in 81% yield with remarkable regio- and diastereoselectivity (dr = 8:1).

### 4.17. Total Synthesis of Daphgraciline by Li’s Group

**Scheme 45:**
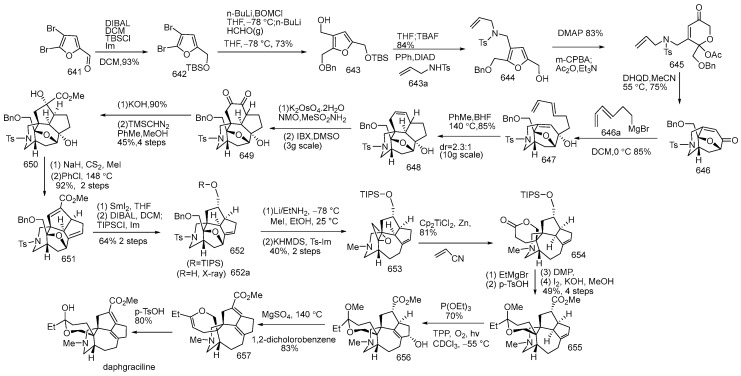
Total Synthesis of Daphgraciline by C.-C. Li’s group.

C.-C. Li and colleagues reported the total synthesis of the yuzurine-type alkaloid daphgraciline in 2022 [218]. As shown in Scheme 45, **641** was reduced using DIBAL-H and protected with TBSCl, resulting in **642** in 93% overall yield. Reactive exchanges of dibromofuran **642** with *n*-BuLi, followed by introductions of benzyl chloromethyl ether (BOMCl) and gaseous formaldehyde (HCHO), were performed sequentially to afford **643**. Compound **643** was then transformed into **644** via a Mitsunobu reaction with **643a** and subsequent deprotection. The utilization of *m*-CPBA in DCM facilitated the Achmatowicz rearrangement of **644**, which was subsequently acetylated in a one-pot reaction. This process resulted in the synthesis of **645** in 83% yield. Next, dihydroquinidine (DHQD) [219] was employed as a base catalyst at 55 °C for the desired type II [5+2] cycloaddition of **645**, thus forming **646**. By selectively adding **646a** to **646**, the researchers obtained **647** on a significant scale (15 g). Further progression involved the intramolecular Diels–Alder reaction of **647**, resulting in a mixture of **648** and its C15-diastereomer counterpart (dr = 2.3:1) in exceptional yield (85%). The dihydroxylation of a mixture of **648** and **648a** with potassium osmate(VI) anhydrous (K_2_OsO_4_), followed by oxidation with IBX, yielded diketone **649**.

Subsequently, the researchers attempted Wolff rearrangement [220] of **649** but discovered significant challenges. Therefore, the desired tetracyclic core **650** was obtained by ring-contraction and benzilic acid-type rearrangement [221] of **649**. Compound **650**, possessing two hydroxyl groups that were not bound to any other atoms, underwent double Chugaev elimination with remarkable efficiency, resulting in the formation of **651**. The researchers then achieved the chemo- and diastereoselective conjugated reduction of the C13=C14 olefin moiety in **651** using SmI_2_. This was succeeded by the reduction of the ester group using DIBAL-H and simultaneous protection with triisopropyl silane (TIPS) in a single-step reaction, thus forming **652** in 64% overall yield. Confirmation of the structure of **652** was achieved by X-ray crystallography of its precursor, **652a**.

Compound **652** was treated with Li in ethylamine (EtNH_2_), followed by the addition of MeI, resulting in the synthesis of the desired diol. Subsequently, subjecting the diol to KHMDS and tosyl-imidazole (Ts-Im) in THF led to the formation of epoxide **653**. The reductive epoxide cyclization between **653** and acrylonitrile exclusively yielded spirolactone **654** as a single diastereomer in 81% yield. Sequential treatment of **654** with ethylmagnesium bromide (EtMgBr) in Et_2_O, followed by *p*-TsOH in MeOH, led to the formation of a ketal alcohol. Subsequent oxidations using DMP and I_2_/KOH/MeOH converted the ketal alcohol to ester **655**. Expanding on their prior research [222], the researchers achieved the synthesis of alcohol **656** through a Schenck ene reaction utilizing TPP as the photosensitizer. This approach effectively addresses the synthetic challenge posed by the C9=C10 tetrasubstituted double bond. Taking inspiration from the groundbreaking research conducted by A. Li’s group [164], the researchers heated **656** to 140 °C in MgSO_4_, thus forming dehydrodaphgraciline (**657**) [223] bearing a C14=C15 tetrasubstituted double bond. Finally, the treatment of **657** with *p*-TsOH in THF/H_2_O afforded daphgraciline an 80% overall yield.

### 4.18. Total Synthesis of C_14_-epi-Deoxycalyciphylline H by Xu’s Group

**Scheme 46:**
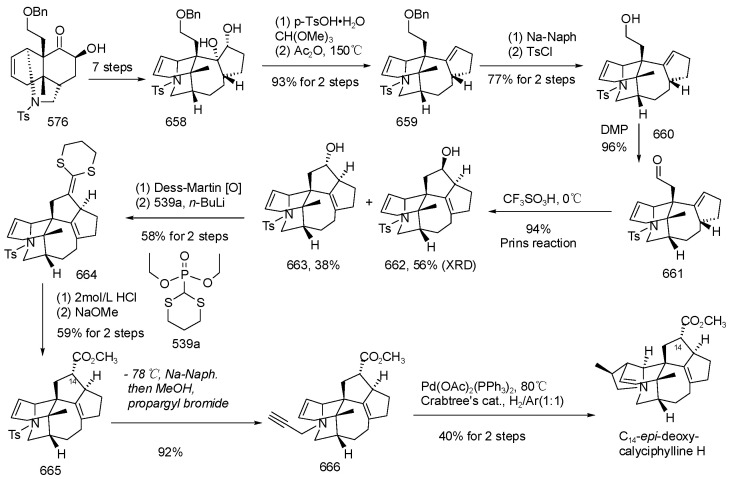
Total Synthesis of C_14_-epi-Deoxycalyciphylline H by Xu’s group.

Hu and Xu achieved the complete synthesis of C_14_-*epi*-deoxycalyciphylline H, which is widely considered to be a yuzurimine-type alkaloid, in 2024 [224]. As shown in Scheme 46, the investigation began with tricyclic compound **576**, which underwent a seven-step process involving ring expansion and cyclopentane formation to produce vicinal diol **658**. Subsequently, by utilizing Ando’s olefination conditions (*p*-TsOH and trimethyl orthoformate (CH(OMe)_3_) followed by Ac_2_O at 150 °C) [225], alkene **659** was efficiently derived from diol **658** in 93% yield. The benzyl group of **659** was eliminated using sodium naphthalenide; however, owing to partial *N*-detosylation, it was necessary to retosylate **659** to obtain a desirable yield of **660**.

To enhance the efficiency of the synthesis process, the primary hydroxyl group in **660** was oxidized by a straightforward Dess–Martin reaction, resulting in aldehyde formation (**661**). Subsequently, **661** was subjected to acidic conditions (TfOH at −0 °C) to trigger a Prins reaction involving the aldehyde and alkene motifs, resulting in the fabrication of alcohol motifs **662** and **663** (56% and 38% yield, respectively). The mixture of **662** and **663** was subjected to Dess–Martin oxidation, resulting in the corresponding ketone, which was homologated by an HWE reaction (**539a** and *n*-BuLi) to produce **664**. Compound **665** was then formed via hydrolysis of the ketene dithioacetal moiety in **664**. By replacing the *N*-tosyl group with a propargyl group, enyne **666** was obtained in high yield (92%). Finally, Pd-catalyzed enyne cycloisomerization [226] facilitated the formation of both the essential tetrahydropyrrole motif and the C3–C4 alkene motif in the corresponding diene, and selective hydrogenation employing H_2_ and Crabtree’s catalyst generated C_14_-*epi*-deoxycalyciphylline H.

### 4.19. Total Synthesis of (−)-Himalensine A by Dixon’s Group

**Scheme 47:**
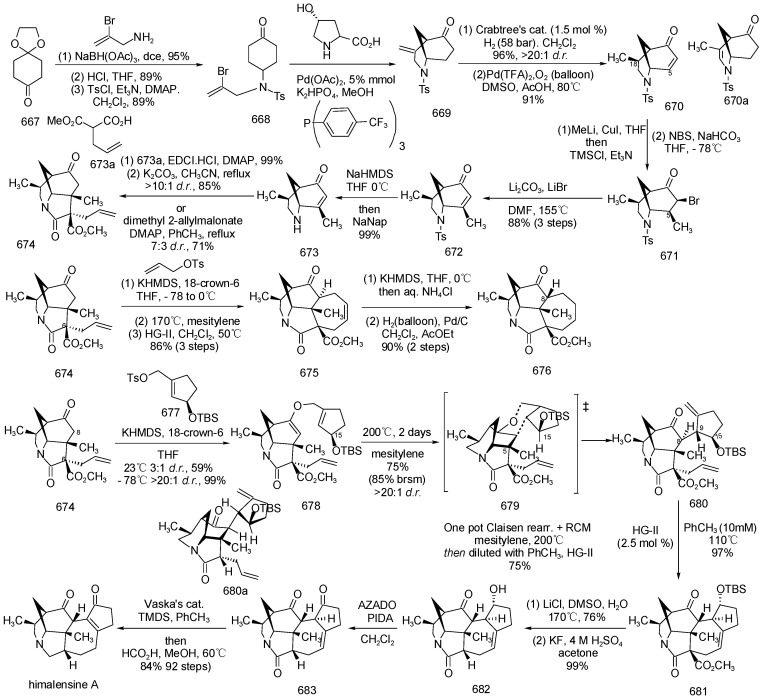
Total Synthesis of (−)-Himalensine A by Dixon’s group.

In 2023, Dixon’s team achieved the convergent enantioselective total synthesis of himalensine A in just 18 steps [227]. This was made possible through the use of a carefully selected method for constructing the morphan core. Specifically, the researchers employed a co-catalyzed desymmetrization technique involving Pd and hydroxyproline, as well as cyclohexanones and vinyl-bromide tethers. As shown in Scheme 47, **668** was synthesized by the reductive amination of **667** using 2-bromoprop-2-en-1-amine, followed by ketal hydrolysis and amine tosylation by standard procedures. The key vinylation reaction was easily scaled up, resulting in cyclized product **669** in 92% yield with 94% ee. The compound **669** was hydrogenated to **670** and **670a**. Next, enone **670** was reacted with Me_2_CuLi·LiI (formed in situ from MeLi and CuI) and TMSCl. The resultant silyl enol ether underwent bromination employing NBS to form **671**, followed by dehydrobromination utilizing Li_2_CO_3_ and LiBr to yield **672**. Subsequently, the tosyl group of **672** was selectively removed via treatment with sodium naphthalenide after protecting its extended sodium enolate form in situ. This involved the coupling of secondary amine **673** with malonate **673a** using EDCI·HCl [228]. The desired tricyclic compound (**674**) was obtained by treating the resulting malonamate with K_2_CO_3_ in MeCN.

Compound **674** was exposed to KHMDS at −78 °C, treated with allyl tosylate in the presence of 18-crown-6, and subsequently heated to 170 °C in mesitylene, thus forming a Claisen rearrangement product. Treating this product with Hoveyda–Grubbs II catalyst produced tetracyclic **675** through ring-closing metathesis in 86% overall yield over three steps. The application of KHMDS and a protic workup on **675** resulted in epimerization at C8. Finally, hydrogenation of the C8 epimerization product afforded **676**. Notably, **676** is a versatile intermediate for DA synthesis.

In another pathway, tricyclic ketone **674** was treated with KHMDS at low temperatures for deprotonation. The resulting enolate was then treated with a complex allyl tosylate (**677**), thus producing enol ether **678** with high efficiency. When heated to approximately 200 °C, **678** underwent Claisen rearrangement through a possible chair-like transition state (**679**), thereby creating two neighboring tertiary stereocenters. To determine the relative stereochemical configuration of **680**, an analogous compound (**680a**) was synthesized using the same methodology and analyzed by single-crystal X-ray diffraction.

By treating diene **680** with Hoveyda–Grubbs II catalyst, the D ring of himalensine A was effectively formed along with C8 epimerization. This led to a relatively thermodynamically stable bowl-shaped epimer, **681**, in satisfactory yield. The methyl ester group in **681** was eliminated through Krapcho decarboxylation, while acidic conditions were employed to cleave the TBS group to form **682**. To address the challenge of oxidizing sterically hindered alcohol **682**, the researchers utilized a combination of PIDA and 2-azaadamantane *N*-oxyl (AZADO) catalysis. Following purification on silica, the resulting oxidized product (**683**) exhibited alkene migration, which ultimately resulted in the formation of oxyhimalensine A. As a result, the formal synthesis of this natural product was successfully completed.

An alternative approach involved subjecting the crude oxidation product **683** to reduction conditions, as previously described for himalensine A. According to this procedure, the lactam in **683** was initially transformed into its corresponding silylated hemiaminal by utilizing Vaska’s complex in the presence of tetramethyldisiloxane (TMDS). Subsequently, through treatment with formic acid, it underwent further reduction to produce the desired pyrrolidine ring. Simultaneously, migration of the double bond resulted in conjugation with the carbonyl group on the cyclopentanone E ring. Thus, a complete synthesis of himalensine A [150] was accomplished in 20 steps with a 10% overall yield (18 steps and 9% yield after telescoping).

### 4.20. Distinct Total Syntheses of (−)-Daphnezomines A and B and (+)-Dapholdhamine B by Zhai’s Group

**Scheme 48:**
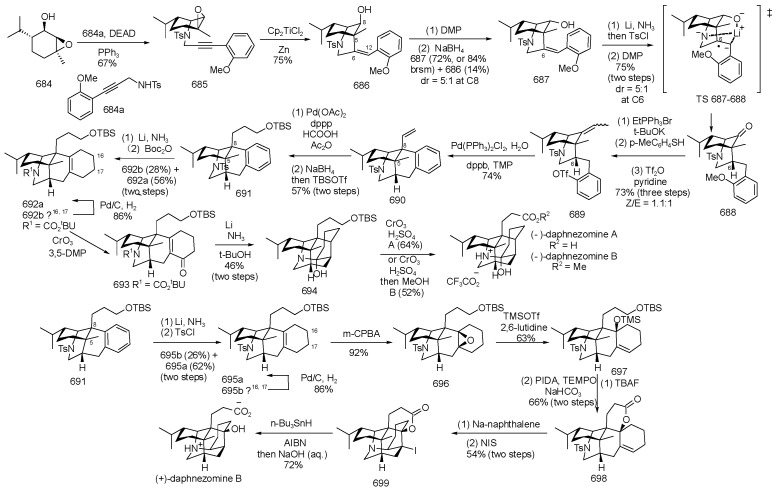
Distinct Total Syntheses of (−)-Daphnezomines A and B and (+)-Dapholdhamine B by Zhai’s group.

In 2023, Zhai’s team reported the distinct complete syntheses of (−)-daphnezomines A and B, as well as (+)-dapholdhamine B [229]. As shown in Scheme 48, the Mitsunobu reaction of known chiral alcohol **684** with known sulfonamide **684a**, which can be obtained from *o*-iodoanisole through Sonogashira coupling, afforded epoxide **685**. Product **686** was obtained by introducing epoxide **685** dropwise to a Ti(III) reagent (formed in situ from bis(cyclopentadienyl)titanium(IV) dichloride (Cp_2_TiCl_2_) and activated Zn powder) under dilute conditions. The oxidation of alcohol **686** using DMP, followed by reduction of the carbonyl group with NaBH_4_ in a one-pot reaction, led to the synthesis of **687** in 72% yield (or 84% yield with recycling of the starting material). Subsequent treatment of homoallylic alcohol **687** with Li/NH_3_(liq) in the presence of EtOH resulted in ketone **688** through a reaction with DMP.

The synthesis of **689** (*Z*/*E* = 1.1:1) was accomplished in approximately 73% overall yield through a series of reactions, including Wittig olefination between ketone **688** and EtPPh_3_Br, demethylation of the methyl ether utilizing *p*-Me-C_6_H_4_SH, and triflation employing Tf_2_O. Compound **690** underwent a two-step conversion to yield **691**. Two distinct free amines were produced, which were then protected using excess di-*tert*-butyldicarbonate (Boc_2_O), thus forming a mixture of carbamic–carbonic anhydrides **692a** (a monoene) and **692b** (a diene). Through Pd/C-catalyzed chemoselective hydrogenation, **692a** was selectively obtained as the sole product. Regioselective allylic oxidation of alkene **692a** at the least hindered position with CrO_3_ and 3,5-dimethylpyrazole afforded enone **693**. Treating enone **693** with Li/NH_3_(liq) in the presence of *t*-BuOH led to hemiketal **694** in approximately 46% overall yield over two steps. Finally, hemiketal **694** was subjected to Jones oxidation (CrO_3_/H_2_SO_4_) to form (−)-daphnezomine A in approximately 64% yield. Alternatively, by adding excess MeOH to the reaction mixture, (−)-daphnezomine B was obtained in 52% yield. (−)-Daphnezomines A and B were both converted into their respective trifluoroacetates using flash column chromatography with DCM/MeOH/TFA as the eluent.

To synthesize (−)-dapholdhamine B, **691** underwent Birch reduction of the benzene ring, while the tosyl (Ts) protecting group on the N atom was simultaneously removed through treatment with Li/NH_3_(liq). The resulting mixture, which contained two free amines, was then reacted directly with *p*-TsCl/Et_3_N to produce alkene **695a** and diene **695b** in 62% and 26% yield, respectively. Diene **695b** was subsequently converted into alkene **695a** via catalytic hydrogenation in 86% overall yield. Selective epoxidation from the convex face occurred during the *m*-CPBA-mediated epoxidation of alkene **695a**, leading to excellent stereoselectivity in the formation of epoxide **696**. Exposing **696** to TMSOTf in the presence of 2,6-lutidine led to the selective opening of the epoxide ring at the least hindered position. This resulted in allylic TMS ether **697** in moderate yield (63%) [230]. By global removal of the silyl groups and oxidization using PIDA/TEMPO, lactone **698** was obtained through oxidative cyclization. To ensure stability under acidic conditions, solid NaHCO_3_ was introduced prior to PIDA/TEMPO oxidation. Following reductive desulfurization, the resulting amine was treated with *N*-iodosuccinimide (NIS) to generate tertiary iodide **699** through a necessary 6-*endo*-*trig* haloamination. This transformation afforded the hexacyclic structure observed in (+)-dapholdhamine B. Ultimately, the dehalogenation of iodolactone **699** [231] was achieved using a radical reaction with AIBN and Bu_3_SnH, followed by saponification of the lactone moiety under basic conditions (15% NaOH(aq)/MeOH, 1:1 *v*/*v*). The overall synthetic strategy resulted in an impressive 72% yield for (+)-dapholdhamine B.

### 4.21. Total Synthesis of (±)- and (−)-Daphnillonin B by Li’s Group

**Scheme 49:**
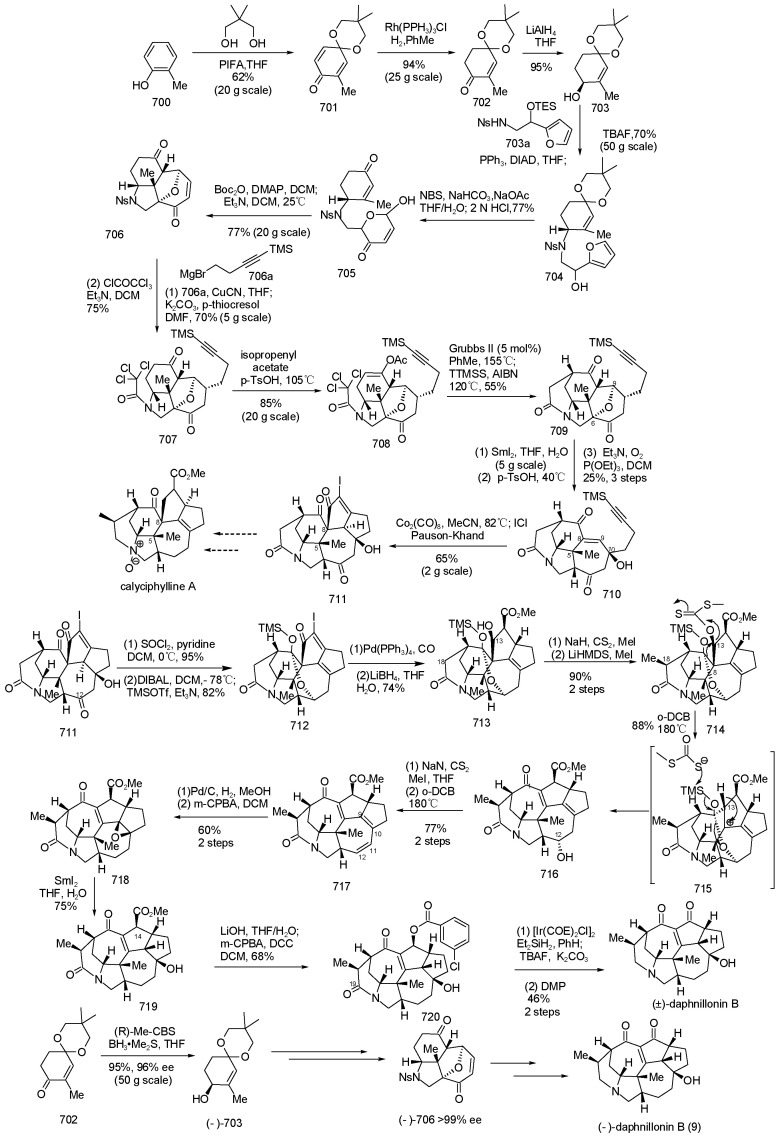
Total Synthesis of (±)- and (−)-Daphnillonin B by Li’s group.

In 2023, Li’s group achieved the total synthesis of daphnicyclidin-type alkaloids (±)- and (−)-daphnillonin B. These compounds contain a unique 7/6/5/7/5/5 ABCDEF hexacyclic core structure [232]. As shown in Scheme 49, the oxidative dearomatization of *o*-cresol (**700**) using 2,2-dimethylpropane-1,3-diol and phenyliodine(III) bis(trifluoroacetate) (PIFA) was conducted to form **701** in 62% yield. The regioselective hydrogenation of **701** using Wilkinson’s catalyst resulted in **702** in 94% yield. Compound **702** was then subjected to carbonyl group reduction using LiAlH_4_, thus forming **703** in 95% yield. A one-pot transformation involving a Mitsunobu reaction between **703** and reagent **703a**, followed by deprotection, afforded **704** in 70% yield (50 g scale). The Achmatowicz rearrangement of **704** utilizing NBS and subsequent workup with concentrated HCl (2 N) yielded **705** in 77% yield. Compound **705** underwent a series of reactions involving Boc_2_O and DMAP in DCM. This was followed by the addition of Et_3_N in DCM in the same reaction vessel. The result was the exclusive production of **706** as a single diastereomer in an impressive 77% yield.

Next, the diastereoselective addition of Grignard reagent **706a** to **706**, on a smaller scale, led to further transformations, including nitrobenzenesulfonyl (Ns) group deprotection and subsequent trichloroacetylation. Ultimately, this sequence yielded **707** with an overall efficiency of approximately 53%. To obtain enol acetate derivative **708**, ketone **707** underwent additional processing using isopropenylacetate under optimized conditions. Notably, when conducted on larger scales (e.g., 20 g), the desired enol acetate product exhibited excellent conversion rates and high yields (85%). Radical cyclization with Grubbs II catalyst proceeded smoothly on trichloroacetamide derivative **708**, followed by one-pot dechlorination to afford **709** in 55% yield. The C6–O bond in **709** was selectively broken by reduction using SmI_2_ (5 g scale). Subsequently, the resulting C9–OH group was eliminated with *p*-TsOH and then hydroxylated at C10 using O_2_ and Et_3_N. This led to the formation of **710** in satisfactory yield over three steps.

After extensive investigations, **710** underwent an intramolecular Pauson–Khand reaction when treated with Co_2_(CO)_8_ in refluxing MeCN. The subsequent iodination process utilizing ICl produced **711** as a single diastereomer. Compound **711** shows great potential as an advanced intermediate for synthesizing calyciphylline A-type alkaloids. To further modify **711**, it was treated with SOCl_2_ and pyridine in DCM, followed by a one-pot reaction of the resulting alcohol with TMSOTf and Et_3_N. These reactions collectively yielded **712** in a satisfactory 78% overall yield. The vinyl iodide in **712** was subjected to carbonylation using a Pd(PPh_3_)_4_ catalyst under a CO atmosphere at elevated pressure in a mixture of MeOH and THF. This was followed by diastereoselective reduction at positions 1 and 4, as well as positions 1 and 2, utilizing LiBH_4_, thus forming **713**. Subsequently, treatment of **713** with NaH/CS_2_/MeI led to diastereoselective methylation at C18, yielding **714** in 90% overall yield. Notably, heating **714** to 180 °C in *o*-dichlorobenzene (*o*-DCB) achieved the desired product (**716**) with high efficiency (88% yield). Compound **716** then underwent Chugaev elimination to produce **717** in approximately 77% overall yield. Finally, chemoselective hydrogenation, which targeted the C11–C12 olefin within **717**, and subsequent diastereoselective epoxidation focusing on the C9–C10 olefin moiety resulted in the formation of **718**. The treatment of **718** with SmI_2_ afforded **719** as a single diastereomer in 75% yield. Carboxylic ester **719** was then subjected to a series of reactions involving LiOH, *m*-CPBA, and DCC in DCM. As a result, **720** was obtained in 68% yield.

Finally, selective reduction of the C19 lactam moiety in **720** using chlorobis(cyclooctene)iridium(I) dimer ([Ir(coe)_2_Cl]_2_) and diethylsilane (Et_2_SiH_2_), followed by workup with TBAF and K_2_CO_3_ and subsequent oxidation with DMP, furnished (±)-daphnillonin B in 46% overall yield. Notably, standard Corey–Bakshi–Shibata reaction conditions transformed **702** into (−)-**703** as the only product with an impressive 95% yield (50 g scale) and exceptional enantioselectivity (96% ee). Through an analogous synthetic route, the asymmetric synthesis of optically pure (−)-**706** (>99% ee) was also achieved starting from (−)-**703**. X-ray crystallography was employed to definitively determine the absolute configuration of the synthesized (−)-**706**. Subsequently, utilizing a similar pathway, the complete asymmetric synthesis of daphnillonin B was achieved starting from optically pure (−)-**706**.

### 4.22. Total Synthesis of Four Subfamilies of DAs by Li’s Group

In 2023, Li’s team announced the groundbreaking synthesis of four subfamilies of DAs: calyciphylline A-type, macrodaphniphyllamine-type, daphnilongeranin A-type, and daphnicyclidin D-type [233]. This achievement was made possible through an innovative biomimetic approach that incorporated substrate manipulation, reaction diversification, and pathway modification techniques.

**Scheme 50:**
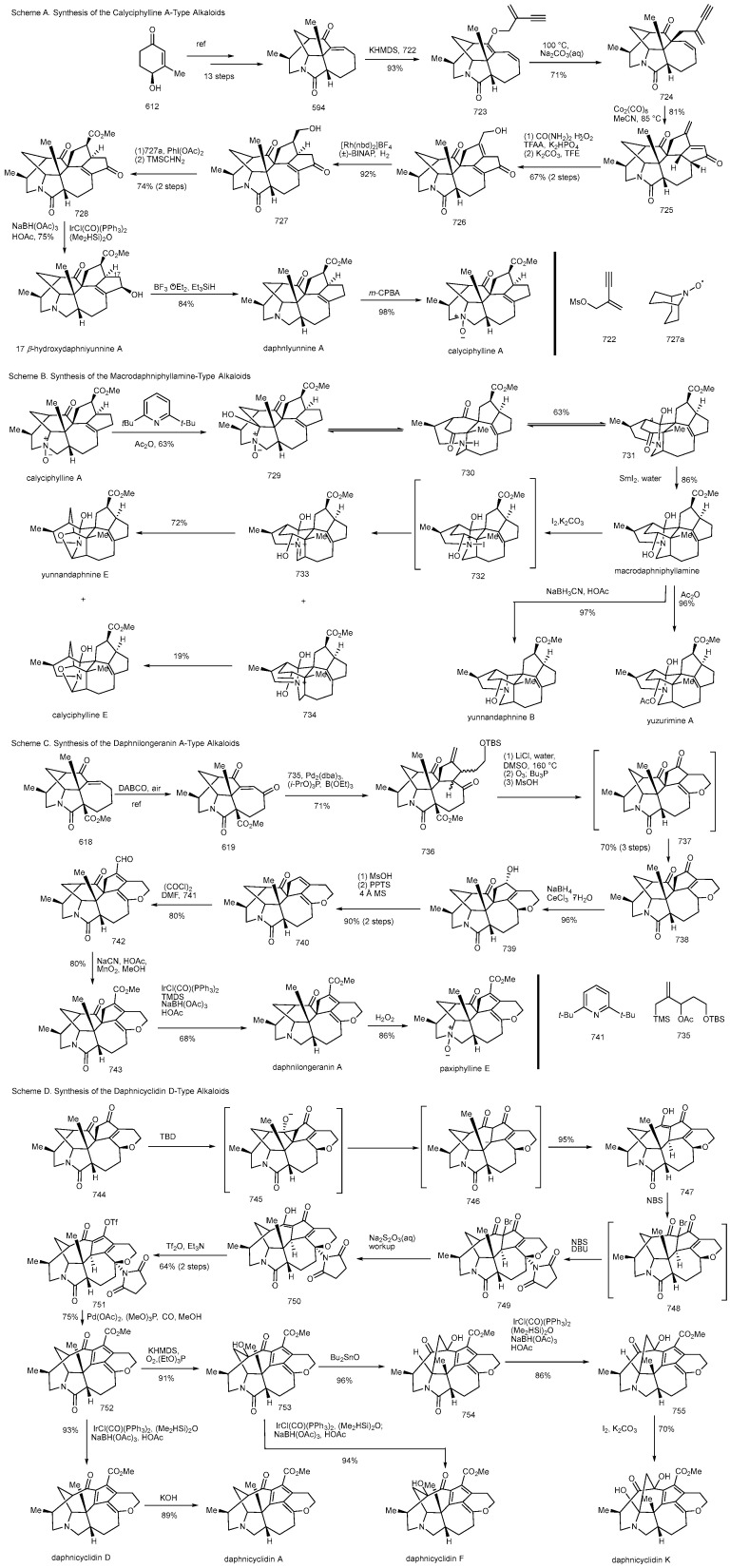
Total Synthesis of Four Subfamilies of DAs by A. Li’s group.

In their prior studies on DAs synthesis [145,164], A. Li’s group utilized tetracyclic compound **594**, which can be acquired from easily obtainable α,β-unsaturated enone **612**, as a crucial intermediate. As shown in Scheme 50A, by deprotonating **594** using KHMDS followed by *O*-allylation between the resulting enolate and mesylate **722**, the researchers achieved dienol ether **723** in excellent yield (93%). By adapting a procedure from their earlier work on the synthesis of hybridaphniphylline B [164], the researchers synthesized ketone **724** using Na_2_CO_3_(aq) in MeOH at 100 °C. Notably, exclusive formation of the C8 diastereomer was achieved. Upon treating the modified functionalized 1,6- synthesized enyne with Co_2_(CO)_8_ in MeCN at 85 °C via an intramolecular Pauson–Khand reaction, a significant majority (81%) of conjugated dienone **725** formed as a stereoisomer. The in situ generation of TFPAA epoxidized the exocyclic C=C bond of **725**, followed by a base-promoted cascade involving double bond migration and δ-alkoxy elimination to form hydroxy dienone **726**. By screening various catalysts, the researchers identified the optimal combination as [Rh(nbd)_2_]BF_4_(nbd = norbornadiene) and (±)-2,2′-bis(diphenylphosphino)-1,1′-binaphthyl ((±)-BINAP), which yielded **727**. To obtain the corresponding carboxylic acid (**727a**), the researchers oxidized the primary alcohol using 9-azabicyclo[3.3.1]nonane *N*-oxyl (ABNO) and PIDA, followed by methylation with TMSCHN_2_ in MeOH to produce ester **728** in a 74% overall yield. Under Nagashima conditions, the lactam in **728** was converted to the corresponding enamine, while the cyclopentenone experienced selective reduction from its convex face. By employing NaBH(OAc)_3_ in the presence of HOAc, the researchers obtained 17β-hydroxydaphniyunnine A through smooth reduction of the enamine. Subsequent deoxygenation of the resulting allylic alcohol [234] using Et_3_SiH and BF_3_·Et_2_O afforded daphniyunnine A an 84% yield. Further oxidation of daphniyunnine A using *m*-CPBA resulted in the formation of calyciphylline A in a 98% yield.

With a total of six compounds in their possession, the researchers devised a reverse synthetic pathway for macrodaphniphyllamine from calyciphylline A (Scheme 50B), aligning with the postulated biogenetic route. The primary obstacle during this procedure was the specific breaking of the C4–N bond in calyciphylline A. By utilizing Polonovski conditions (Ac_2_O and DTBMP), a mixture comprising hemiaminals **729** and **731** (~1.5:1) was obtained in 63% yield; it is presumed that **731** formed from **729** via diketone intermediate **730**. The SmI_2_/H_2_O reagent system was found to be the most effective for the production of macrodaphniphyllamine, resulting in an 86% yield. This alkaloid then served as a shared intermediate for synthesizing other members within the same subfamily. By selectively acetylating its C4 hydroxyl group using Ac_2_O, Et_3_N, and DMAP, yuzurimine A was obtained in high yield (96%). Additionally, yunnandaphnine B was produced in 97% yield by reducing the hemiaminal within macrodaphniphyllamine using NaBH_3_CN and HOAc. Additionally, the researchers utilized a dehydrogenation with mechanistic similarities to the Polonovski reaction for the one-pot synthesis of yunnandaphnine E and calyciphylline E from macrodaphniphyllamine. The reaction of macrodaphniphyllamine with I_2_ and K_2_CO_3_ produced a presumed quaternary ammonium intermediate, **732**. This intermediate then underwent different sequences involving HI elimination and hydroxyl attack, resulting in a 72% yield for yunnandaphnine E and a 19% yield for calyciphylline E. These products were formed via iminium ion species **733** and **734**, respectively.

As shown in Scheme 50C, the synthesis of enedione **619** was achieved by γ-oxidation of the readily accessible enone **618**. The Trost conditions were modified using tris(dibenzylideneacetone)dipalladium(0) (Pd_2_(dba)_3_), (*i*-PrO)_3_P (*i*-Pr = isopropyl), and triethyl borate (B(OEt)_3_) to transform the trimethylenemethane (TMM) precursor, **735**, into a mixture of C9 and C15 stereoisomers (**736**; approximately 13.6:5.5:2.5:1) in a total yield of 71%. Mixture **736** underwent a series of reactions, including Krapcho demethoxycarbonylation using LiCl, H_2_O, and DMSO at 160 °C; ozonolysis; and desilylative cyclization using methanesulfonic acid (MsOH). This led to the formation of α,β-unsaturated enone **738** with high overall efficiency. This outcome is likely caused by an in situ dehydration–double bond migration process involving an intermediate enol ether (**737**). The Luche reduction of **738** afforded allylic alcohol **739** as the sole diastereomer in an impressive 96% yield. Treating **739** with MsOH resulted in a blend of diene **740** and a δ-hydroxyketone, which was generated through hydrolytic processes. However, employing PPTS and a molecular sieve (4 Å) exclusively furnished **740** in 90% overall yield. By employing Vilsmeier–Haack reaction conditions, which included DMF, (COCl)_2_, and **741**, the electron-rich diene was transformed into aldehyde **742** in an impressive yield of 80%. Afterward, **742** was subjected to Corey oxidative esterification using NaCN, HOAc, MnO_2_, and MeOH, resulting in the successful synthesis of **743**. The lactam derivative was then subjected to Nagashima amide reduction using Vaska’s complex and TMDS, followed by enamine reduction with NaBH(OAc)_3_ and HOAc, resulting in the synthesis of daphnilongeranin A in 68% overall yield. Finally, oxidation of this tertiary amine using H_2_O_2_(aq) provided paxiphylline E with excellent yield (86%).

Exploiting the synthetic versatility of **744**, the researchers then established an expedited pathway to synthesize daphnicyclidin D (Scheme 50D). Drawing from their expertise in tandem reactions during the synthesis of daphenylline [144], the researchers discovered that TBD was an efficient reagent for initiating the cascade sequence. This is likely because it facilitates the intramolecular aldol reaction in **744**. The desired 5,7-fused bicyclic ring system was obtained as **747** as a sole C8 diastereomer in an impressive 85% yield. This outcome may have been achieved through intermediates **745** and **746**. Exposure to NBS at 55 °C predominantly resulted in bromide formation, as observed in intermediate **748**. Upon subsequent treatment with DBU and excess NBS under suitable conditions, intermediate **748** underwent sequential C10 bromination followed by nucleophilic substitution with the succinimide anion, thereby forming intermediate **749**. As anticipated, Na_2_S_2_O_3_(aq) workup resulted in the formation of enol **750**, which was subsequently triflated to afford **751** in 64% overall yield from starting material **747**. Modified Shair conditions (Pd(OAc)_2_, trimethyl phosphite ((MeO)_3_P), 90 bar CO, Et_3_N, and MeOH/DMSO) were employed for the methoxycarbonylation of **751**. Additionally, in a weakly basic environment, the increased acidity of the cyclopentadiene moiety led to the spontaneous elimination of the succinimide. As a result, methyl ester **752** was directly obtained in satisfactory yield (75%).

From **752**, daphnicyclidin D achieved an impressive yield (93%) through one-pot lactam reduction, as mentioned above. In addition, taking inspiration from the polarized nature of its fulvene domain, which incorporates two electron-withdrawing groups, the researchers devised a sequential approach involving KOH-mediated conjugate addition–lactol ring opening–lactonization. This strategy enabled the synthesis of daphnicyclidin A in high yield (89%). In another pathway, **752** was converted to **753**. Reacting **753** with a small quantity of dibutyltin oxide (Bu_2_SnO) at 80 °C led to the synthesis of **754** in exceptional yield (96%). The lactam reduction of **754** yielded *proto*-daphnicyclidin K (**755**), which is potentially an as-yet-undiscovered naturally occurring DA. Treatment of this tertiary amine with I_2_ and K_2_CO_3_ afforded daphnicyclidin K in 70% yield. Finally, the lactam reduction of **753** achieved the efficient synthesis of daphnicyclidin F.

## 5. Conclusions

To date, over 350 DAs have been isolated and reported. However, the number of new isolations has decreased significantly in recent years, largely because of previous intensive efforts. Despite this decline in new discoveries, the diverse structural and bioactive properties of DAs continue to capture the attention of organic and synthetic chemists [235,236,237,238]. In particular, over the past five years, several studies have been conducted on the total synthesis of DAs. A range of innovative approaches have been devised to facilitate the efficient and streamlined production of DAs. These efforts have not only advanced the synthesis of DAs but have also contributed to the broader development of organic synthesis methodologies. As the strategy for synthesizing DAs is becoming more and more concise, it helps to accumulate a large number of molecular samples of the alkaloids, which can be applied to biological activity tests and then discover more interesting activities and even applied to clinical trials.

Future research on DA synthesis should focus on the construction of polycyclic skeleton systems and asymmetric synthesis techniques, particularly those catalyzed by metal complexes or small organic molecules. As novel synthesis methodologies and experimental technologies continue to advance, we can expect an increasing number of DA syntheses to be reported in the future.

## Data Availability

Not applicable.
